# Transfer of clinically relevant gene expression signatures in breast cancer: from Affymetrix microarray to Illumina RNA-Sequencing technology

**DOI:** 10.1186/1471-2164-15-1008

**Published:** 2014-11-21

**Authors:** Debora Fumagalli, Alexis Blanchet-Cohen, David Brown, Christine Desmedt, David Gacquer, Stefan Michiels, Françoise Rothé, Samira Majjaj, Roberto Salgado, Denis Larsimont, Michail Ignatiadis, Marion Maetens, Martine Piccart, Vincent Detours, Christos Sotiriou, Benjamin Haibe-Kains

**Affiliations:** Breast Cancer Translational Research Laboratory (BCTL), Institut Jules Bordet, Brussels, Belgium; Bioinformatics Core Facility, Institut de Recherches cliniques de Montréal, Montreal, QC Canada; IRIBHM, Université Libre de Bruxelles, Campus Erasme, Brussels, Belgium; Department of Biostatistics and Epidemiology, Institut Gustave-Roussy, Villejuif, France; Paris-Sud University, Paris, France; Breast International Group, Brussels, Belgium; Department of Pathology, Institut Jules Bordet, Brussels, Belgium; Princess Margaret Cancer Centre, University Health Network, Toronto, ON Canada; Medical Biophysics Department, University of Toronto, Toronto, ON Canada

**Keywords:** Breast cancer, Gene expression signatures, Affymetrix, Microarray, Illumina, RNA-Seq, Immunohistochemistry, Estrogen receptor, Progesterone receptor, HER2 receptor

## Abstract

**Background:**

Microarrays have revolutionized breast cancer (BC) research by enabling studies of gene expression on a transcriptome-wide scale. Recently, RNA-Sequencing (RNA-Seq) has emerged as an alternative for precise readouts of the transcriptome. To date, no study has compared the ability of the two technologies to quantify clinically relevant individual genes and microarray-derived gene expression signatures (GES) in a set of BC samples encompassing the known molecular BC’s subtypes. To accomplish this, the RNA from 57 BCs representing the four main molecular subtypes (triple negative, HER2 positive, luminal A, luminal B), was profiled with Affymetrix HG-U133 Plus 2.0 chips and sequenced using the Illumina HiSeq 2000 platform. The correlations of three clinically relevant BC genes, six molecular subtype classifiers, and a selection of 21 GES were evaluated.

**Results:**

16,097 genes common to the two platforms were retained for downstream analysis. Gene-wise comparison of microarray and RNA-Seq data revealed that 52% had a Spearman’s correlation coefficient greater than 0.7 with highly correlated genes displaying significantly higher expression levels. We found excellent correlation between microarray and RNA-Seq for the estrogen receptor (ER; r_s_ = 0.973; 95% CI: 0.971-0.975), progesterone receptor (PgR; r_s_ = 0.95; 0.947-0.954), and human epidermal growth factor receptor 2 (HER2; r_s_ = 0.918; 0.912-0.923), while a few discordances between ER and PgR quantified by immunohistochemistry and RNA-Seq/microarray were observed. All the subtype classifiers evaluated agreed well (Cohen’s kappa coefficients >0.8) and all the proliferation-based GES showed excellent Spearman correlations between microarray and RNA-Seq (all r_s_ >0.965). Immune-, stroma- and pathway-based GES showed a lower correlation relative to prognostic signatures (all r_s_ >0.6).

**Conclusions:**

To our knowledge, this is the first study to report a systematic comparison of RNA-Seq to microarray for the evaluation of single genes and GES clinically relevant to BC. According to our results, the vast majority of single gene biomarkers and well-established GES can be reliably evaluated using the RNA-Seq technology.

**Electronic supplementary material:**

The online version of this article (doi:10.1186/1471-2164-15-1008) contains supplementary material, which is available to authorized users.

## Background

For more than a decade, microarrays have represented the most comprehensive approach to measuring gene expression levels [1]. Their ability to simultaneously assess thousands of transcripts, coupled with relatively low experimentation costs and the broad availability of analytical tools, have facilitated their wide use and led to fundamental advances in several research fields. In breast cancer, implementing gene expression microarrays has broadened our knowledge about the biology of the disease, which has, for many years, relied on immunohistochemistry (IHC) and clinical-pathologic parameters only. Several studies have shown that breast cancers can be classified into at least four “intrinsic” subtypes (basal-like, HER2 enriched, luminal A, and luminal B) which can only be partially recapitulated by IHC definitions of the three fundamental breast cancer biomarkers: estrogen receptor (ER), progesterone receptor (PgR), and human epidermal growth factor receptor 2 (HER2) [2–5].

In current clinical practice, subtypes are defined mostly by using an IHC surrogate [6]. Multiple expression-based classifiers have been developed, including two versions of the Subtype Classification Model (SCM) (SCMOD1 [7] and SCMOD2 [8]), and the simple three-gene model (SCMGENE [9]) developed by Sotiriou and co-workers, as well as three variants of the Single Sample Predictor (SSP) (SSP2003 [10], SSP2006 [11] and PAM50 [12]) developed by Perou and co-workers, the latter having been recently translated into a clinical assay (Prosigna^™^[13]). The computational implementation and comparison of these classifiers has been documented in [9]. Microarrays have also been used to derive a series of gene expression signatures aimed at characterizing the biology of the disease and at helping clinicians predict relapse and response to treatment more accurately than tools using traditional clinico-pathological parameters [7, 12, 14–27]. Some of these signatures have been endorsed by international breast cancer guidelines, and they are being increasingly implemented in standard practice [6].

In parallel, RNA sequencing (RNA-Seq) is emerging as the technology of reference for thorough characterization of the human transcriptome and as a superior alternative to microarrays to define gene expression levels [28–31]. RNA-Seq is overcoming some of the drawbacks of microarrays [28–32]. For instance, the dependence of microarrays on hybridization of transcripts to pre-determined probes restricts analysis to genes for which genomic sequence information is available first-hand and to sequences that are distant enough so that probes do not cross-hybridize. Moreover, high levels of background noise arising from non-specific hybridization and probe saturation affect the quantification of transcripts expressed at low and high levels, respectively, limiting the dynamic range of the technology. Although the RNA-Seq technology efficiently address these issues, the current lack of standards for analyzing these new data, coupled with the relatively high cost of the RNA-Seq experiments, could deter investigators from implementing the technology in their activity.

Several studies have been carried out to compare the performance of RNA-Seq and microarrays, including exon arrays, in defining levels of gene expression [33–42]. The vast majority of them have focused on establishing the reliability of RNA-Seq in differential gene expression (DGE) analyses between two or more samples and/or conditions of interest. Despite the fact that they have generally reported good correlation between the two technologies, most of these studies have relied on relatively few samples or exclusively on non-human samples. Moreover, they have never attempted to assess the performance of RNA-Seq in defining clinically relevant biomarkers developed using microarrays. Given the promise of microarray-based gene signatures [43] and the significant advantages of the new RNA-Seq technology in providing more accurate and reliable gene expression measurements, there is a dire need to investigate the transition of breast cancer gene signatures from microarray to RNA-Seq.

The main aim of the current study is to compare the agreement between two of the most widely used microarray and RNA-Seq platforms, Affymetrix and Illumina HiSeq respectively, in estimating (1) the expression of single genes which are clinically relevant to breast cancer and (2) breast cancer subtype classifiers and gene expression signatures that have been developed over the years with microarrays. The comparison uses a dataset obtained from well characterized breast cancer patients representing the four main breast cancer subtypes: triple negative, HER2 positive, luminal A, and luminal B.

## Methods

### Sample selection and characterization

Fresh-frozen tumor material was obtained from 57 breast cancer patients who were treated at Institut Jules Bordet (Brussels, Belgium) between 2007 and 2011 and whose samples were stored at the institute’s biorepository. The samples represented the four main known breast cancer subtypes (by IHC) and their tumor cell content evaluated on a hematoxylin-eosin (H&E) slide by a board-certified breast cancer pathologist was greater than or equal to 30%. Of the 57 patients, 17 had triple negative breast cancer (TN: ER, PgR, and HER2 negative), 14 HER2 positive (any ER and PgR, HER2 positive), 16 luminal A (ER, HER2 negative, histological grade 1), and 10 luminal B (ER, HER2 negative, histological grade 3). The use of the tumor material is consistent with the informed consent signed by the patients and was granted approval by Institut Jules Bordet’s ethics committee (approval number: CE1967), and is in accordance with the applicable laws and regulations in Belgium.

### RNA extraction

RNA was extracted using TRIzol® (Life Technologies, Carlsbad, California) following the manufacturer’s instructions. Concentration was measured using the NanoDrop 1000 (Thermo Scientific, Waltham, Massachusetts), and integrity (RIN: RNA Integrity Number) was assessed using an Agilent 2100 Bioanalyzer (Agilent Technologies, Santa Clara, California). All the samples yielded enough material for downstream experiments, and had a RIN equal to or greater than 6.5. RNA obtained from the same extraction procedure was profiled on microarrays and sequenced on the Illumina HiSeq 2000.

### Microarray experiments

100 ng of total RNA was profiled at the Institut Jules Bordet using the Affymetrix® HG-U133 Plus 2.0 Arrays (Affymetrix, Santa Clara, California), following the manufacturer’s instructions. Briefly, the RNA was first reverse-transcribed into double-stranded cDNA. This cDNA was transcribed in vitro. After purification of the aRNA, 12.5 μg were fragmented and labeled prior to hybridization to the chips. Quality control (QC) for each chip was performed following the recommendations posted on [44]. Following QC, the probe level intensities were background adjusted and quantile normalized using the Frozen Robust Multiarray Analysis (fRMA) method [45] as implemented in the R/Bioconductor package fmra [46]. Probeset level annotations were obtained from R/Bioconductor package jetset [47] and complemented with BioMart [48]; when multiple probesets mapped to the same Entrez Gene ID, the probeset with the highest jetset score was selected. The raw Affymetrix CEL files are available from the NCBI’s Gene Expression Omnibus under accession number GSE43358.

The data can be accessed through this link: http://www.ncbi.nlm.nih.gov/geo/query/acc.cgi?token=trmzbecoaqyugtc&acc=GSE43358.

### RNA-Seq sample preparation and sequencing

Transcriptome sequencing was performed at DNAVision (Gosselies, Belgium). Transcriptome libraries were constructed using the Illumina ® TruSeq™ RNA Sample Preparation Kit for paired-end reads sequencing on the HiSeq 2000 (Illumina, San Diego, California) following the manufacturer’s instructions. Briefly, starting from 1 μg of total RNA, the poly-A containing mRNA molecules were purified using poly-T oligo-attached magnetic beads. Following purification, the mRNA was fragmented into small pieces using divalent cations under elevated temperature. The cleaved RNA fragments were copied into first strand cDNA using reverse transcriptase and random primers. This was followed by second strand cDNA synthesis using DNA Polymerase I and RNase H and purification using the AMPure XP beads (Agencourt BioSciences Corporation, Beverly, Massachusetts). The cDNA fragments were end repaired with the addition of a single ‘A’ base and the ligation of adapters. The products were purified using the AMPure XP beads and enriched with PCR (15 cycles) to create the final cDNA library followed by purification using the AMPure XP beads. Library quality control and quantification were performed using the Agilent Bioanalyser 2100 and qRT-PCR. The libraries were then pooled (4 libraries/pool). Clusters were generated in a cBot Cluster Generation System using the Paired-End Cluster Generation Kit v2-HS and sequenced on the Illumina® HiSeq 2000 platform with a 2x50 base-pairs paired-end mode. Base calls were made using the Illumina CASAVA 1.5 pipeline. Sequence data has been deposited at the European Genome-phenome Archive (EGA), which is hosted by the EBI and the CRG [49], under study’s accession number EGAS00001000495 and dataset’s accession number EGAD00001000626.

### Assessment of RNA-Seq data quality

The following statistics were computed to verify the quality of the RNA-Seq data (Additional file 1: Table S1). The total number of paired reads, the average Phred quality scores, and the average GC content were calculated on the quality trimmed FASTQ files with FastQC [50]. The percentage of “proper pairs”, defined as mapped paired reads with an insert size ranging from 60 to 160 bp, was calculated with BamTools [51]. The percentage of aligned duplicate read pairs was calculated with Picard tools MarkDuplicates [52].

### RNA-Seq analysis

After trimming the poor quality bases, the reads were mapped to the human reference genome hg19 with TopHat2 (version 2.0.0) [53], and gene expression was quantified with Cufflinks (version 2.0.0) [53]. The annotation file (GTF file) used for both alignment and gene quantification was downloaded from Ensembl (on 26 July, 2012). To match the log scale of gene expression measurements from the microarray data, the FPKM (Fragments Per Kilobase of transcript per Million mapped reads) values computed by Cufflinks were log-transformed using the following formula:


where X represents the FPKM value computed by Cufflinks, and X’ is the log-transformed expression value.

### Gene expression signatures

Our study focused on the following gene expression signatures: (1) six prognostic signatures (GENE70 [14], GENE21 [15], Genomic Grade Index (GGI [16]), Risk of Relapse-Score (ROR-S [12]), ENDOPREDICT [17], and CIN70 [18]); (2) two immune signatures (STAT1MODULE [7] and IRMODULE [19]); (3) three stroma-related signatures (PLAUMODULE [7], DCN [20], and STROMACD10 [21]); and (4) ten pathway related signatures (PIK3CA-GS [22], PTEN loss [23], IGF1 [24], AKT/mTOR [25], MAPK [26], SRC, RAS, MYC, E2F3, and beta-catenin [27]). We also evaluated six subtype classifiers: SCMOD1 [7], SCMOD2 [8], SCMGENE [9], SSP2003 [10], SSP2006 [11], and PAM50 [12]. In addition to these multivariate subtyping models, we evaluated the three individual breast cancer clinically relevant genes: ER, PgR, and HER2.

We used the original signature algorithms for GENE70, GENE21, GGI, ROR-S, ENDOPREDICT, CIN70, STAT1MODULE, PLAUMODULE, DCN and STROMACD10 as implemented in the Bioconductor package *genefu*[54]. For the remaining gene expression signatures, we computed the signature scores following the approach used in Ignatiadis et al. [55]. The scores were computed from the list of genes in their respective signatures (as listed in GeneSigDB [56]) as the weighted average using the following formula:


where *s* is the signature score, *n* is the number of genes in the signature of interest, *x*_*i*_ is the expression of the gene, and the gene-specific weight *w*_*i*_ ∈ {−1,1} is the sign of the coefficient defined in the original publication. Only genes that could be mapped to EntrezGene IDs were used. Finally, each signature score was rescaled so that the 2.5% and 97.5% quantiles were equal to +1 and −1 respectively.

### Data analysis

The pair-wise correlation between Affymetrix microarrays and Illumina RNA-Seq gene expression data and gene expression signatures scores was assessed using Spearman’s rank-based correlation. For the three single gene biomarkers (ER, PgR and HER2), the correlation between microarray or RNA-Seq with IHC was estimated to identify which technology provided better concordance with IHC. Cohen’s kappa coefficient was used to compare the subtype classifications from microarray or RNA-Seq data. To statistically compare the Spearman correlation and Cohen’s kappa coefficients of different gene signatures, we used a two-sided Wilcoxon rank sum test with 100 bootstrap replicates of the 57 patients to determine the p-value. The resulting p-values, reporting the significance of the correlation difference between each pair of gene expression signatures, were corrected for multiple testing using Bonferroni’s method.

To compare the correlation of gene expression over the whole transcriptome between each pair of data type from a given sample, we used Spearman’s rank-based correlation, the null distribution of which was established as the range of coefficients observed from all possible combinations of the 57 pairs excluding self-self pairs. This is efficiently computed from the cross correlation matrix minus the diagonal elements. The analyses performed in this study are fully reproducible and comply with proposed guidelines in terms of availability of the code and data [57]. The R scripts developed for the analysis are available upon request.

### Data availability

The raw Affymetrix CEL files are available from the NCBI’s Gene Expression Omnibus under accession number GSE43358. The data can be accessed through this link: http://www.ncbi.nlm.nih.gov/geo/query/acc.cgi?token=trmzbecoaqyugtc&acc=GSE43358.

Sequence data has been deposited at the European Genome-phenome Archive (EGA), which is hosted by the EBI and the CRG [49], under study’s accession number EGAS00001000495 and dataset’s accession number EGAD00001000626.

## Results

### Gene-wise comparison of expression levels using Affymetrix microarray and Illumina RNA-Seq platforms

**Figure 1:**
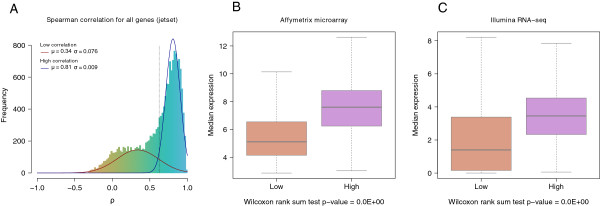
**Gene expression correlation between Affymetrix microarray and Illumina RNA-Seq platforms. A:** Expression correlation of the 16,097 genes measured both on Affymetrix microarray and Illumina RNA-Seq platforms after selecting the best Affymetrix probeset using jetset. **B:** and **C:** Box plots showing median level of gene expression for both Affymetrix and RNA-Seq for the genes with low (<0.7) and high (≥0.7) correlation. Genes highly correlated between the two platforms showed higher levels of expression than those with low correlation.

A subset of 16,097 genes were defined as common to the two platforms and retained for downstream analysis. Gene identifiers did not perfectly overlap due to differences in the annotation systems: jetset matched the Affymetrix probesets to the NCBI RefSeq human cDNA database, while the RNA-Seq analysis pipeline used Ensembl gene annotations (see Methods for more detail). When comparing the expression levels of the genes retained after selection of the best Affymetrix probeset, we found that although the scale of expression values differs due to different technology and normalization procedures, their rank is well conserved with 52%, 34%, and 11% of these genes having Spearman’s rank-based correlation greater than 0.7, 0.8, and 0.9, respectively (Figure 1A). The Spearman correlation coefficient for each evaluated gene is reported in Additional file 1: Table S2.

We observed that genes with the highest correlation coefficients for the comparison of microarray and RNA-Seq were significantly more expressed. Similarly, the genes that were positively correlated were significantly more expressed than genes that were negatively correlated (two-sided Wilcoxon rank sum test p-value <1x10^−16^; Additional file 2: Figure S1). This result holds true when considering a high cutoff of correlation r_s_ ≥0.7 (two-sided Wilcoxon rank sum test p-value <1x10^−16^; Figure 1B and 1C). This phenomenon could be explained by the potentially higher variance of genes expressed at low levels [58] or by the fact that microarrays have a limited dynamic range compared to RNA-Seq [28–32].

In order to investigate this phenomenon more deeply, we computed the correlation for genes with cumulative increasing expression (Additional file 3: Figure S2). By starting with genes expressed at low levels and increasing gene expression levels by increments of 1%, we observed a steep rise in the median correlation coefficient; in contrast, the magnitude of the decrease moving away from genes expressed at high levels was much lower. These results suggest that, despite the potential saturation of microarrays for highly expressed genes, the correlation between the two technologies remains high; however, we observed a high-level of inconsistency for genes expressed at low levels, either originating from microarray or RNA-seq technology, or both.

We acknowledge from past experience with gene expression microarrays that, when comparing the whole transcriptome, two unrelated samples may have a correlation coefficient that is as high as two arrays performed on the same sample, raising questions about the significance of an asymptotic p-value in that particular setting. In our dataset, the correlation of gene expression profiles for the whole transcriptome measured by microarray and RNA-Seq was statistically significant for all except three patients (Additional file 4: Figure S3).

### Definition of ER, PgR, and HER2 status according to IHC, microarray, and RNA-Seq

**Figure 2:**
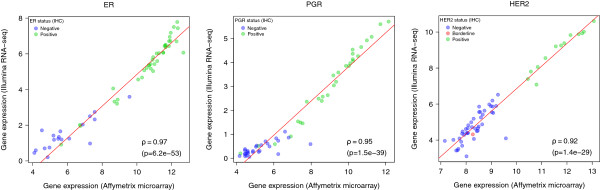
**Expression correlation for ER, PgR, and HER2 genes.** Scatterplots reporting the expression correlation of ER, PgR, and HER2 defined by Affymetrix microarray or Illumina RNA-Seq. Each dot is colored according to the corresponding status determined by IHC: green for positive, blue for negative, red for borderline. Spearman correlation coefficient and p-value are provided below the plots.

Among the genes retained for analysis, we focused our attention on three that are clinically relevant for breast cancer: ER, PgR, and HER2. Measuring them precisely is of utmost importance to clinical practice as these are presently the only validated breast cancer predictive biomarkers available, and they are routinely used to make decisions about patient treatment [6, 59].When comparing the expression levels of these three genes as defined by microarray and RNA-Seq, we found excellent Spearman correlation coefficients: 0.973 for ER [95% CI: 0.971-0.975]; 0.95 for PgR [95% CI: 0.947-0.954]; and 0.918 for HER2 [95% CI: 0.912-0.923] (Figure 2).

We then went a step further and compared the gene expression levels defined by either RNA-Seq or Affymetrix with IHC, which is currently considered to be the methodology of reference for the definition of these markers, together with FISH for HER2 [60, 61]. Overall, a good correlation was found between the technologies (r_s_ >0.69), and only a few discordances were observed (Additional file 5: Figure S4).

### Correlation between technologies for the definition of breast cancer subtype classifiers

**Figure 3:**
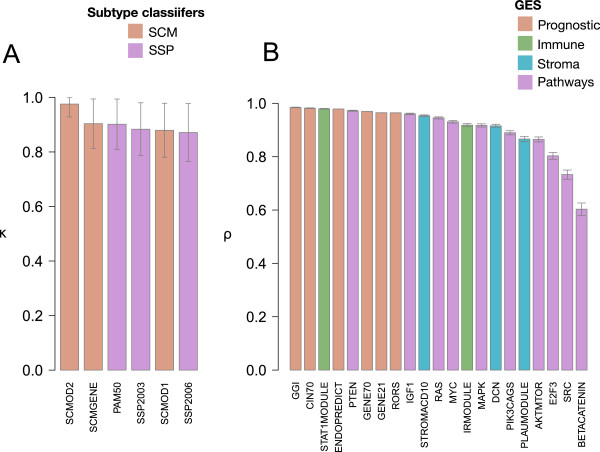
**Correlation values for the evaluated subtype classifiers and gene expression signatures. A:** Cohen’s Kappa coefficients for subtype classifiers (orange: SCMs; purple: SSPs). **B:** Spearman correlation values for prognostic (orange), immune (green), stroma (blue) and pathway (purple) signature scores as computed using Affymetrix microarray and Illumina RNA-Seq platforms.

Two different gene expression approaches have been developed to prospectively classify breast cancers into molecular subtypes: Subtype Classification Models (SCMs) [7–9] and Single Sample Predictors (SSPs) [10–12], which include PAM50. In the current dataset, our subtype classifier SCMOD2 [8] showed the highest correlation between microarray and RNA-Seq technologies (κ = 0.975; Figure 3A, Additional file 1: Table S3), which was significantly higher than the other classifiers (100 bootstrap replicates, corrected p-value <0.001; Additional file 1: Table S4). Of note, although the kappa coefficients for SCMGENE [9] and PAM50 [12] were very similar (κ = 0.903 vs. 0.902 for SCMGENE and PAM50, respectively), SCMGENE was more concordant than PAM50 in our study (corrected p-value = 0.001, Additional file 1: Table S4).

### Correlation between technologies: gene prognostic signatures

**Figure 4:**
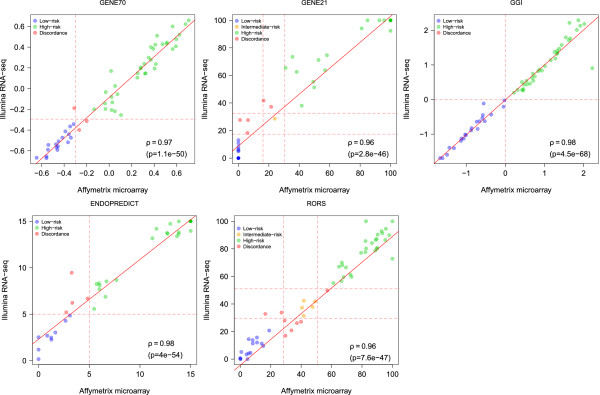
**Risk prediction scores of the commercially available prognostic signatures.** Scatterplots reporting the continuous risk prediction scores of the commercially available prognostic signatures. Each dot is colored according to the corresponding risk classification: blue for concordant low-risk, orange for concordant intermediate risk, green for concordant high-risk and red for discordance. The cutoff used to discretize the continuous risk predictions into risk classifications are represented in dashed red lines. Spearman correlation coefficient and p-value are provided below the plots.

Using microarray technology, several prognostic gene expression signatures have been developed in the attempt to help clinicians to identify which breast cancers are at high or low risk of recurrence [43]. Among these, MammaPrint® (here referred to as GENE70) [14], OncotypeDx® (here referred to as GENE21) [15], GGI [16], ENDOPREDICT [17] and ROR-S [12] have been widely investigated and applied in the clinical setting. When comparing the values of these signatures on a continuum as defined by either microarray or RNA-Seq, an excellent Spearman correlation was found: 0.97 [95% CI 0.968-0.972] for GENE70; 0.965 [95% CI 0.962-0.967] for GENE21; 0.985 [95% CI 0.984-0.986] for GGI; 0.979 [95%CI 0.977,0.981] for ENDOPREDICT; and 0.965 (95% CI 0.962,0.967) for ROR-S (Figure 4).

### Correlation between technologies: immune, stroma and pathway related gene expression signatures

After a first wave of prognostic signatures, which essentially captured tumor proliferation signals, a new generation of “biological” signatures were developed that focused on determining the prognostic and/or predictive role of host-tumor immune interplay, tumor microenvironment and pathway activation signaling. We evaluated the correlation between microarray and RNA-Seq platforms in measuring the following: 1) two immune signatures (STAT1MODULE [7], IRMODULE [19]); 2) three stroma signatures (PLAUMODULE [7], DCN [20], STROMACD10 [21]); and 3) ten pathway signatures (PIK3CA-GS [22], PTEN loss [23], IGF1 [24], AKT/mTOR [25], MAPK [26], SRC, RAS, MYC, E2F3, beta-catenin [27]).As shown in Figure 3B, the Spearman correlation coefficients were better overall for the prognostic signatures than the biological ones (corrected p-value <0.001). This was particularly the case for the pathway signatures.

### Signature enrichment in highly expressed genes and correlation between technologies

Since higher correlation coefficients were found for genes expressed at higher levels, we investigated whether enrichment in genes with higher expression in the above subtype classifiers and gene expression signatures could have affected their correlation when defined with the two technologies. We found that 81% (22/27) and 74% (20/27) of the signatures were significantly enriched with highly expressed genes for Affymetrix microarray and Illumina RNA-seq platforms, respectively (p-value <0.05; Additional file 6: Figure S5 and Additional file 7: Figure S6). However such enrichment did not imply significantly higher correlation between the two platforms, suggesting that the proportion of highly expressed genes is not solely responsible for the highest correlations observed in our study.

## Discussion

The use of molecular tools in clinical practice is on the rise. In breast cancer, international guidelines endorse the implementation of microarray-derived gene signatures to support clinicians in the treatment decision-making process [6]. In addition, the upcoming results of two genome-forward clinical trials, namely the MINDACT and the TailorX trials [62, 63], involving thousands of patients, will provide the highest level of evidence to date about whether basing clinical decisions on microarray-derived prognostic gene signatures influences the outcome of breast cancer patients. The parallel rise of RNA-Seq as an accurate and reliable alternative to microarrays for transcriptome characterization and gene expression quantification is puzzling for investigators, who must decide which technology best fulfills their clinical needs. Hence, it is imperative to determine the reliability of transitioning from microarray to sequencing platforms in the clinical setting. To our knowledge, our is the first report investigating the correlation between the expression level of single clinically relevant genes and gene expression signatures obtained with the most commonly used microarray and RNA-Seq platforms, Affymetrix and Illumina respectively, in a selected dataset of breast cancer patients.

When focusing on the genes in common between the two platforms, our analysis showed that the expression of more than half of them had a Spearman correlation of 0.7. This was the case simply by selecting the most specific Affymetrix probeset and correlating it to gene log2 transformed FPKM obtained at sequencing, without any further computation. In studies exploring the correlation of the two technologies in defining genes differentially expressed among samples and/or conditions, the correlation values of fold change ratios were reported to range from 0.55 to 0.85 [33–42]. Of note, these values are similar to or higher than the correlation observed across different microarray platforms [64]. In these same studies, RNA-Seq seemed to be more reliable than microarray in identifying differential gene expression: in several reports, a large proportion of genes identified as differentially expressed by RNA-Seq but not by microarray were in fact confirmed by qPCR [33, 34, 65]. RNA-Seq also seems more suitable than microarrays to quantify absolute gene expression levels when compared with mass spectrometry measurements [35].

As reported by other investigators [28, 35, 65], we found that the correlation of genes was higher when their expression levels were higher. The reliance of microarrays on the presence of primers and probes limits their ability to measure extreme gene expressions; according to our data, the limitation seems to be more relevant in the low expression range. On the contrary, the nature of sequencing technologies allows the unbiased investigation of gene expression when enough depth is assured.

In our study, RNA-Seq showed high correlation with microarrays when measuring clinically relevant genes in breast cancer (i.e., ER, PgR, and HER2). Nevertheless, despite an overall good correlation, both technologies showed a few discordances when compared to IHC. Similar results have already been reported when comparing microarray with IHC [66, 67]. These discordances might be partly attributed to the fact that IHC measures the expression of ER, PgR, and HER2 at the protein level, while both Affymetrix and RNA-Seq measure the expression levels of the corresponding mRNAs. Although mRNA and protein expression levels are not fully independent, one cannot exclude that post-transcriptional regulation might affect their correlation. Another factor that could account for these discordances is tumor heterogeneity. The expression levels of the three markers can in fact vary across different areas of the tumor. While the RNA profiled on microarray and sequenced on the Illumina platform was obtained from the same tumor area, the slides used for IHC staining were cut from a distinct area of the tumor lesion. The best way to capture the activity level of these receptors and their downstream pathways remains largely unclear. It is possible that the determination of ER, PgR, and HER2 status at the mRNA level could provide clinicians with a more reproducible, quantitative, and informative assessment of these markers [68, 69]. For the time being, however, not enough evidence is available to recommend mRNA-based measurements for clinical practice.

Because of the clinical relevance of breast cancer subtypes, much attention has been paid to the performance of microarray-derived subtype classifiers. Some concerns have been raised about their reliability though. It has been shown that although every SSP can consistently identify molecular subtypes with different levels of survival, they do not reliably assign the same patients to the same molecular subtype [70]. Variation in gene expression data and classification algorithms could influence how samples are classified into each subtype. In our study, the best correlation between microarray and RNA-Seq was obtained for SCMOD2 [8], one of the subtype classifiers developed by our group. Similar correlation coefficients were obtained for PAM50 [12] and SCMGENE [9], with the latter showing slightly higher concordance. This result suggests the higher robustness of SCMGENE to small perturbations, which concurs with our recent robustness study [9]. However, it is unlikely that Prosigna^™^, the recent clinical assay implementing PAM50 using the NanoString® platform, would suffer the same limitation since its analytical validity has been thoroughly assessed [13].

Given the increasing implementation of microarray-derived gene signatures in clinical practice, it is vital to determine if RNA-Seq could reliably be used to define them. When considering 21 of the most relevant signatures developed in recent years [7, 12, 14–27], we found that the correlation values for microarray and RNA-Seq for signature determination ranged from moderate to very strong. Correlation values were higher for prognostic signatures than biological signatures, independently from their enrichment in highly expressed genes. This suggests that proliferation, which drives prognostic signatures, is more robust and reproducible than signals coming from other biological processes and that complex signatures developed on microarrays might be less stable.

## Conclusions

In our study, we have demonstrated that RNA-Seq can reliably be used to evaluate both the expression of clinically relevant single genes and well established gene expression signatures originally defined with microarray technology. Considering the advantages that RNA-Seq offers over microarray, such as its ability to explore larger sets of genes, it is envisaged that its application to wider datasets could even provide information relevant for de-novo classification of breast cancer or for the development of new prognostic and predictive signatures. With the cost of RNA-Seq experiments decreasing continuously and with well-established analytical tools increasingly available [71–75], RNA-Seq is becoming an accessible tool that is superseding microarray in the research setting. We foresee that, with the aid of initiatives such as the SEquencing Quality Control (SEQC) consortium (the third phase of the FDA-led MAQC project) [76] and studies such as ours confirming its reliability, RNA-Seq will eventually also supersede microarrays in the clinical setting.

## Electronic supplementary material

Additional file 1: **Table S1.** Read statistics. “Proper pairs” are defined as reads where both pairs map to the reference genome with an inner distance between 60 and 160 bases. **Table S2.** Spearman correlation coefficients for each gene considered for comparison between Affymetrix microarray and Illumina RNA-Seq platforms. **Table S3.** Contingency table for breast cancer subtype classifiers (SCMs and SSPs) using Affymetrix microarray (AFFY) and Illumina RNA-Seq (RNASEQ) platforms. **Table S4.** Bootstrap analysis to assess the significance of the differences observed for Cohen’s Kappa coefficients for subtype classifiers using Affymetrix microarray and Illumina RNA-Seq platforms. (XLS 6 MB)

Additional file 2: Figure S1: A and B: Box plots showing median level of expression on both Affymetrix microarray and Illumina RNA-Seq platforms for negatively and positively correlated genes. (PDF 9 KB)

Additional file 3: Figure S2: Correlation of gene expression levels between Affymetrix microarray and Illumina RNA-Seq platforms with respect to increasing quantiles of gene expression. (PDF 30 KB)

Additional file 4: Figure S3: Scatterplots reporting the point-to-point comparison of gene expression profiles measured by Affymetrix microarray and Illumina RNA-Seq platforms for each individual patient considered in the current study. Spearman correlation coefficient and p-value are provided below the plots. The solid line represents a linear regression of microarray values on the RNA-seq data while the dotted line has the equation y = x. (PDF 757 KB)

Additional file 5: Figure S4: Spearman correlation for the quantification of three clinically relevant genes (ER, PgR, and HER2) as defined by IHC vs Affymetrix microarray and Illumina RNA-Seq, respectively. (PDF 37 KB)

Additional file 6: Figure S5: Bar plots representing the proportion of genes present in all signatures combined (3,663 unique genes in 27 signatures) with respect to their quantiles of expression for Affymetrix microarray (blue) and Illumina RNA-seq (red) platforms. The p-value reports the significance of the enrichment of signature genes with increasing quantiles of expression (Spearman’s rank-based correlation). (PDF 9 KB)

Additional file 7: Figure S6: Bar plots representing, for each signature (27), the proportion of genes present in all signatures combined with respect to their quantiles of expression for Affymetrix microarray (blue) and Illumina RNA-seq (red) platforms. The p-value reports the significance of the enrichment of signature genes with increasing quantiles of expression (Spearman’s rank-based correlation). Note that the SCMGENE and IRMODULE signatures contains few genes (3 and 6 genes, respectively) while the median signature size is 95 genes; for these small signatures, the p-value is expected to be large due to reduce sample size for the correlation analysis. (PDF 36 KB)

## Abbreviations

RNA-Seq: RNA sequencing
BC: Breast cancer
GES: Gene expression signatures
fRMA: Frozen robust multiarray analysis
ER: Estrogen receptor
PgR: Progesterone receptor
HER2: Human epidermal growth factor receptor 2
IHC: Immunohistochemistry
FPKM: Fragments per kilobase per million mapped reads.
