# Supplementary material for: Transfer of clinically relevant gene expression signatures in breast cancer: from Affymetrix microarray to Illumina RNA-Sequencing technology
Source: BMC Genomics. 2014 Nov 21;15(1):1008. doi: 10.1186/1471-2164-15-1008 (PMC4289354; doi:10.1186/1471-2164-15-1008)
Supplement: Supplementary file 2 — Additional file 2: Figure S1: A and B: Box plots showing median level of expression on both Affymetrix microarray and Illumina RNA-Seq platforms for negatively and positively correlated genes. (PDF 9 KB) [file 12864_2014_6829_MOESM2_ESM.pdf]

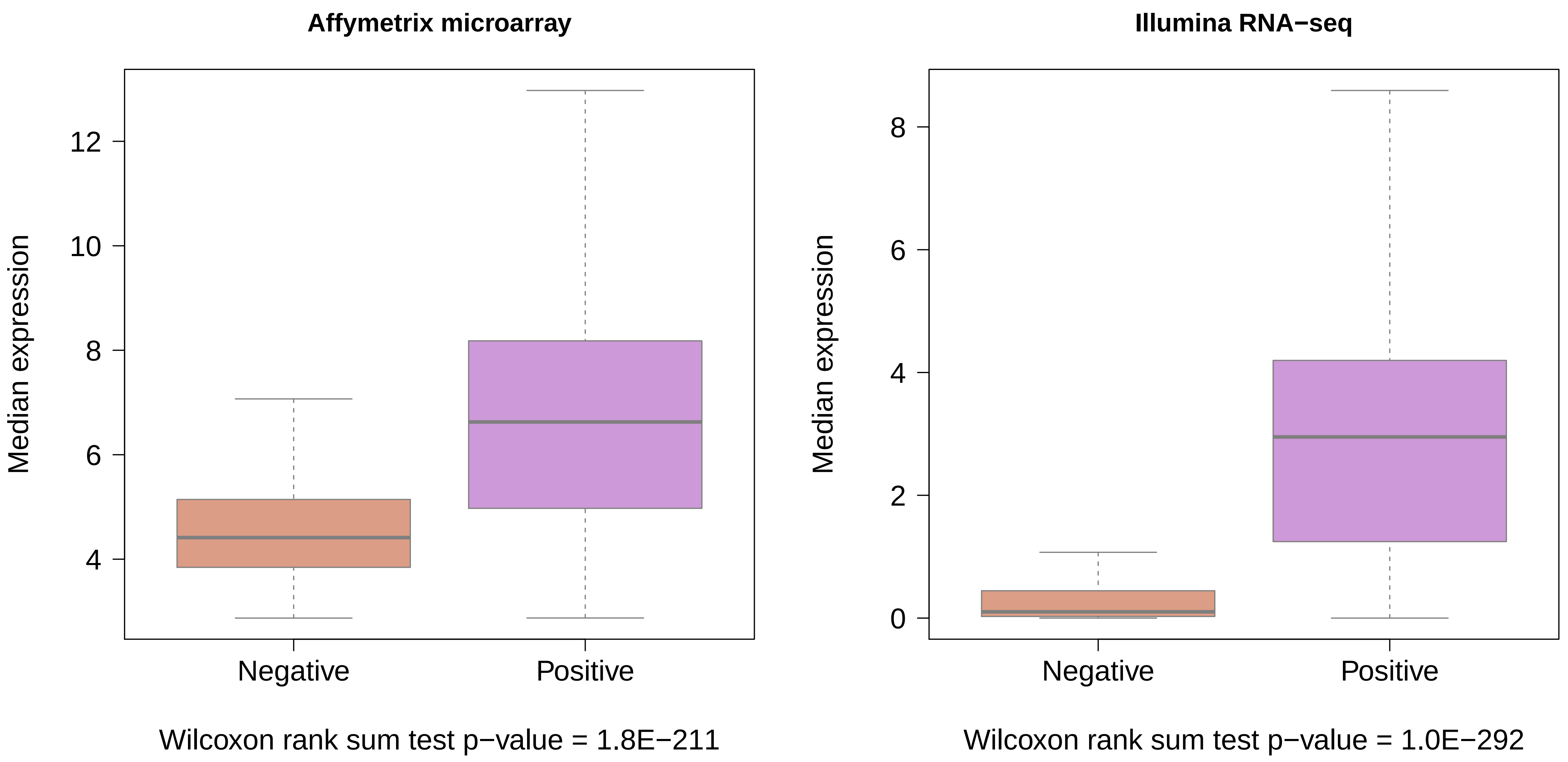

Affymetrix microarray
Median expression
12
10
8
6
4
Negative
Positive
Wilcoxon rank sum test p−value = 1.8E−211
Illumina RNA−seq
Median expression
8
6
4
2
0
Negative
Positive
Wilcoxon rank sum test p−value = 1.0E−292
