# Supplementary material for: Transfer of clinically relevant gene expression signatures in breast cancer: from Affymetrix microarray to Illumina RNA-Sequencing technology
Source: BMC Genomics. 2014 Nov 21;15(1):1008. doi: 10.1186/1471-2164-15-1008 (PMC4289354; doi:10.1186/1471-2164-15-1008)
Supplement: Supplementary file 3 — Additional file 3: Figure S2: Correlation of gene expression levels between Affymetrix microarray and Illumina RNA-Seq platforms with respect to increasing quantiles of gene expression. (PDF 30 KB) [file 12864_2014_6829_MOESM3_ESM.pdf]

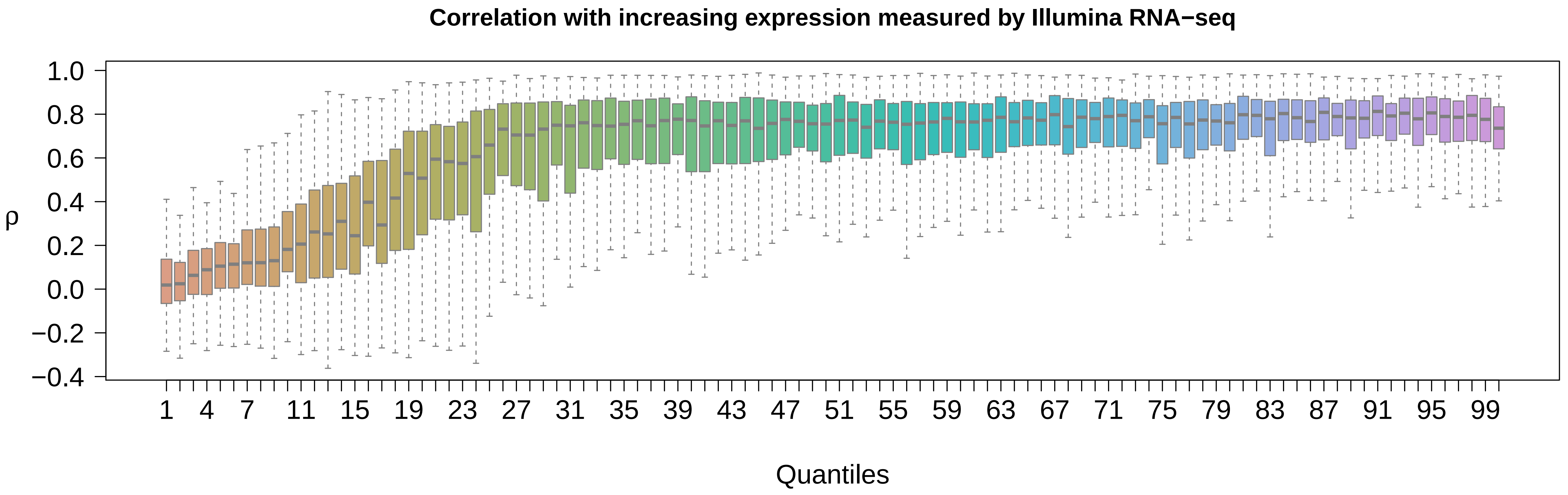
Correlation with increasing expression measured by Illumina RNA−seq
ρ
1.0
0.8
0.6
0.4
0.2
0.0
−0.2
−0.4
1 4 7 11 15 19 23 27 31 35 39 43 47 51 55 59 63 67 71 75 79 83 87 91 95 99
Quantiles

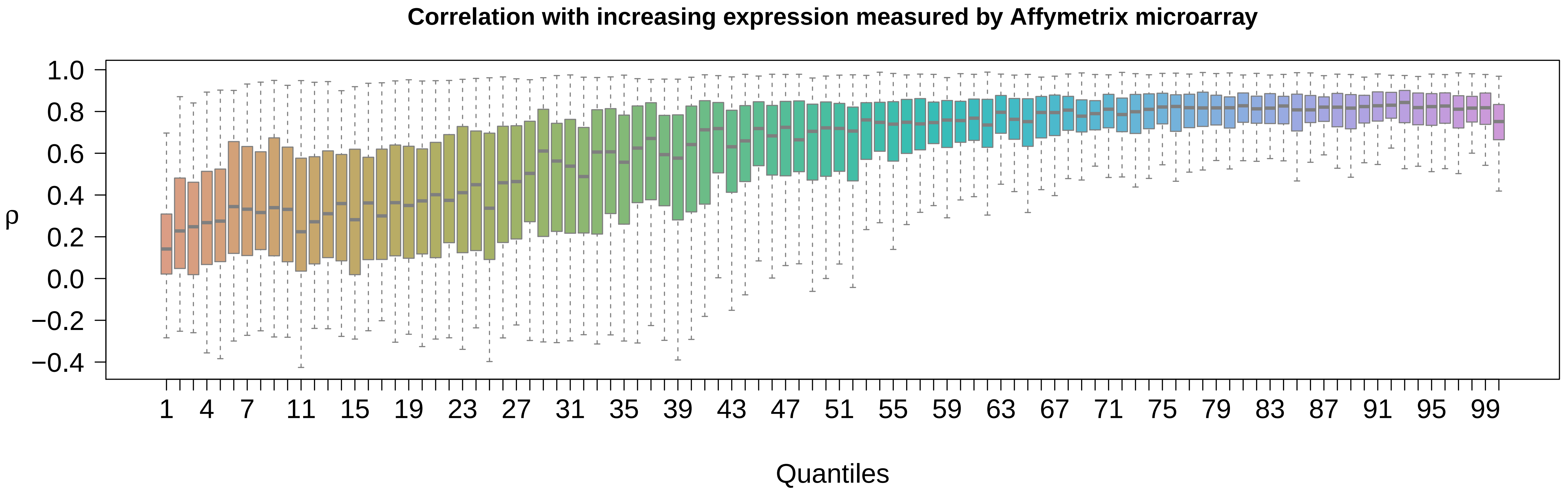
Correlation with increasing expression measured by Affymetrix microarray
ρ
1.0
0.8
0.6
0.4
0.2
0.0
−0.2
−0.4
1 4 7 11 15 19 23 27 31 35 39 43 47 51 55 59 63 67 71 75 79 83 87 91 95 99
Quantiles
