# Supplementary material for: Transfer of clinically relevant gene expression signatures in breast cancer: from Affymetrix microarray to Illumina RNA-Sequencing technology
Source: BMC Genomics. 2014 Nov 21;15(1):1008. doi: 10.1186/1471-2164-15-1008 (PMC4289354; doi:10.1186/1471-2164-15-1008)
Supplement: Supplementary file 4 — Additional file 4: Figure S3: Scatterplots reporting the point-to-point comparison of gene expression profiles measured by Affymetrix microarray and Illumina RNA-Seq platforms for each individual patient considered in the current study. Spearman correlation coefficient and p-value are provided below the plots. The solid line represents a linear regression of microarray values on the RNA-seq data while the dotted line has the equation y = x. (PDF 757 KB) [file 12864_2014_6829_MOESM4_ESM.pdf]

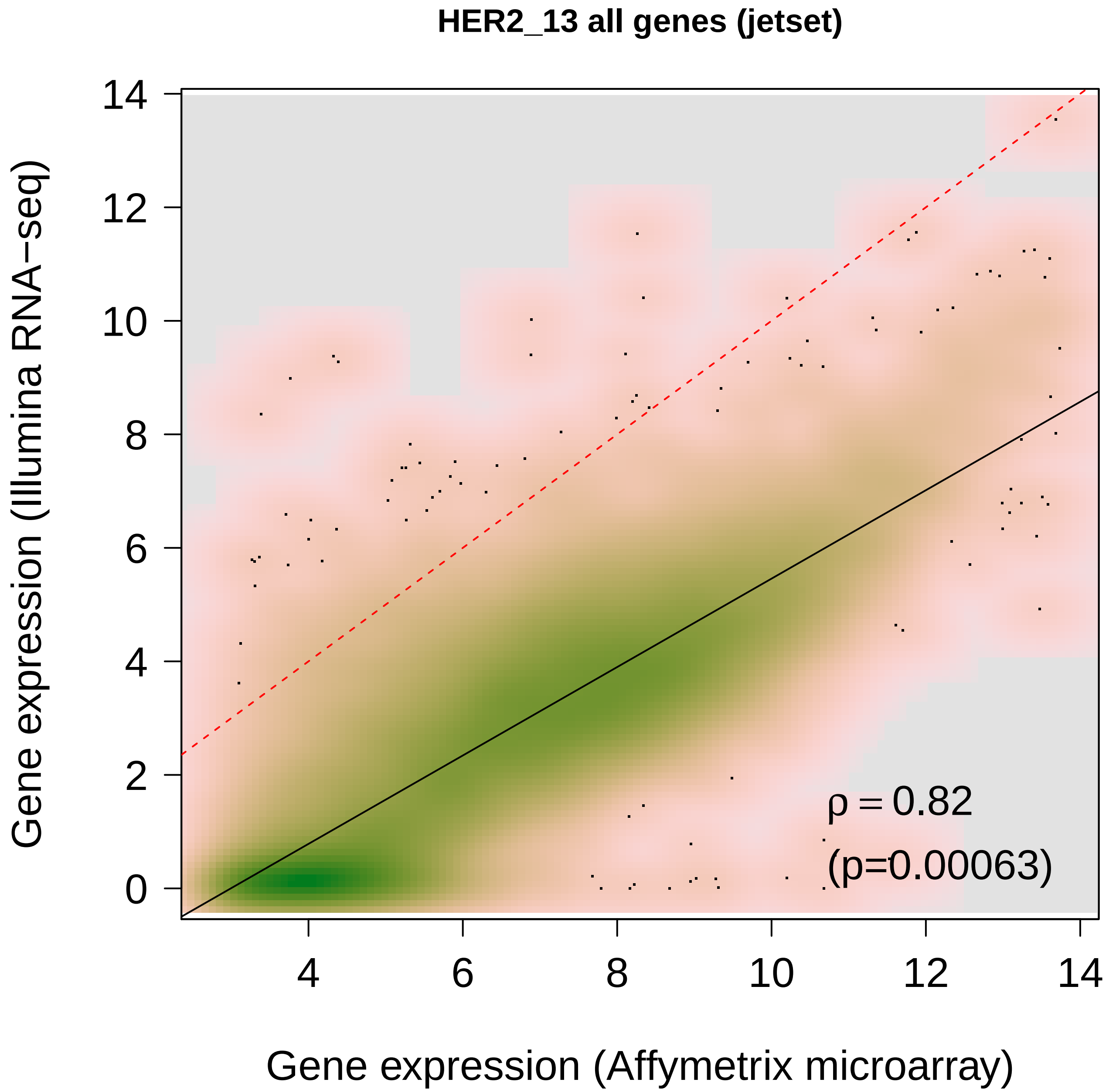

HER2_13 all genes (jetset)
Gene expression (Illumina RNA−seq)
Gene expression (Affymetrix microarray)
ρ = 0.82
(p=0.00063)
0
2
4
6
8
10
12
14

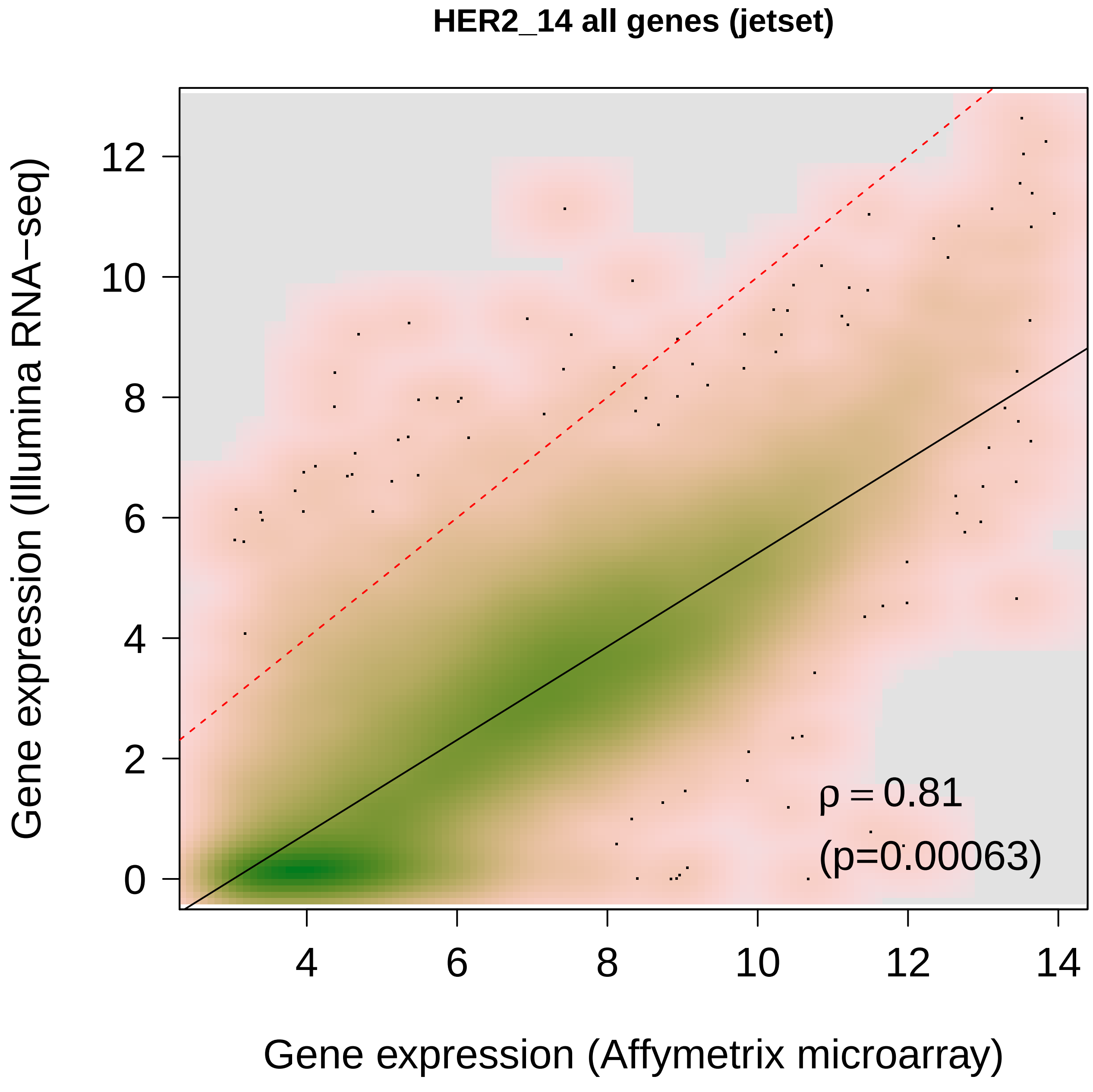

HER2_14 all genes (jetset)
Gene expression (Illumina RNA−seq)
Gene expression (Affymetrix microarray)
0
2
4
6
8
10
12
4
6
8
10
12
14
ρ = 0.81
(p=0.00063)

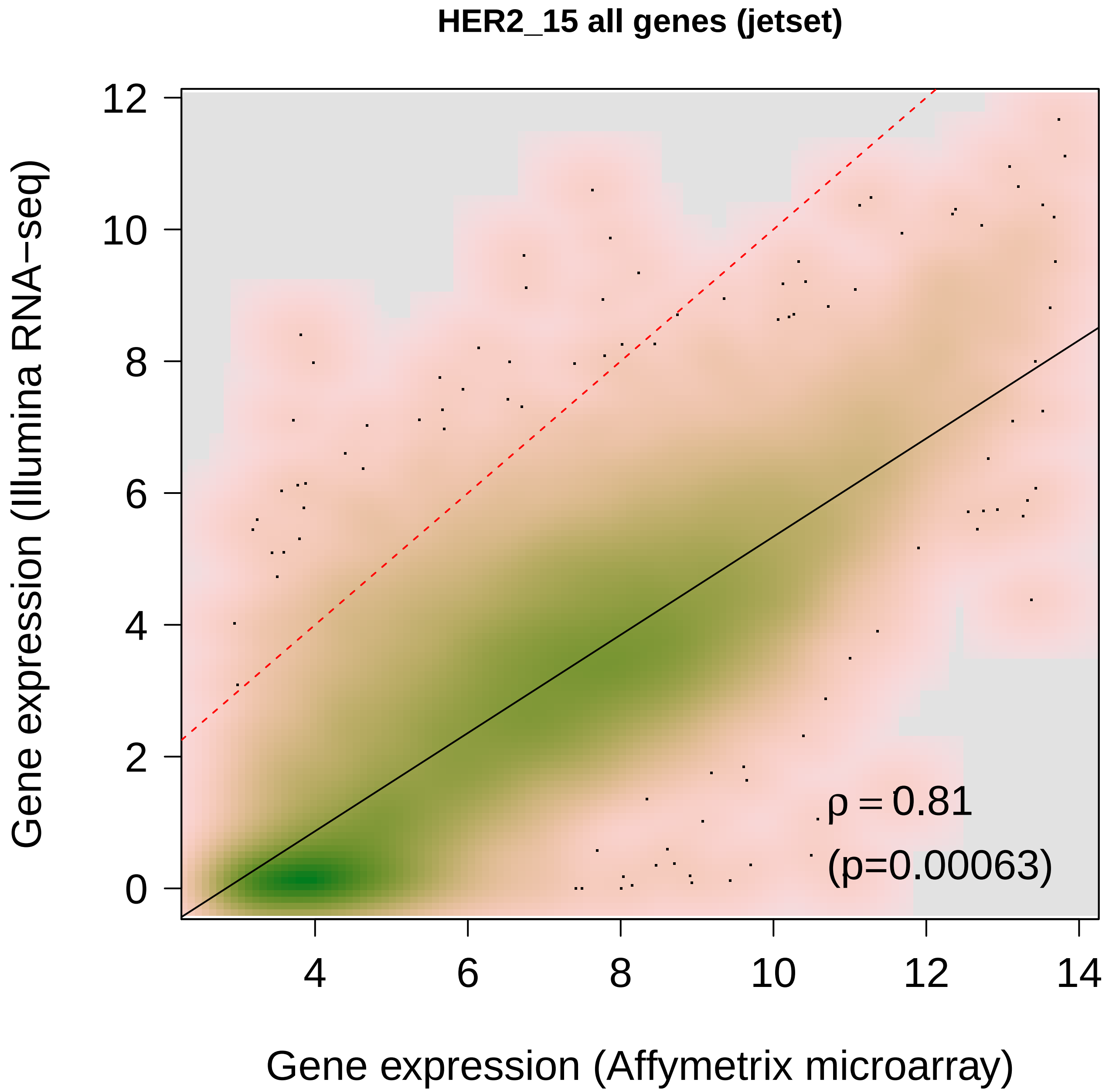
HER2_15 all genes (jetset)
Gene expression (Illumina RNA−seq)
0
2
4
6
8
10
12
4
6
8
10
12
14
ρ = 0.81
(p=0.00063)
Gene expression (Affymetrix microarray)

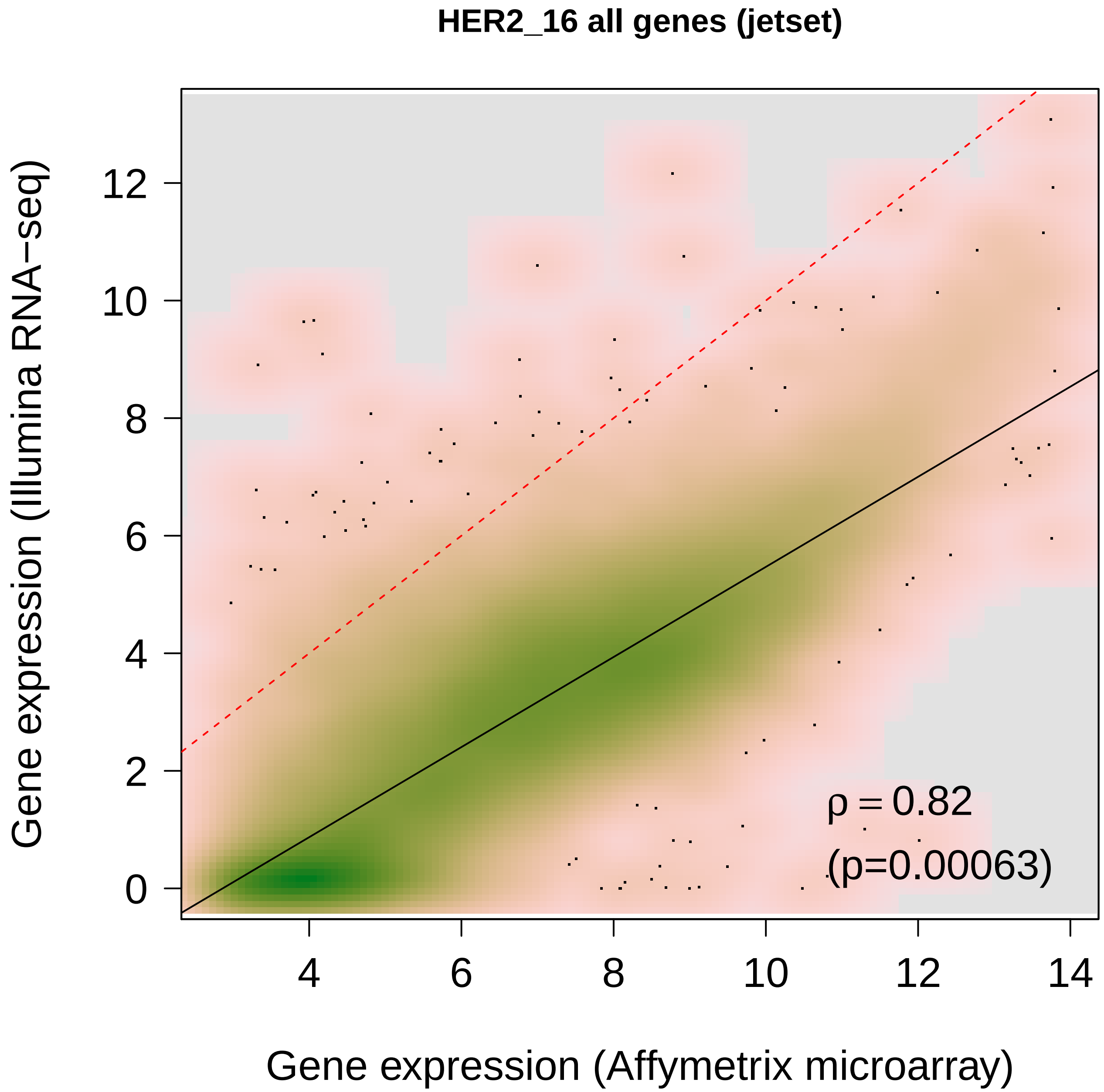

HER2_16 all genes (jetset)
Gene expression (Illumina RNA−seq)
Gene expression (Affymetrix microarray)
0
2
4
6
8
10
12
14
ρ = 0.82
(p=0.00063)

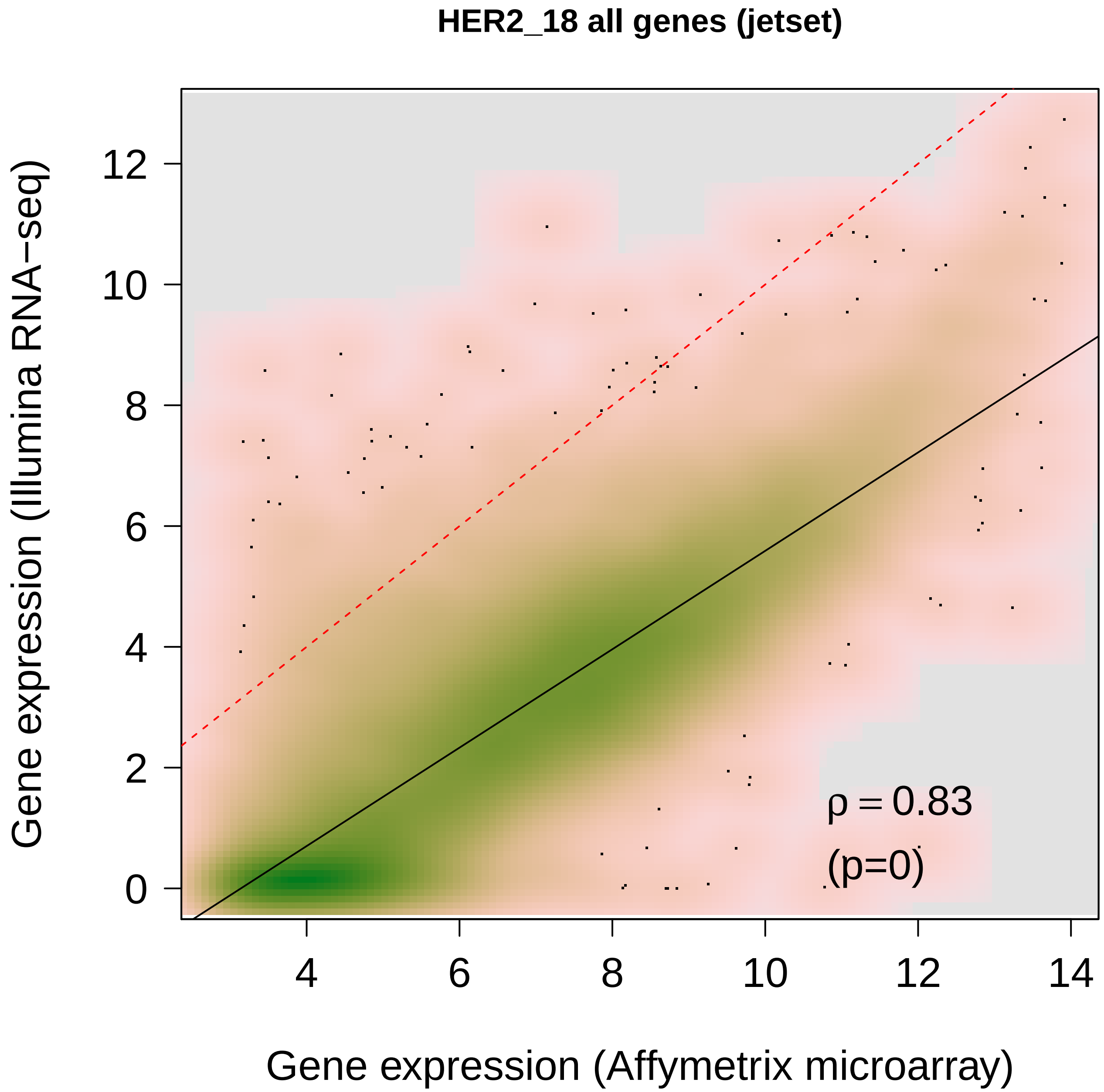

HER2_18 all genes (jetset)
Gene expression (Illumina RNA−seq)
Gene expression (Affymetrix microarray)
ρ = 0.83
(p=0)
0
2
4
6
8
10
12
4
6
8
10
12
14

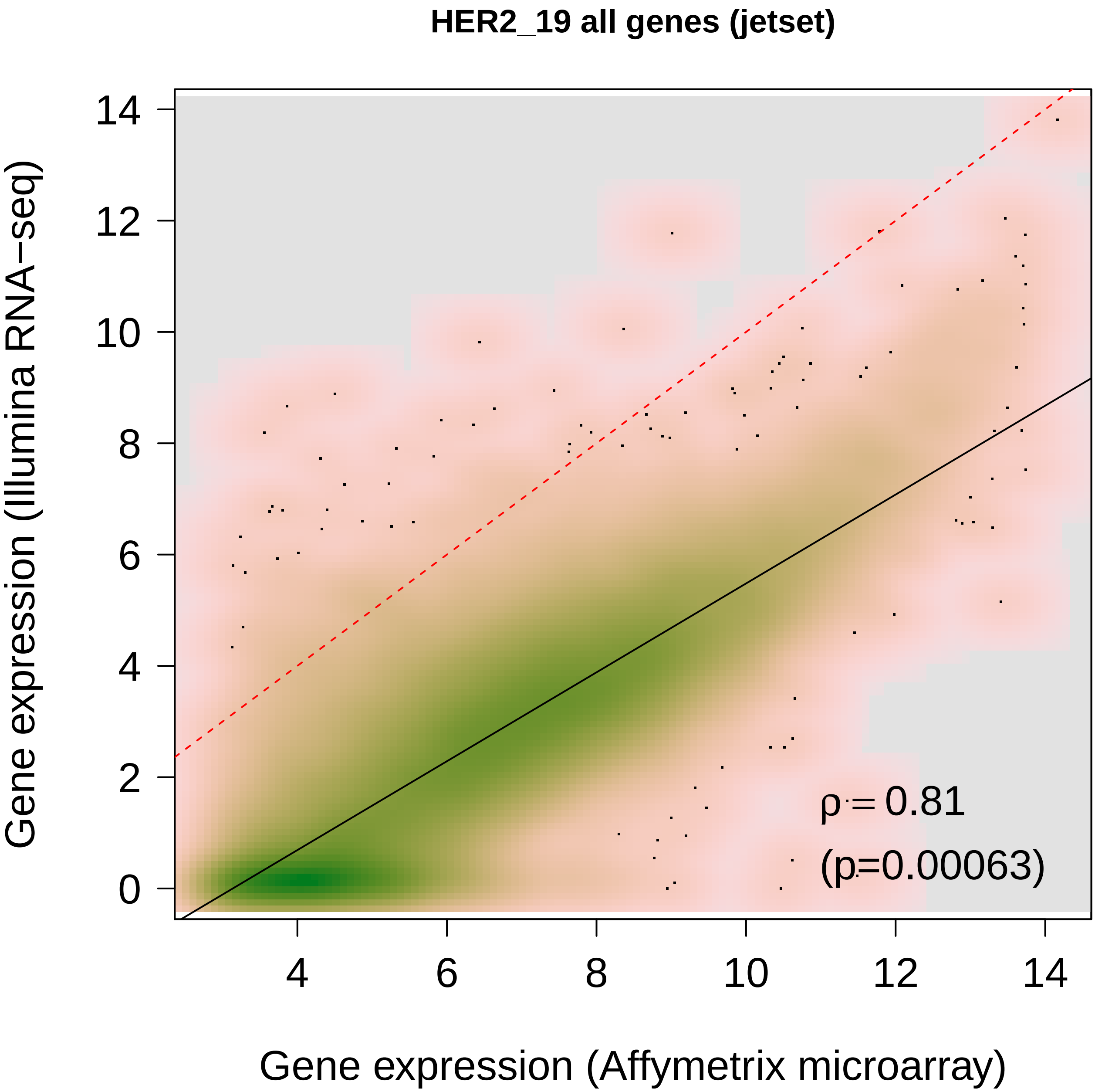
HER2_19 all genes (jetset)
Gene expression (Illumina RNA−seq)
Gene expression (Affymetrix microarray)
0
2
4
6
8
10
12
14
4
6
8
10
12
14
ρ = 0.81
(p=0.00063)

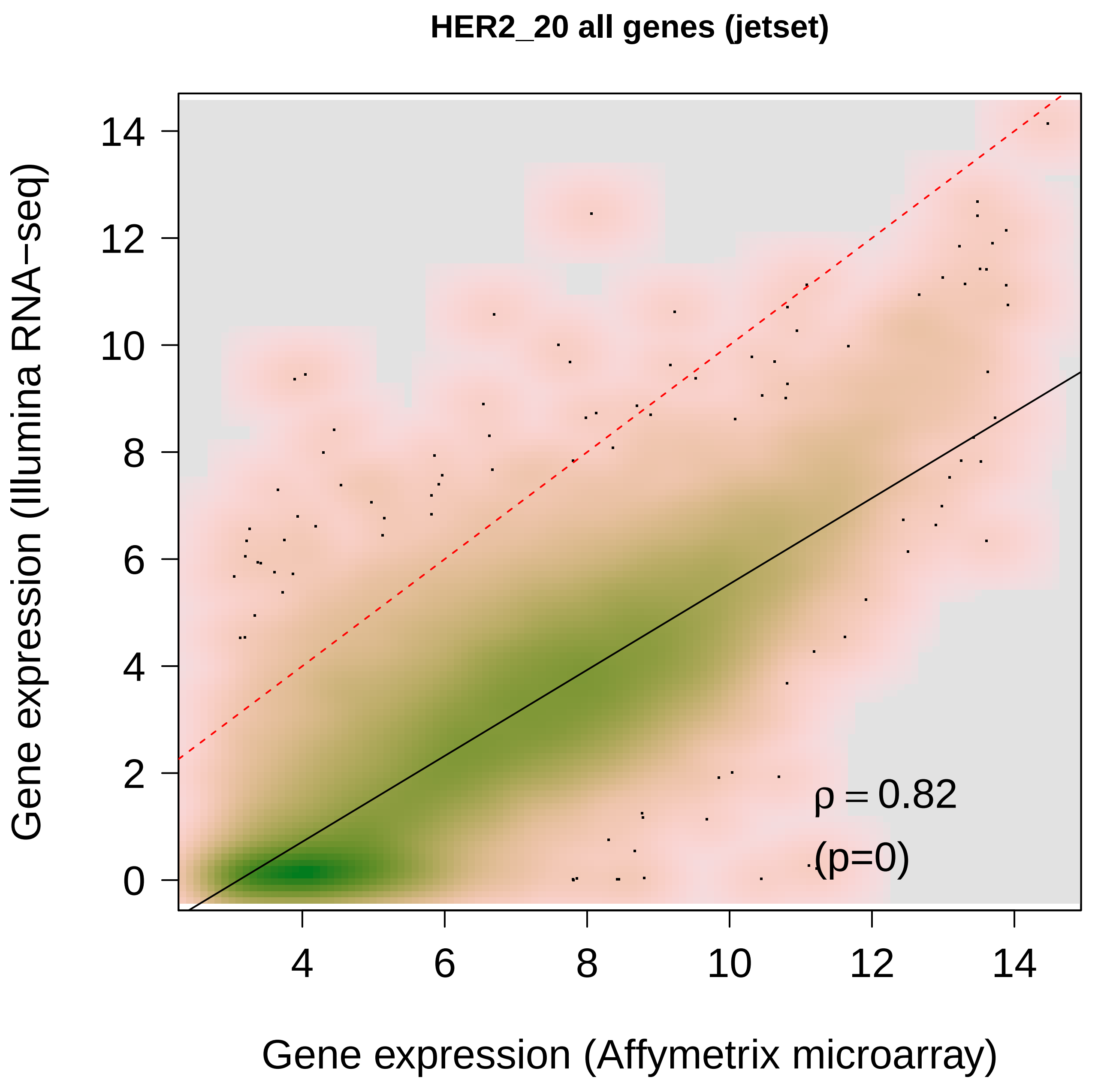
HER2_20 all genes (jetset)
Gene expression (Illumina RNA−seq)
Gene expression (Affymetrix microarray)
ρ = 0.82
(p=0)
0
2
4
6
8
10
12
14
4
6
8
10
12
14

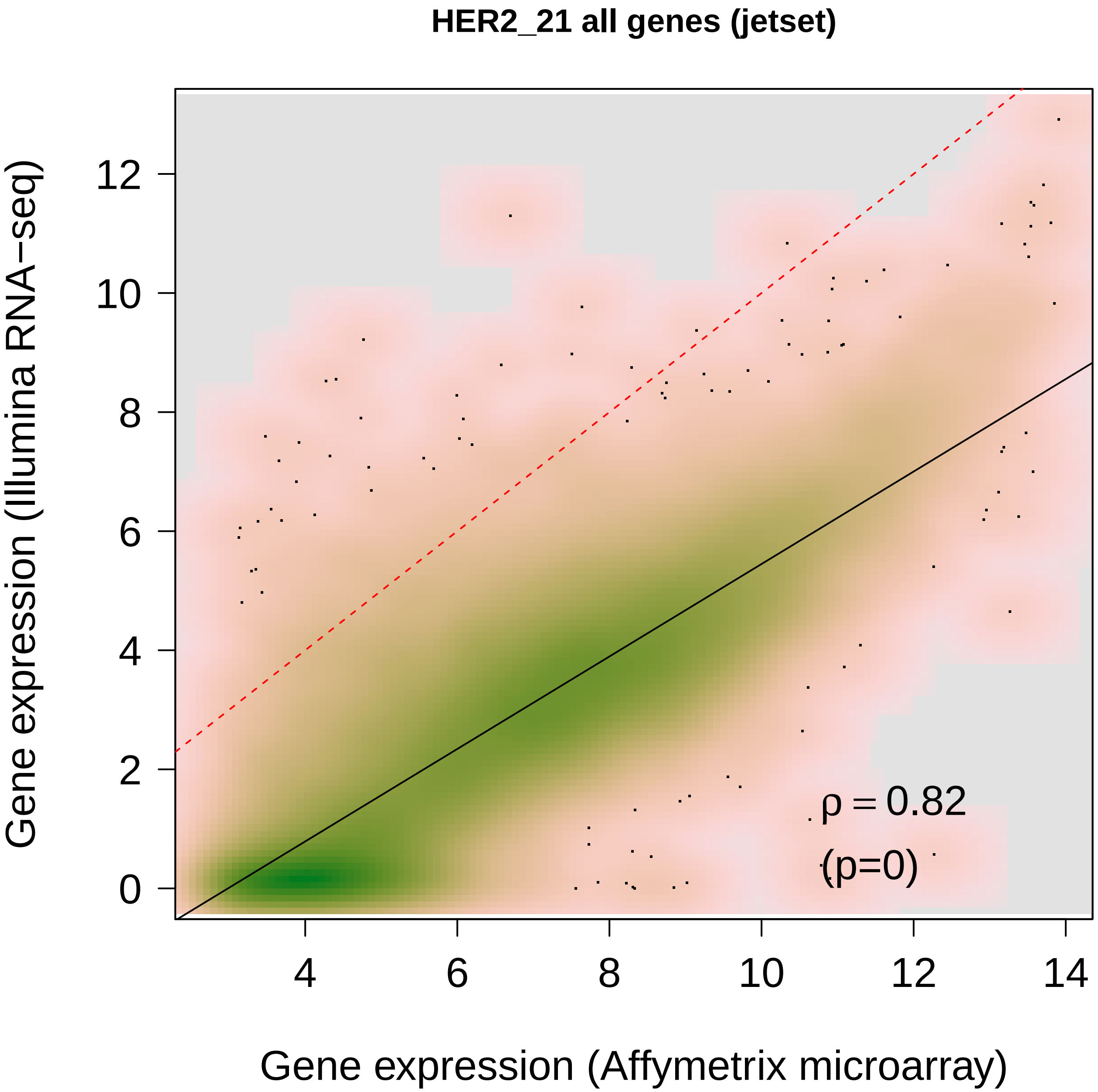

HER2_21 all genes (jetset)
Gene expression (Illumina RNA−seq)
Gene expression (Affymetrix microarray)
0
2
4
6
8
10
12
4
6
8
10
12
14
ρ = 0.82
(p=0)

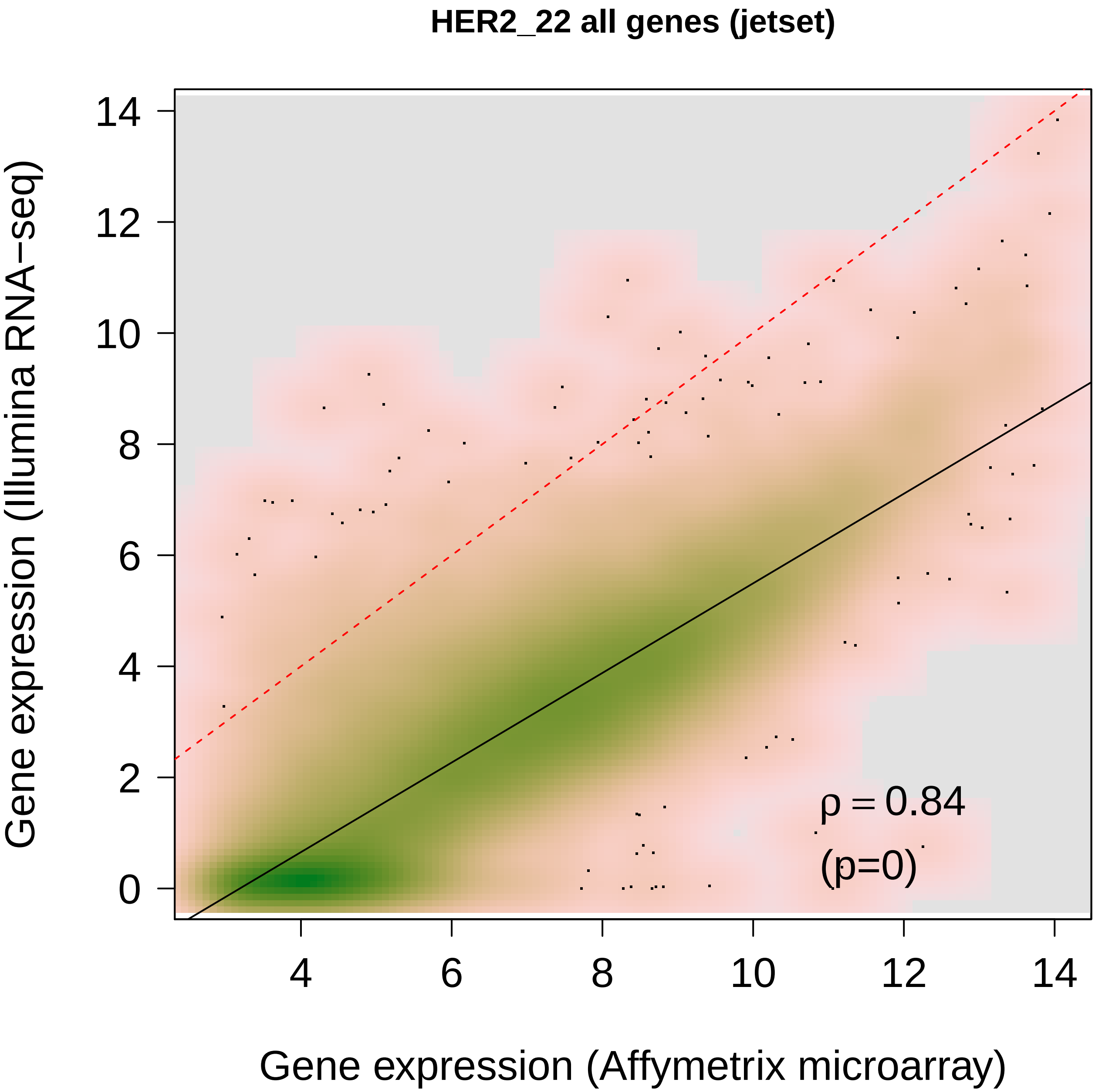

HER2_22 all genes (jetset)
Gene expression (Illumina RNA−seq)
Gene expression (Affymetrix microarray)
ρ = 0.84
(p=0)
0
2
4
6
8
10
12
14
4
6
8
10
12
14

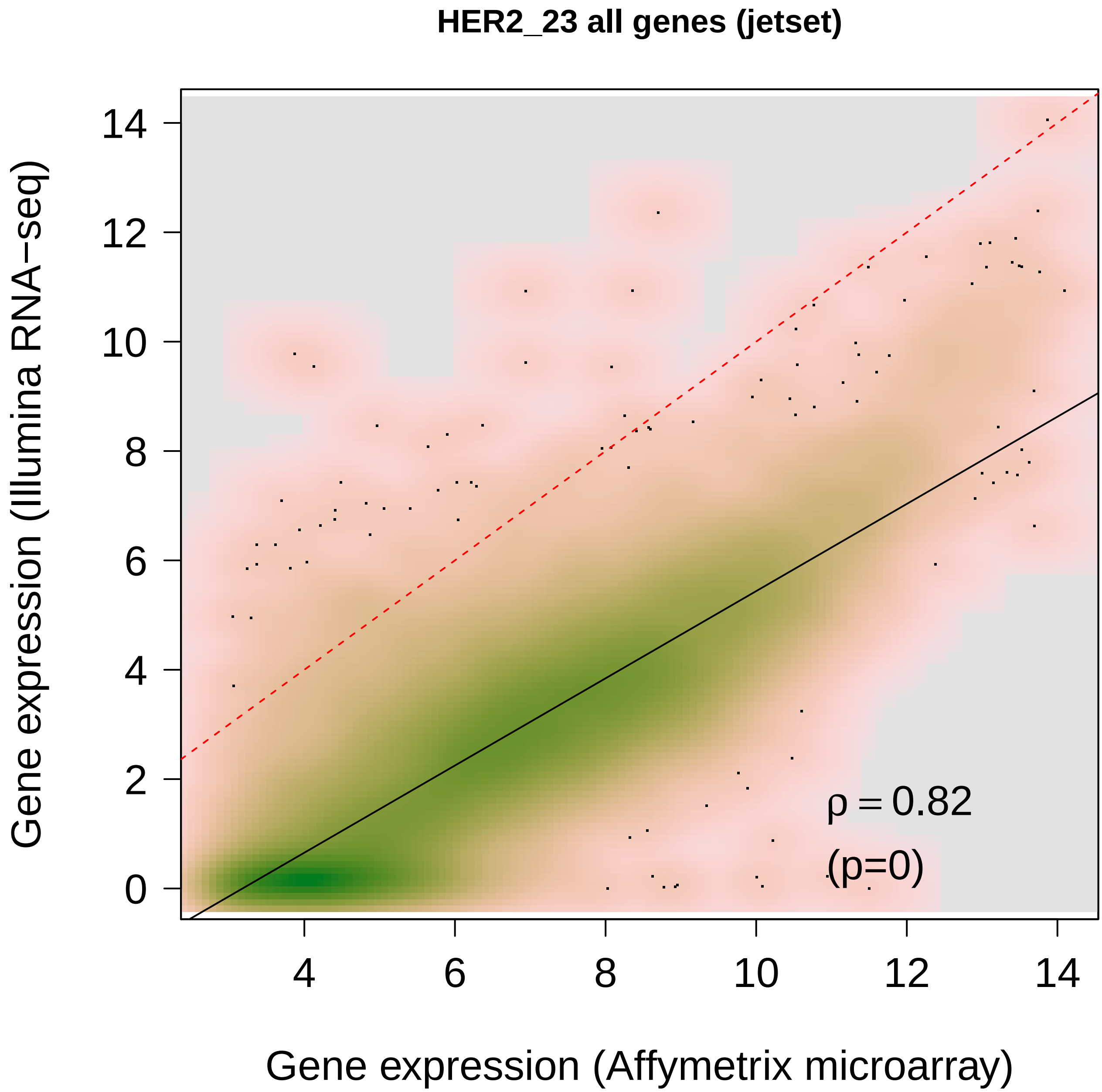

HER2_23 all genes (jetset)
Gene expression (Illumina RNA−seq)
Gene expression (Affymetrix microarray)
0
2
4
6
8
10
12
14
4
6
8
10
12
14
ρ = 0.82
(p=0)

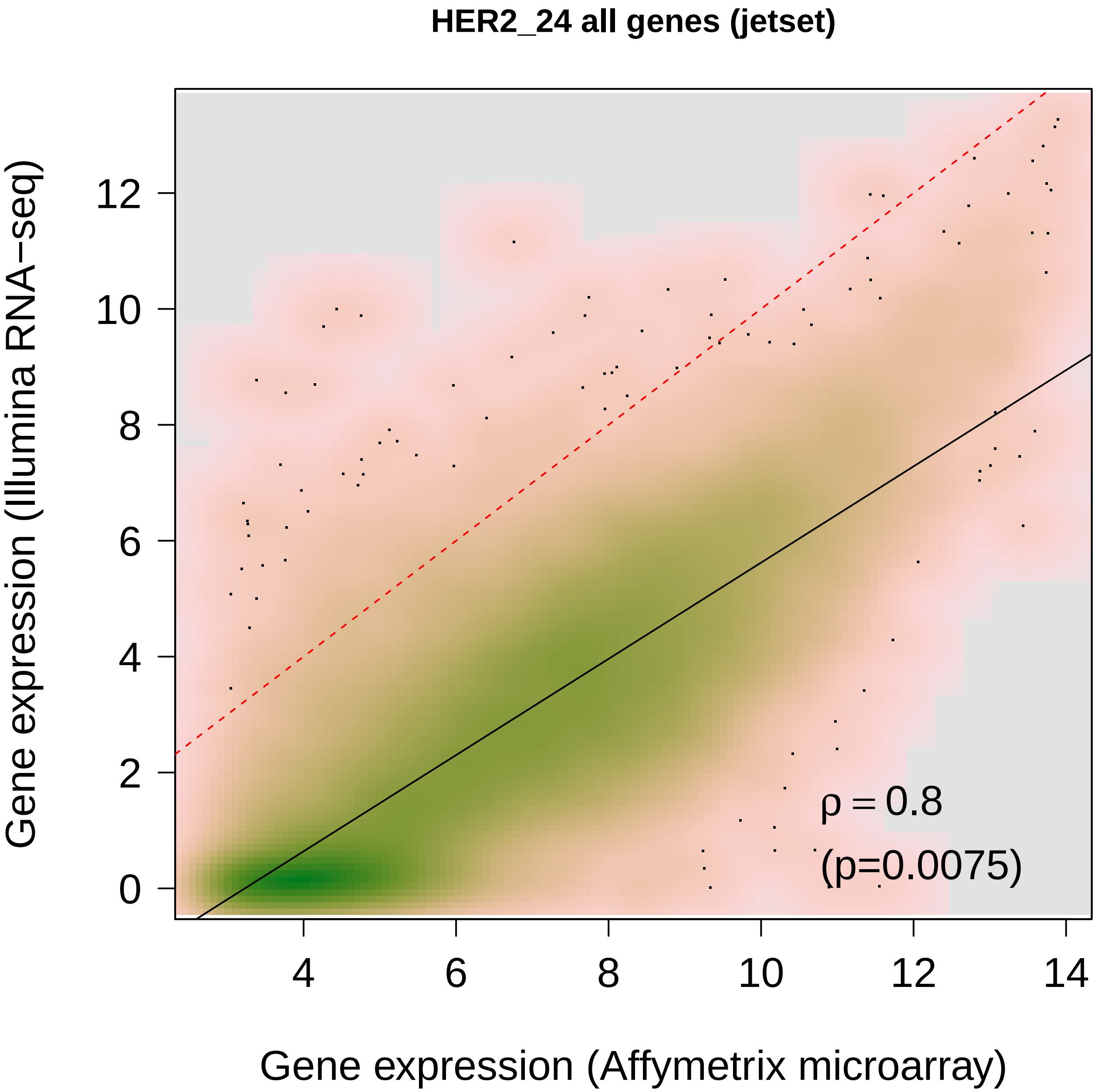

HER2_24 all genes (jetset)
Gene expression (Illumina RNA−seq)
Gene expression (Affymetrix microarray)
0
2
4
6
8
10
12
4
6
8
10
12
14
ρ = 0.8
(p=0.0075)

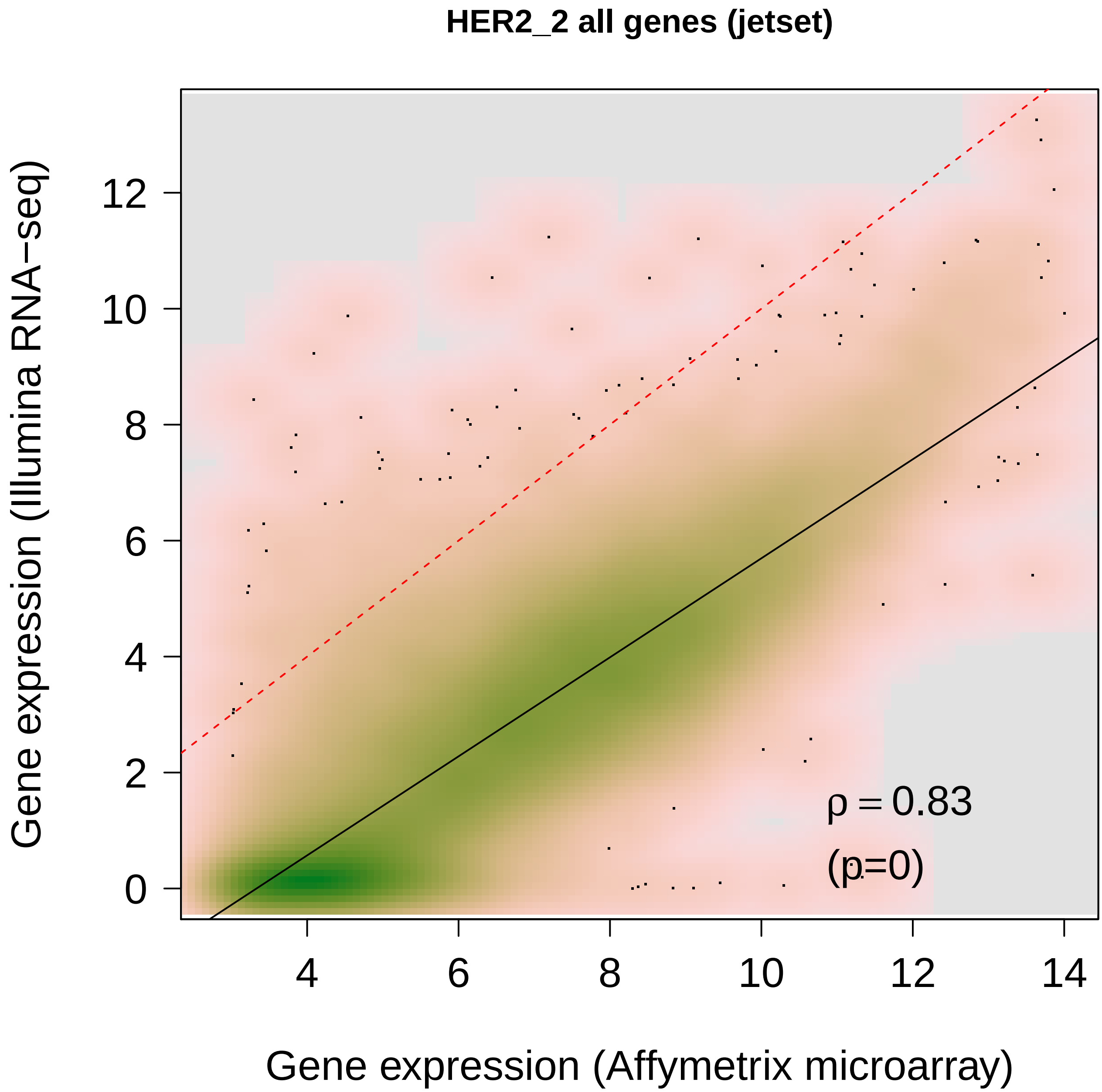

HER2_2 all genes (jetset)
Gene expression (Illumina RNA−seq)
Gene expression (Affymetrix microarray)
0
2
4
6
8
10
12
4
6
8
10
12
14
ρ = 0.83
(p=0)

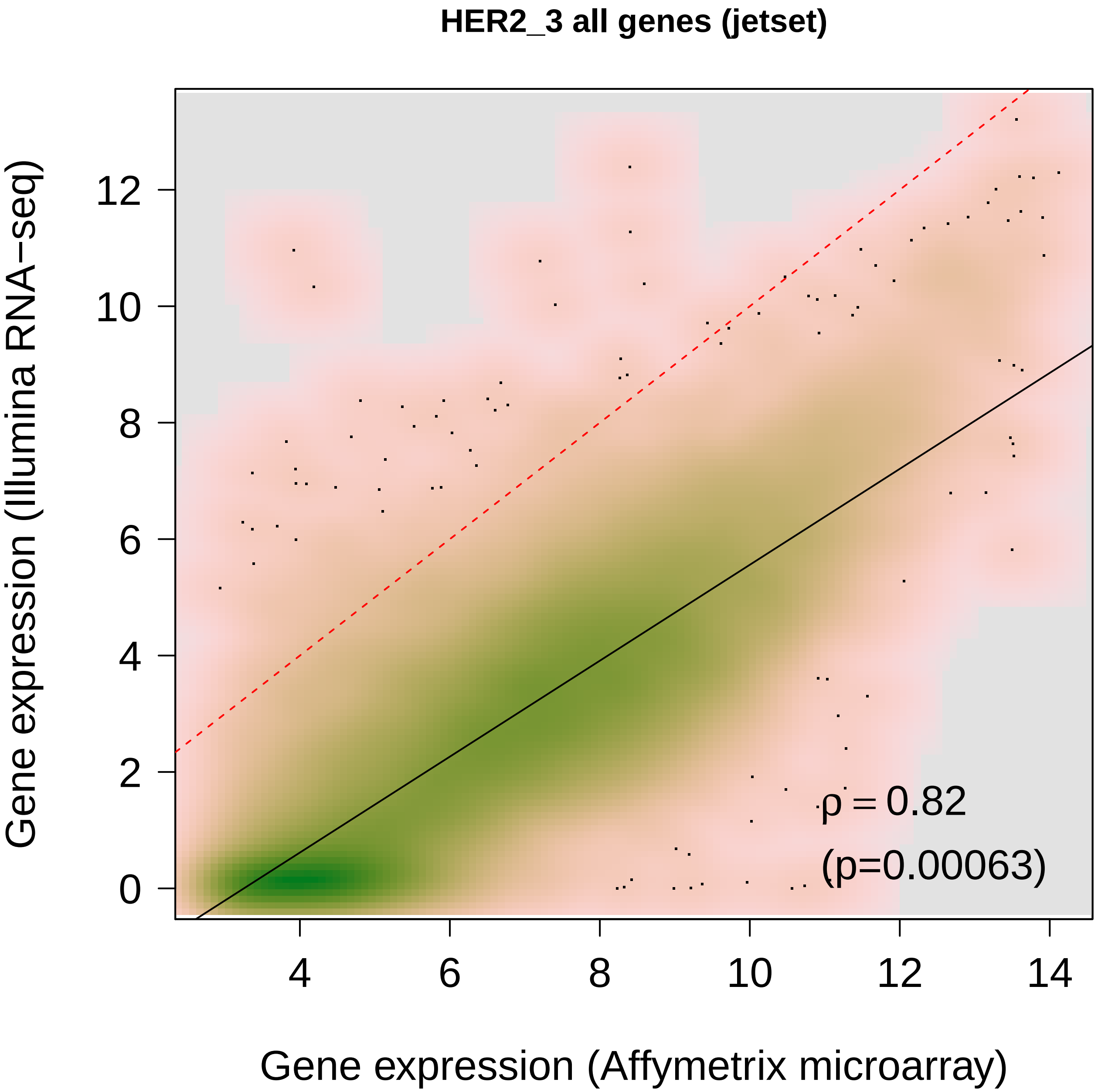

HER2_3 all genes (jetset)
ρ = 0.82
(p=0.00063)
Gene expression (Illumina RNA−seq)
Gene expression (Affymetrix microarray)
0
2
4
6
8
10
12
4
6
8
10
12
14

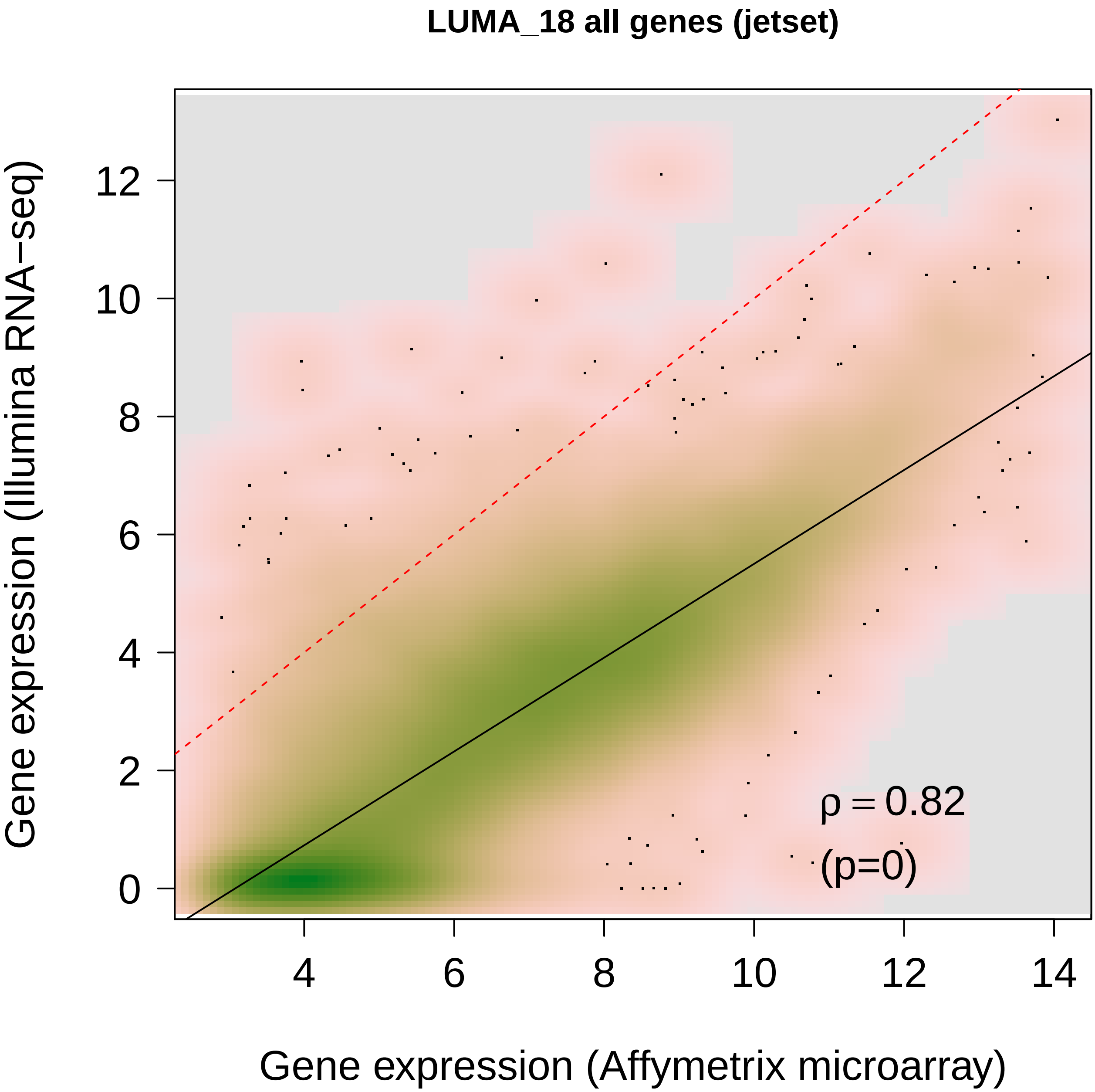

LUMA_18 all genes (jetset)
Gene expression (Illumina RNA−seq)
Gene expression (Affymetrix microarray)
0
2
4
6
8
10
12
4
6
8
10
12
14
ρ = 0.82
(p=0)

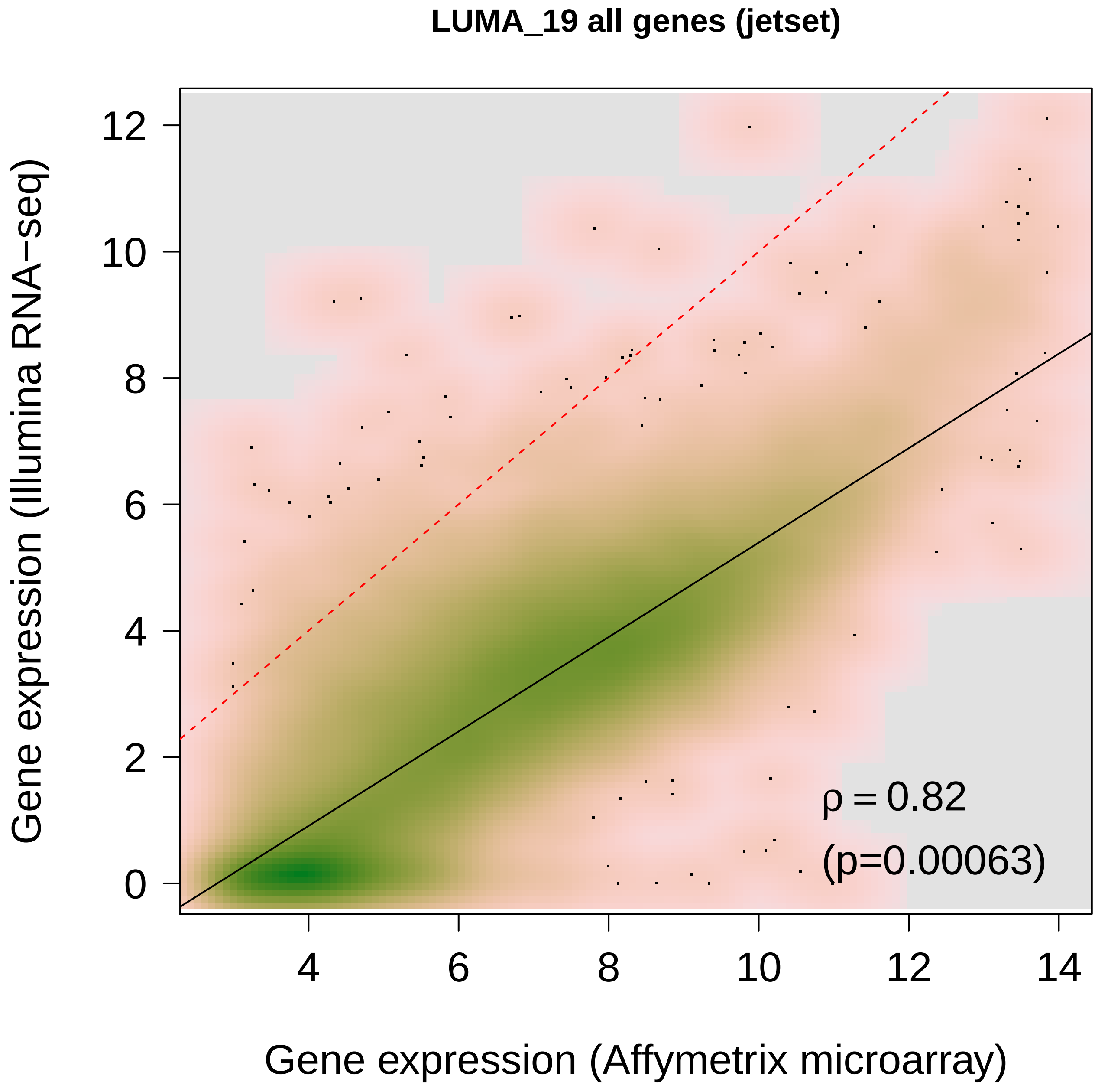
LUMA_19 all genes (jetset)
Gene expression (Illumina RNA−seq)
Gene expression (Affymetrix microarray)
ρ = 0.82
(p=0.00063)
0
2
4
6
8
10
12
4
6
8
10
12
14

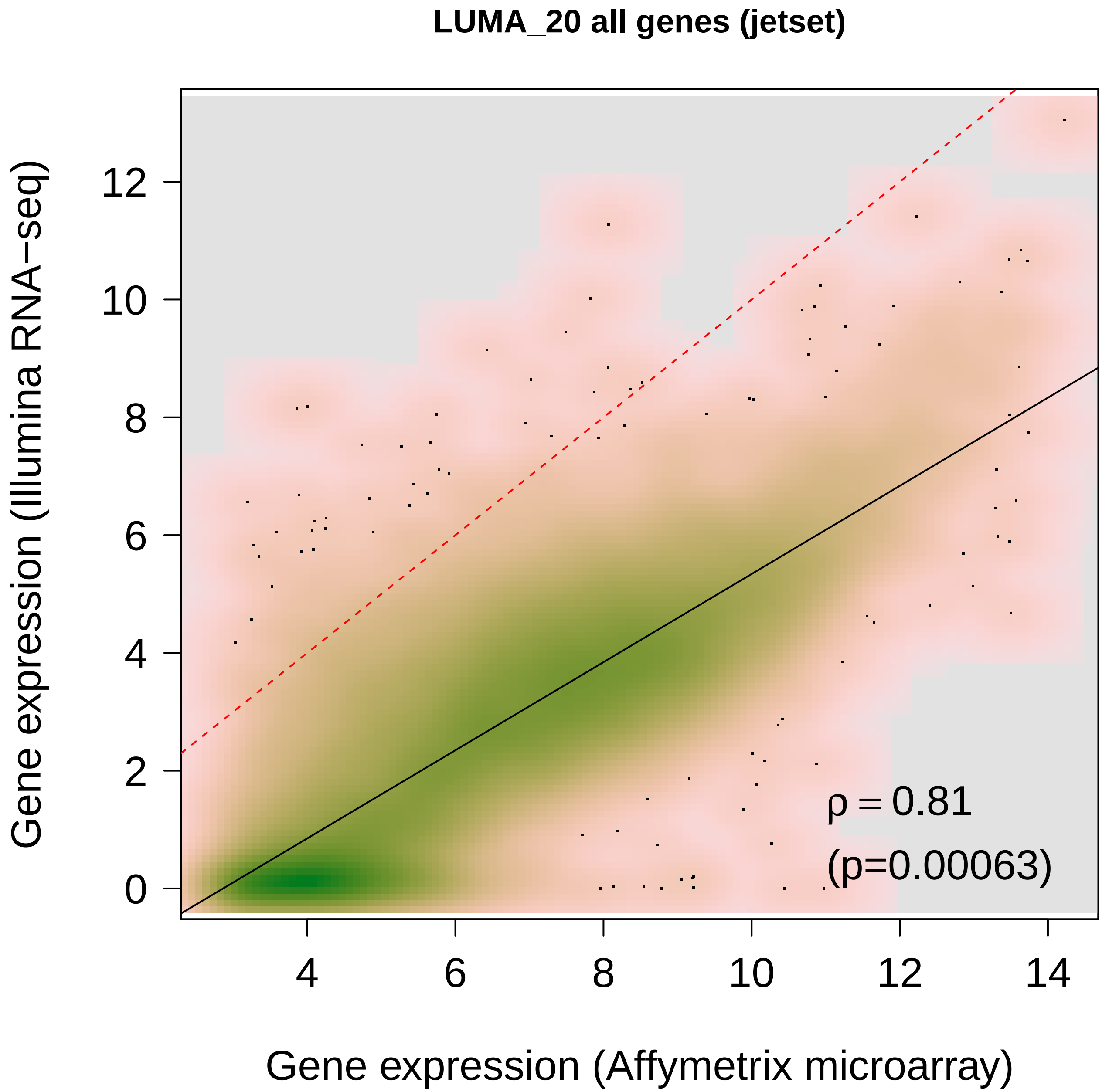
LUMA_20 all genes (jetset)
Gene expression (Illumina RNA−seq)
Gene expression (Affymetrix microarray)
ρ = 0.81
(p=0.00063)
0
2
4
6
8
10
12
4
6
8
10
12
14

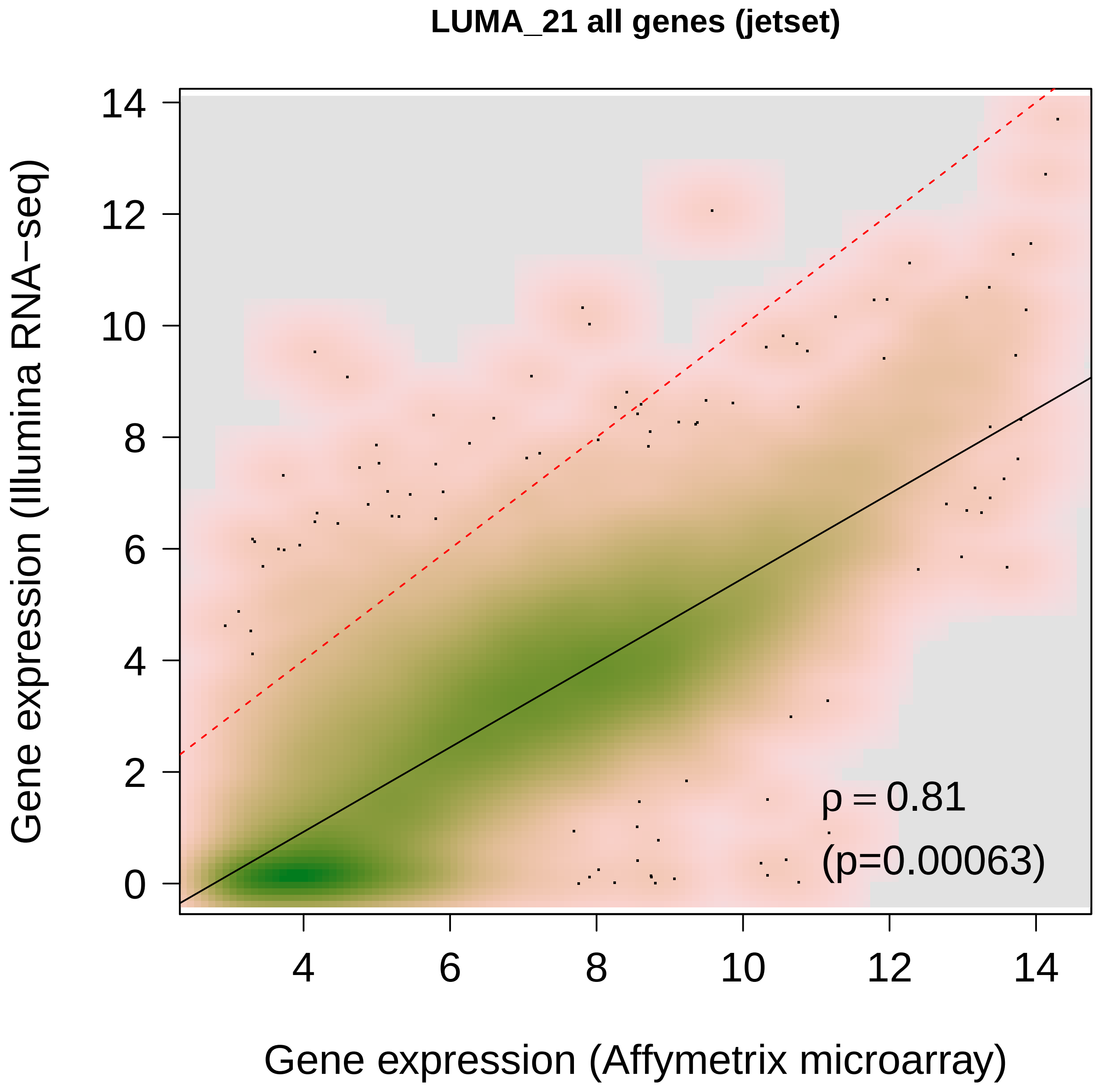
LUMA_21 all genes (jetset)
Gene expression (Illumina RNA−seq)
Gene expression (Affymetrix microarray)
0
2
4
6
8
10
12
14
4
6
8
10
12
14
ρ = 0.81
(p=0.00063)

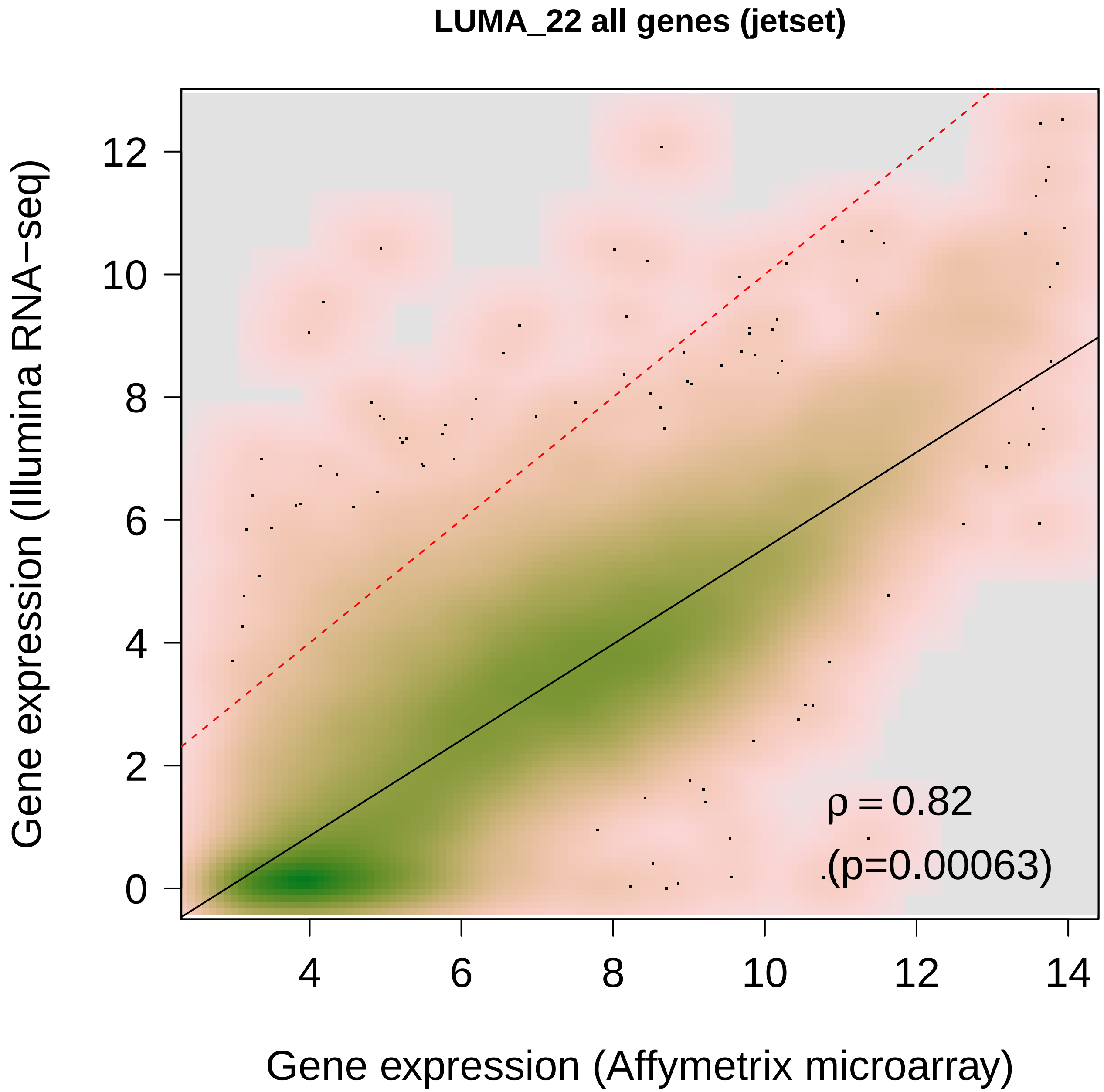

LUMA_22 all genes (jetset)
Gene expression (Illumina RNA−seq)
Gene expression (Affymetrix microarray)
ρ = 0.82
(p=0.00063)
0
2
4
6
8
10
12
4
6
8
10
12
14

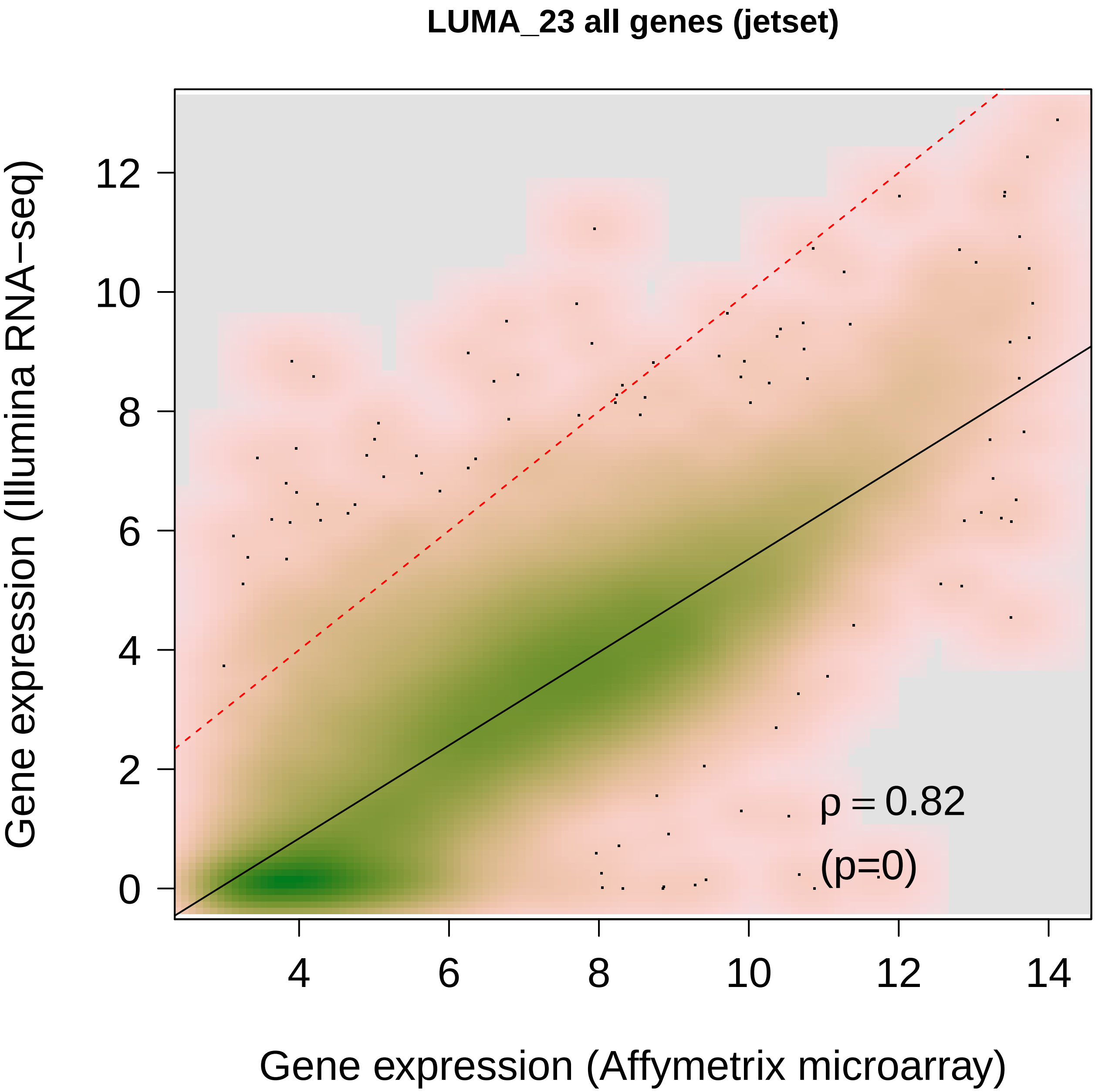

LUMA_23 all genes (jetset)
Gene expression (Illumina RNA−seq)
Gene expression (Affymetrix microarray)
ρ = 0.82
(p=0)
0
2
4
6
8
10
12
4
6
8
10
12
14

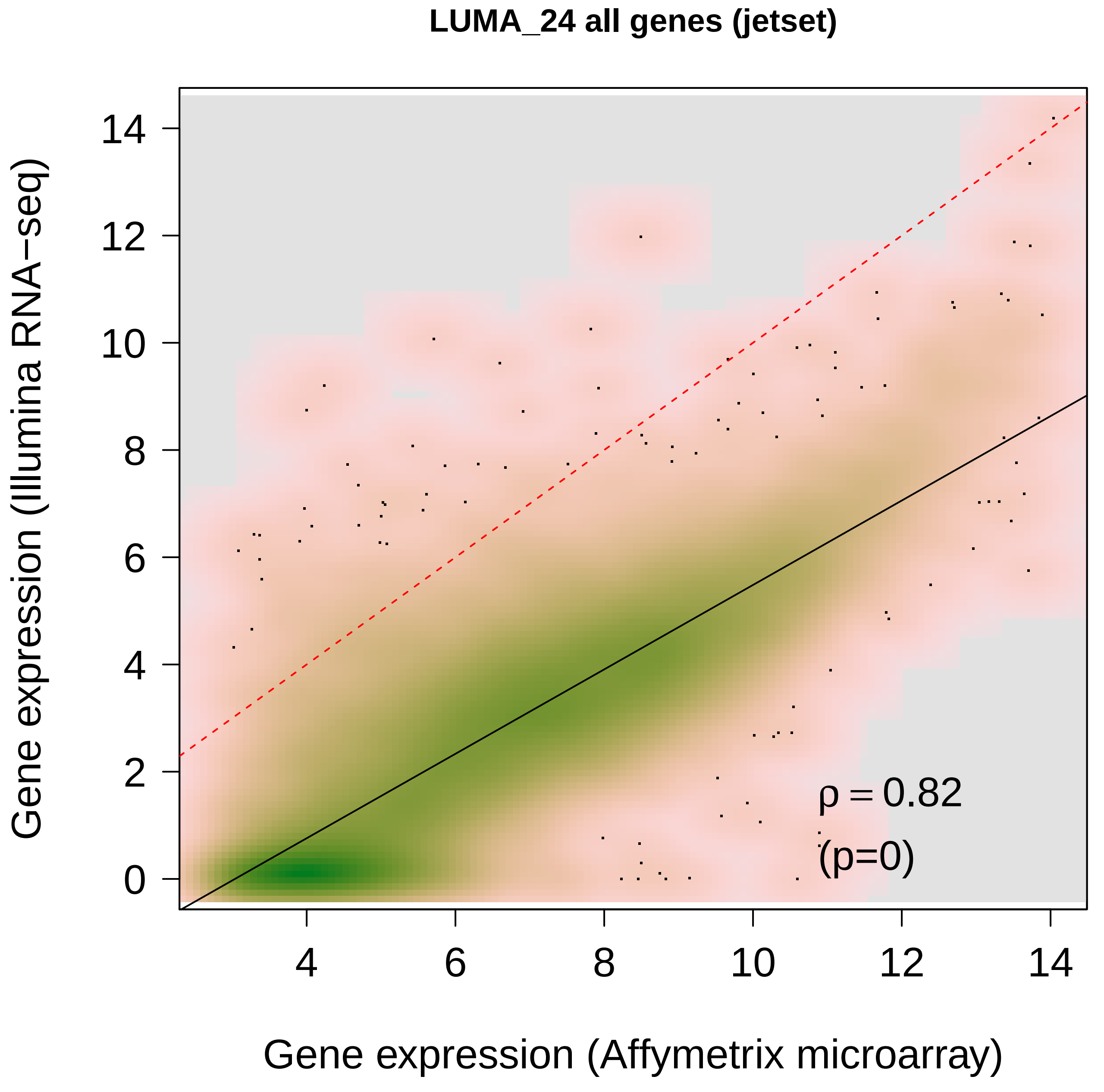

LUMA_24 all genes (jetset)
Gene expression (Illumina RNA−seq)
Gene expression (Affymetrix microarray)
ρ = 0.82
(p=0)
0
2
4
6
8
10
12
14
4
6
8
10
12
14

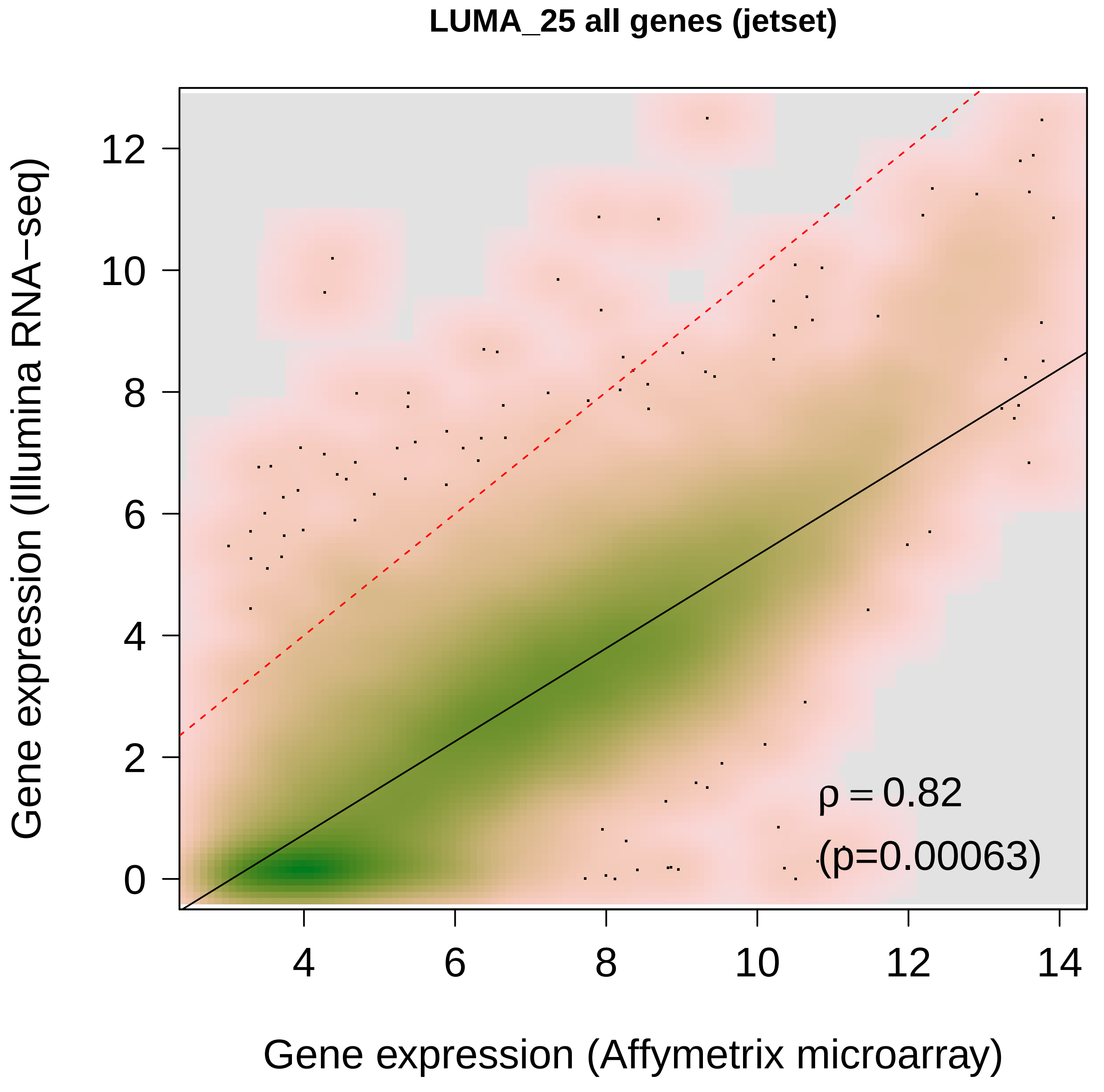
LUMA_25 all genes (jetset)
Gene expression (Illumina RNA−seq)
Gene expression (Affymetrix microarray)
ρ = 0.82
(p=0.00063)
0
2
4
6
8
10
12
4
6
8
10
12
14

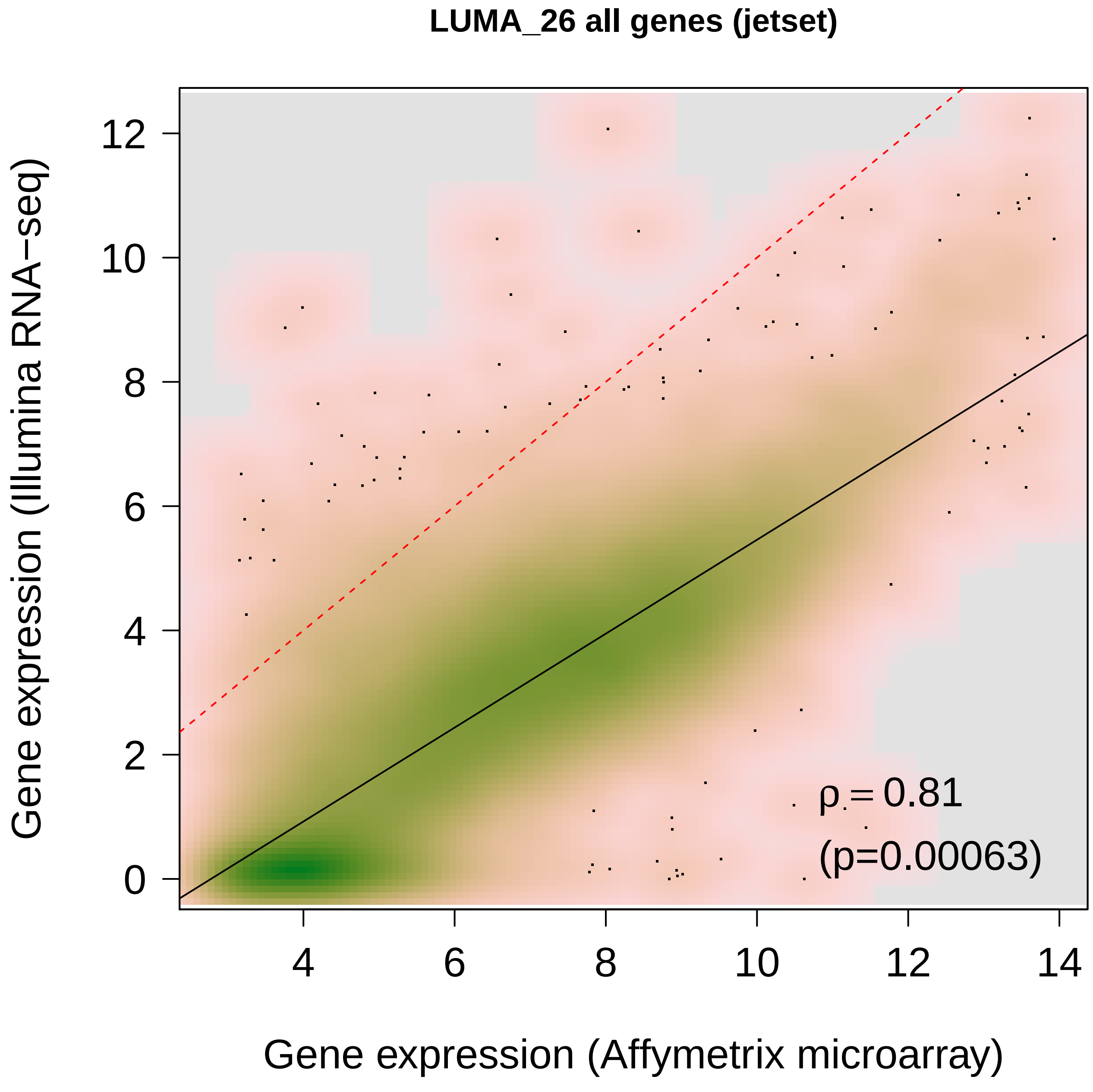

LUMA_26 all genes (jetset)
Gene expression (Illumina RNA−seq)
Gene expression (Affymetrix microarray)
ρ = 0.81
(p=0.00063)
0
2
4
6
8
10
12
4
6
8
10
12
14

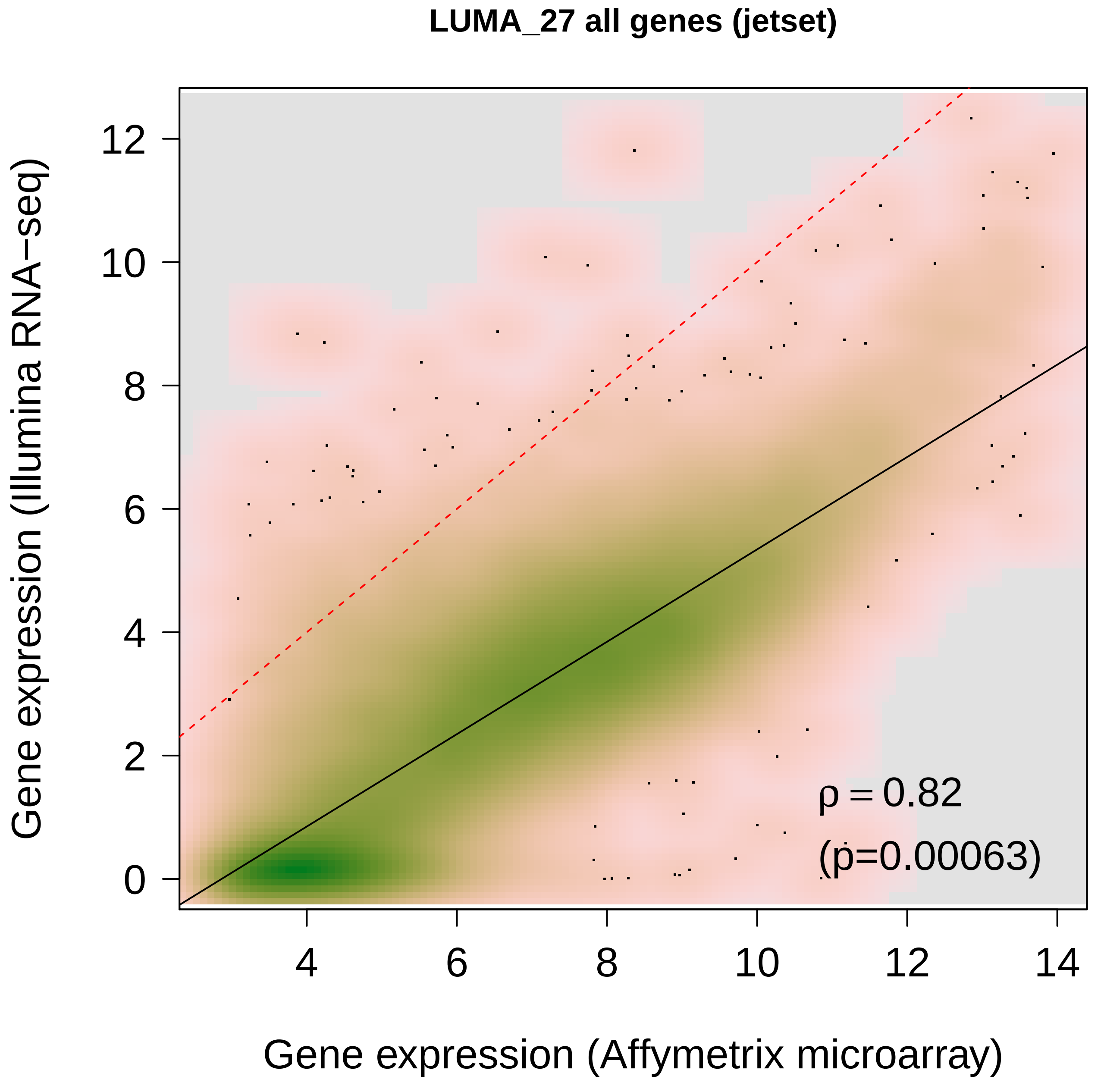

LUMA_27 all genes (jetset)
Gene expression (Illumina RNA−seq)
Gene expression (Affymetrix microarray)
0
2
4
6
8
10
12
4
6
8
10
12
14
ρ = 0.82
(p=0.00063)

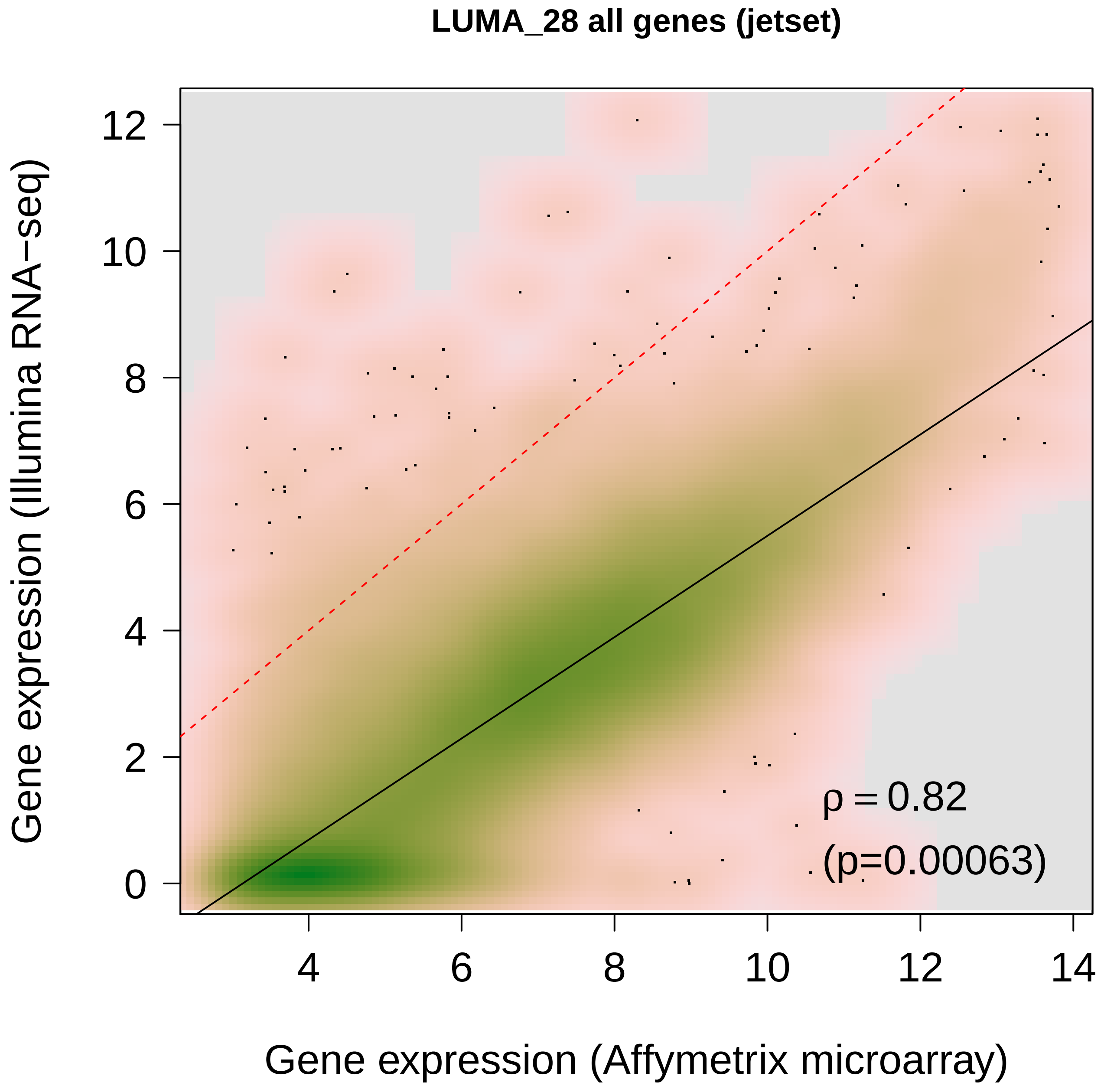
LUMA_28 all genes (jetset)
Gene expression (Illumina RNA−seq)
Gene expression (Affymetrix microarray)
0
2
4
6
8
10
12
4
6
8
10
12
14
ρ = 0.82
(p=0.00063)

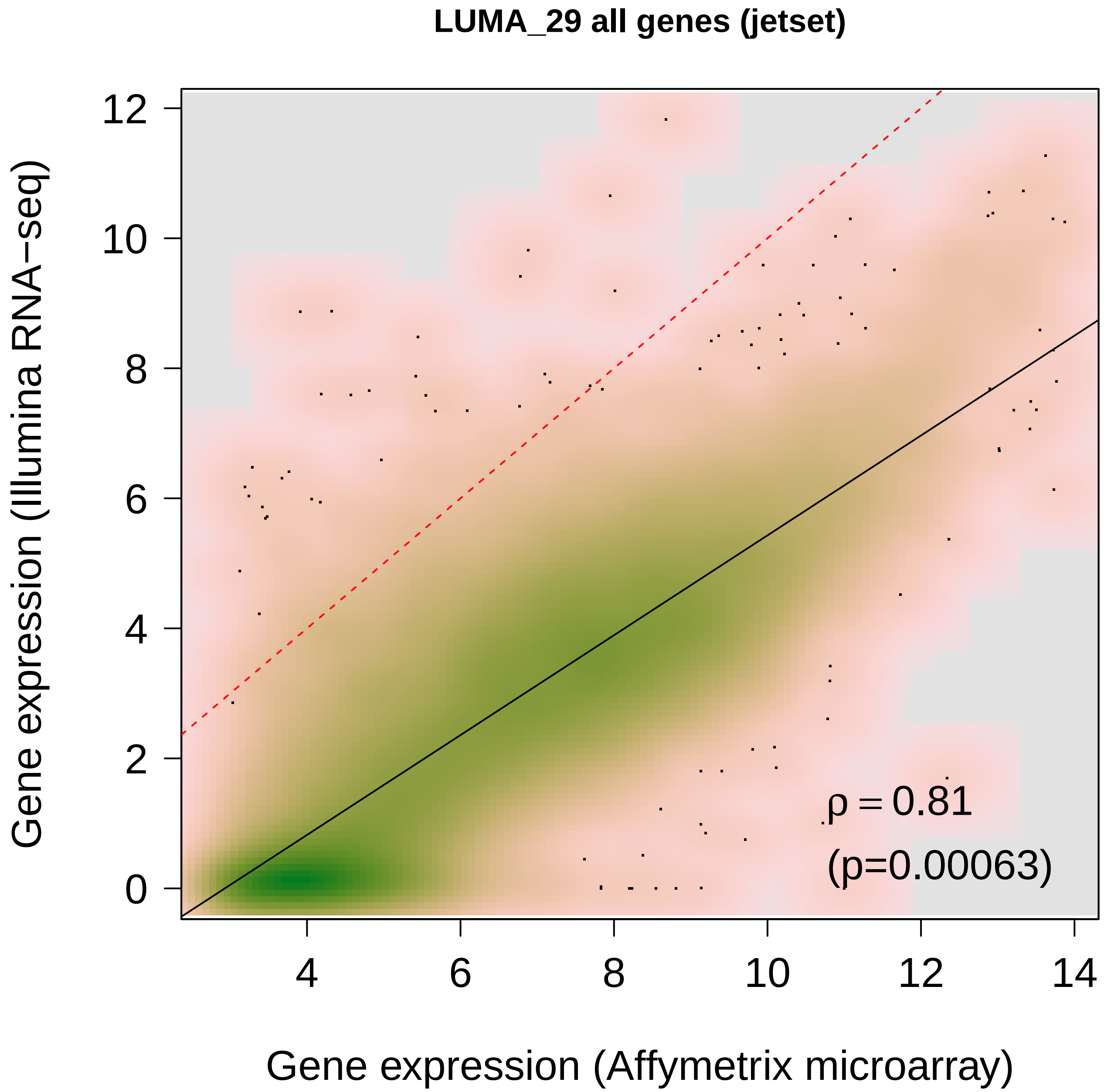
LUMA_29 all genes (jetset)
Gene expression (Illumina RNA−seq)
Gene expression (Affymetrix microarray)
12
10
8
6
4
2
0
4
6
8
10
12
14
ρ = 0.81
(p=0.00063)

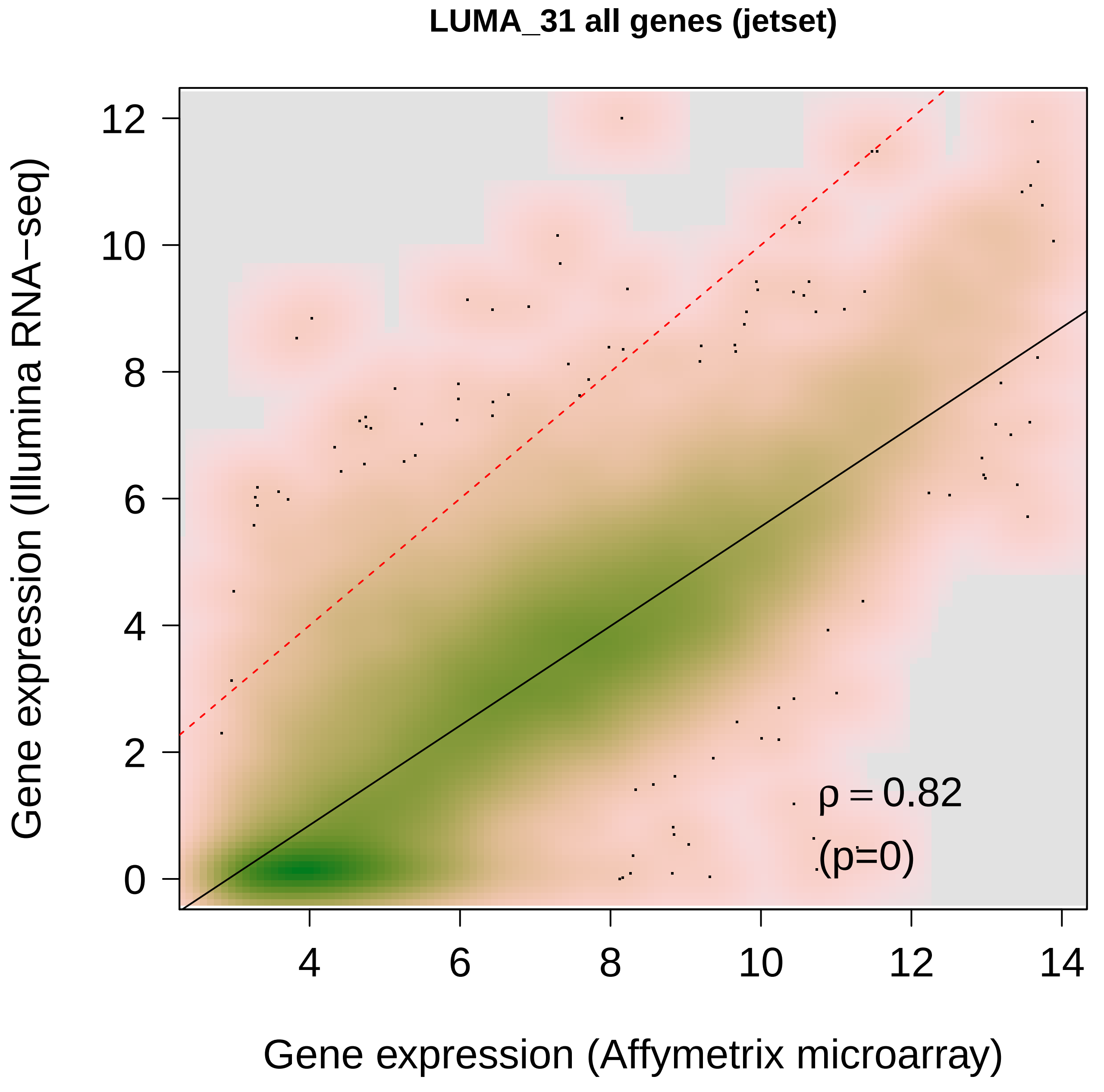

LUMA_31 all genes (jetset)
Gene expression (Illumina RNA−seq)
12
10
8
6
4
2
0
4
6
8
10
12
14
Gene expression (Affymetrix microarray)
ρ = 0.82
(p=0)

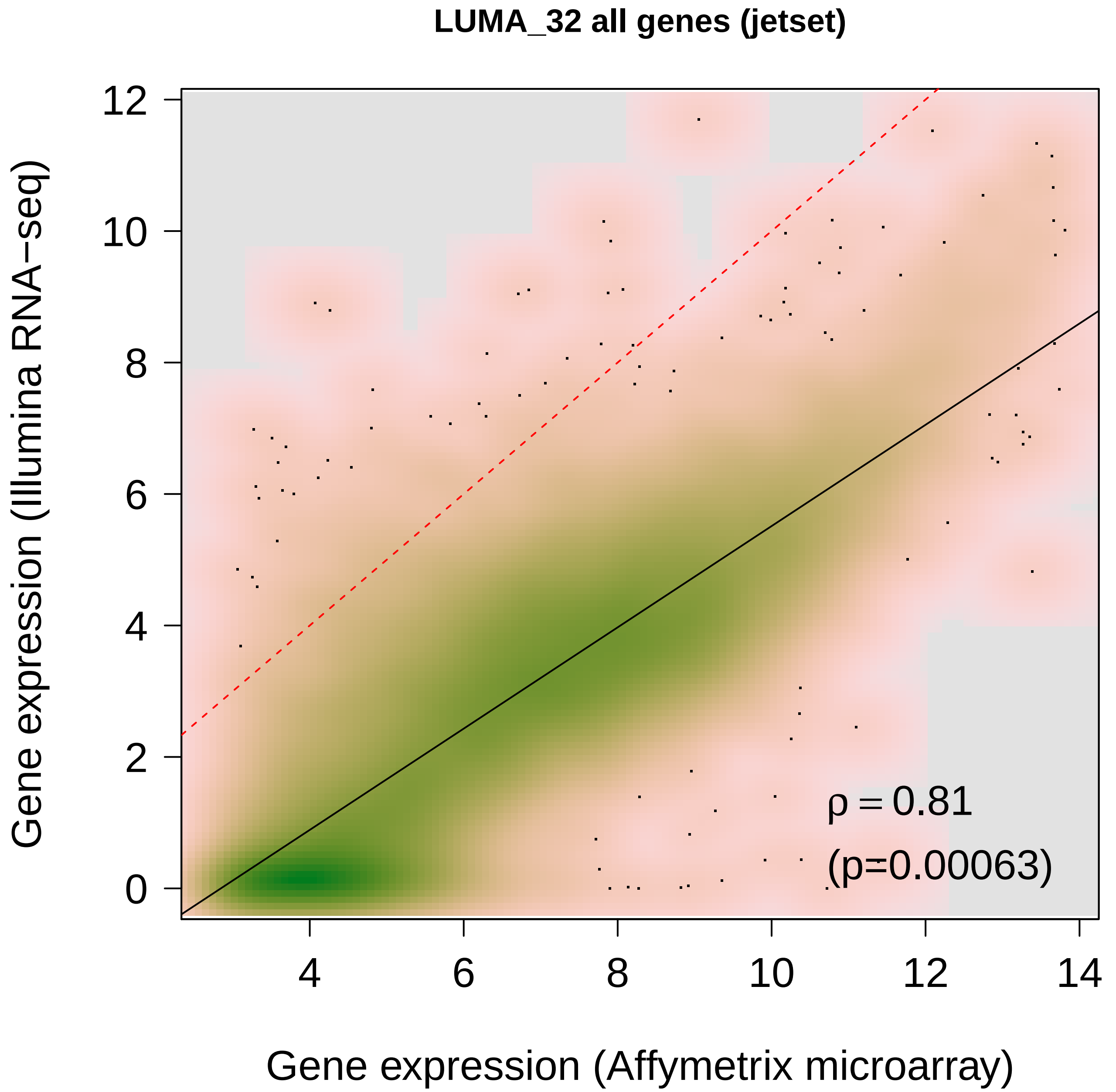

LUMA_32 all genes (jetset)
Gene expression (Illumina RNA−seq)
Gene expression (Affymetrix microarray)
ρ = 0.81
(p=0.00063)
0
2
4
6
8
10
12
4
6
8
10
12
14

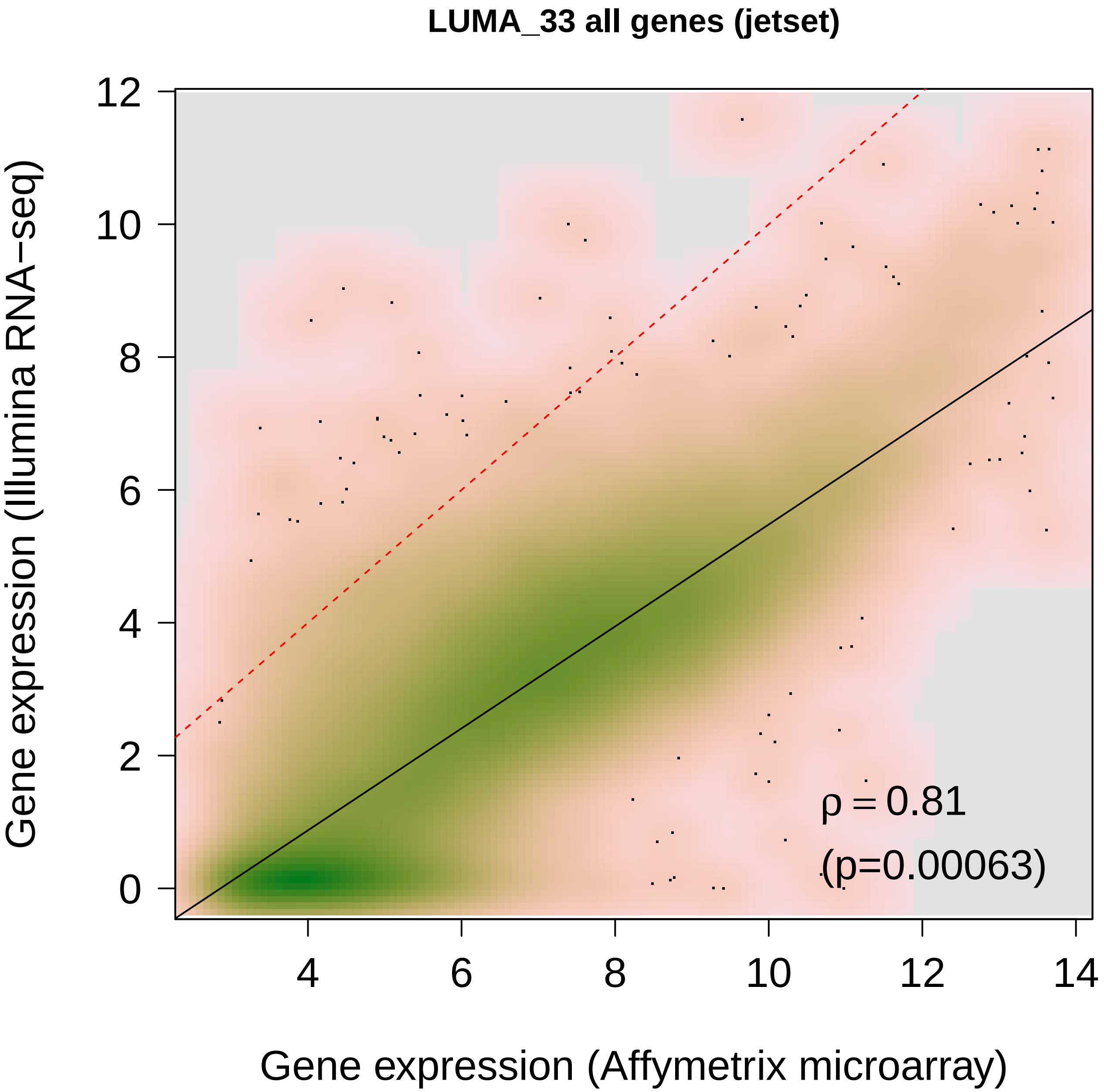
LUMA_33 all genes (jetset)
Gene expression (Illumina RNA−seq)
Gene expression (Affymetrix microarray)
ρ = 0.81
(p=0.00063)
0
2
4
6
8
10
12
4
6
8
10
12
14

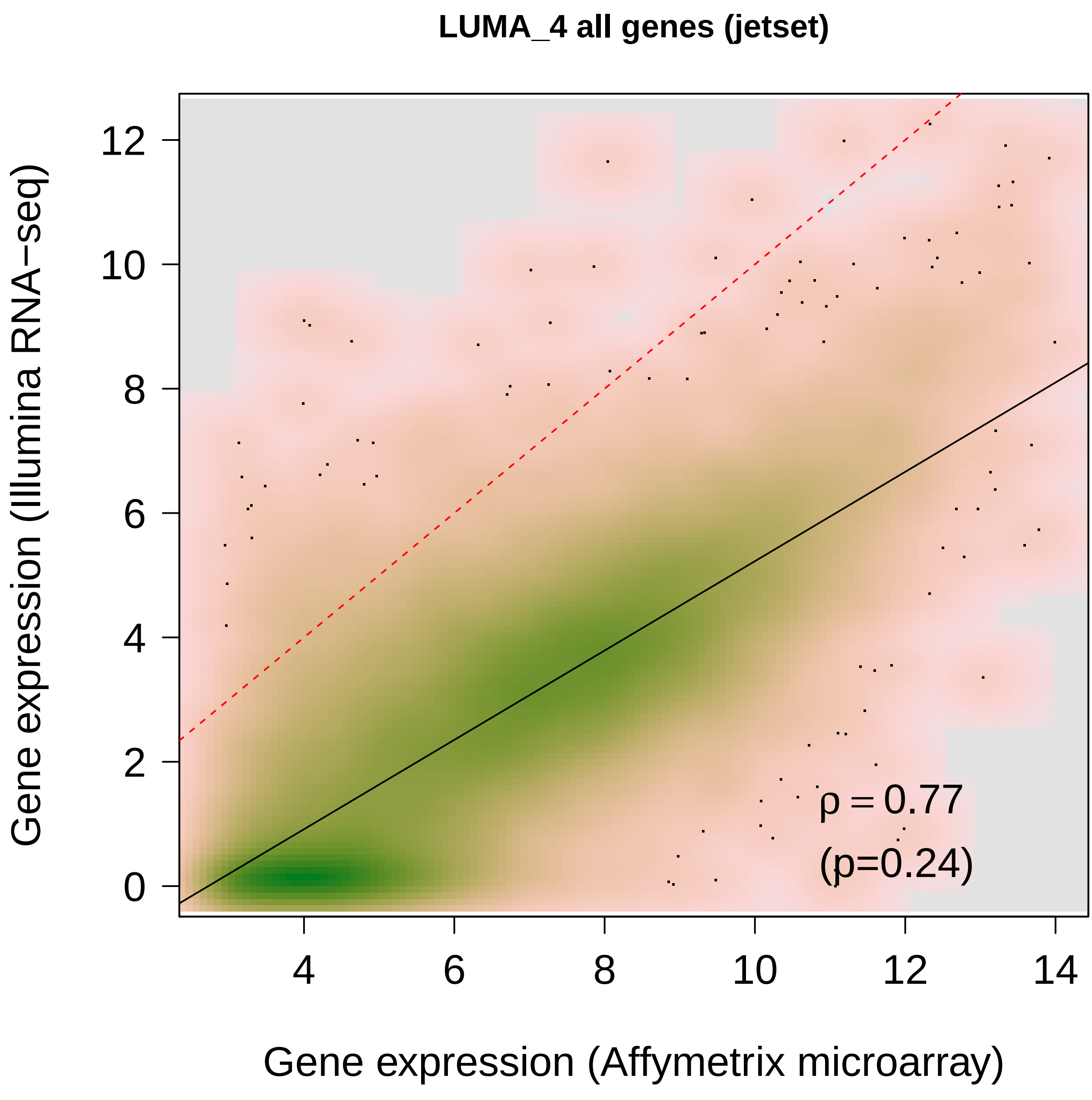

LUMA_4 all genes (jetset)
Gene expression (Illumina RNA−seq)
Gene expression (Affymetrix microarray)
ρ = 0.77
(p=0.24)
0
2
4
6
8
10
12
4
6
8
10
12
14

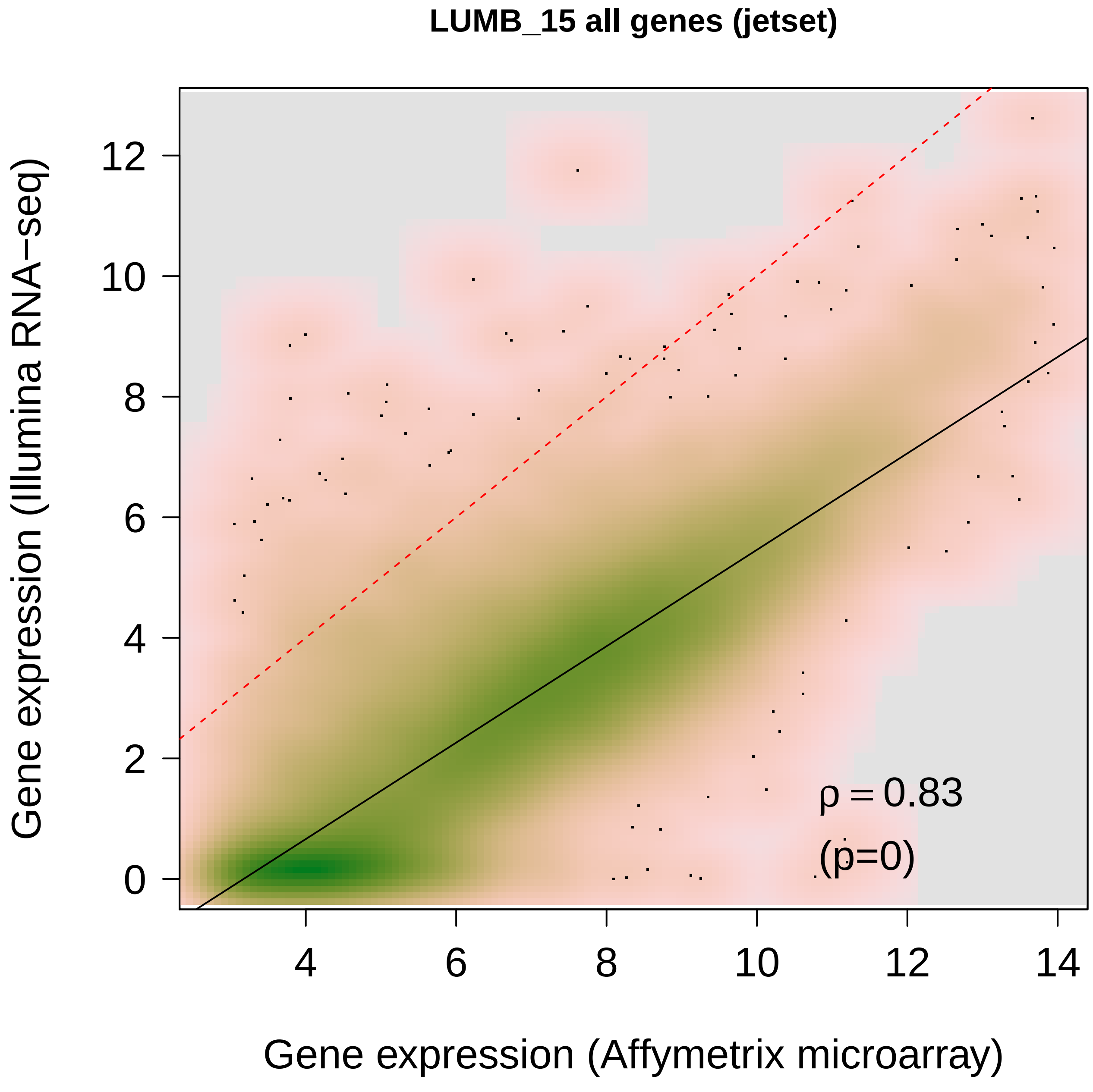

LUMB_15 all genes (jetset)
Gene expression (Illumina RNA−seq)
Gene expression (Affymetrix microarray)
ρ = 0.83
(p=0)
0
2
4
6
8
10
12
4
6
8
10
12
14

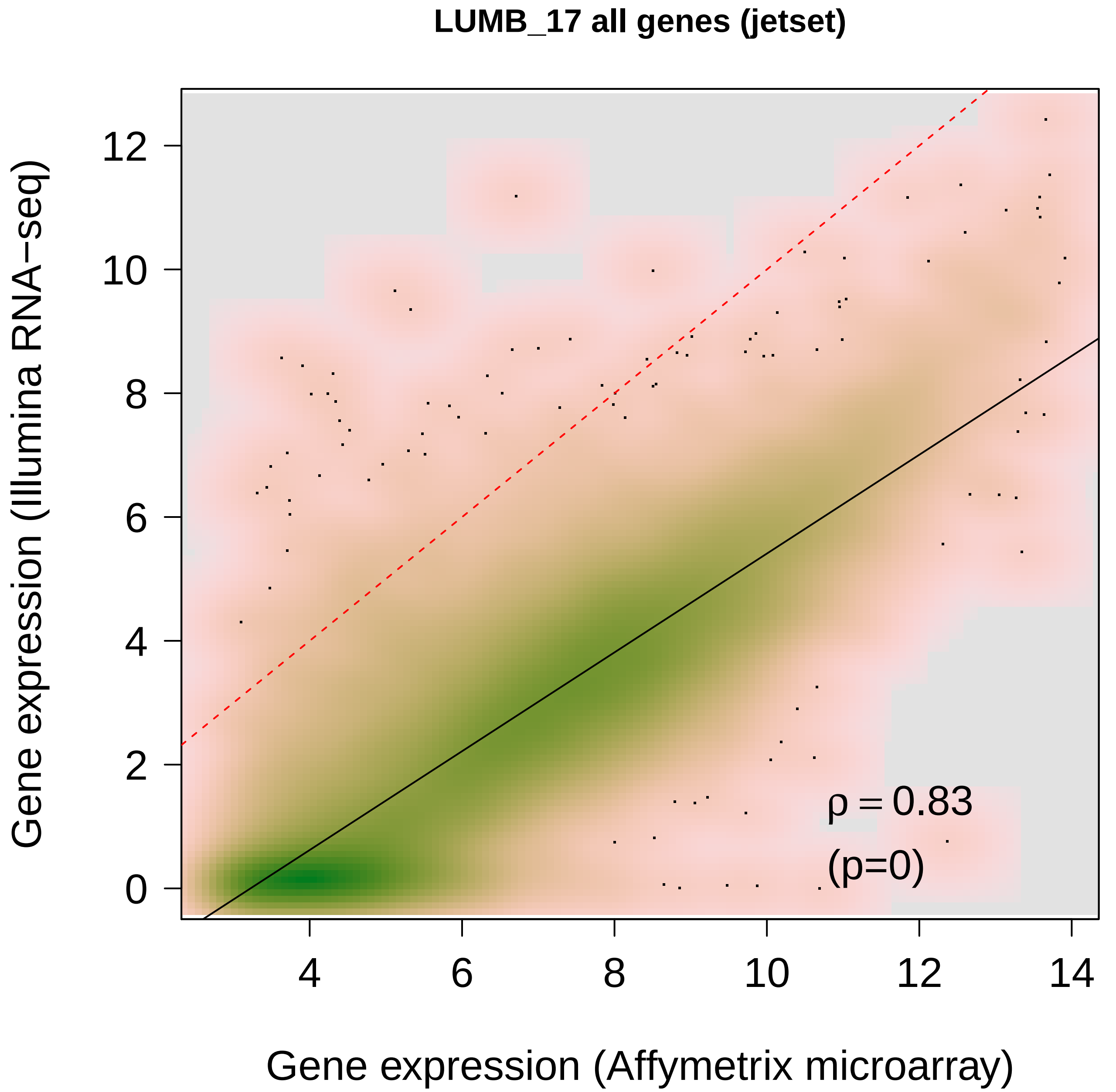

LUMB_17 all genes (jetset)
Gene expression (Illumina RNA−seq)
Gene expression (Affymetrix microarray)
ρ = 0.83
(p=0)
0
2
4
6
8
10
12
4
6
8
10
12
14

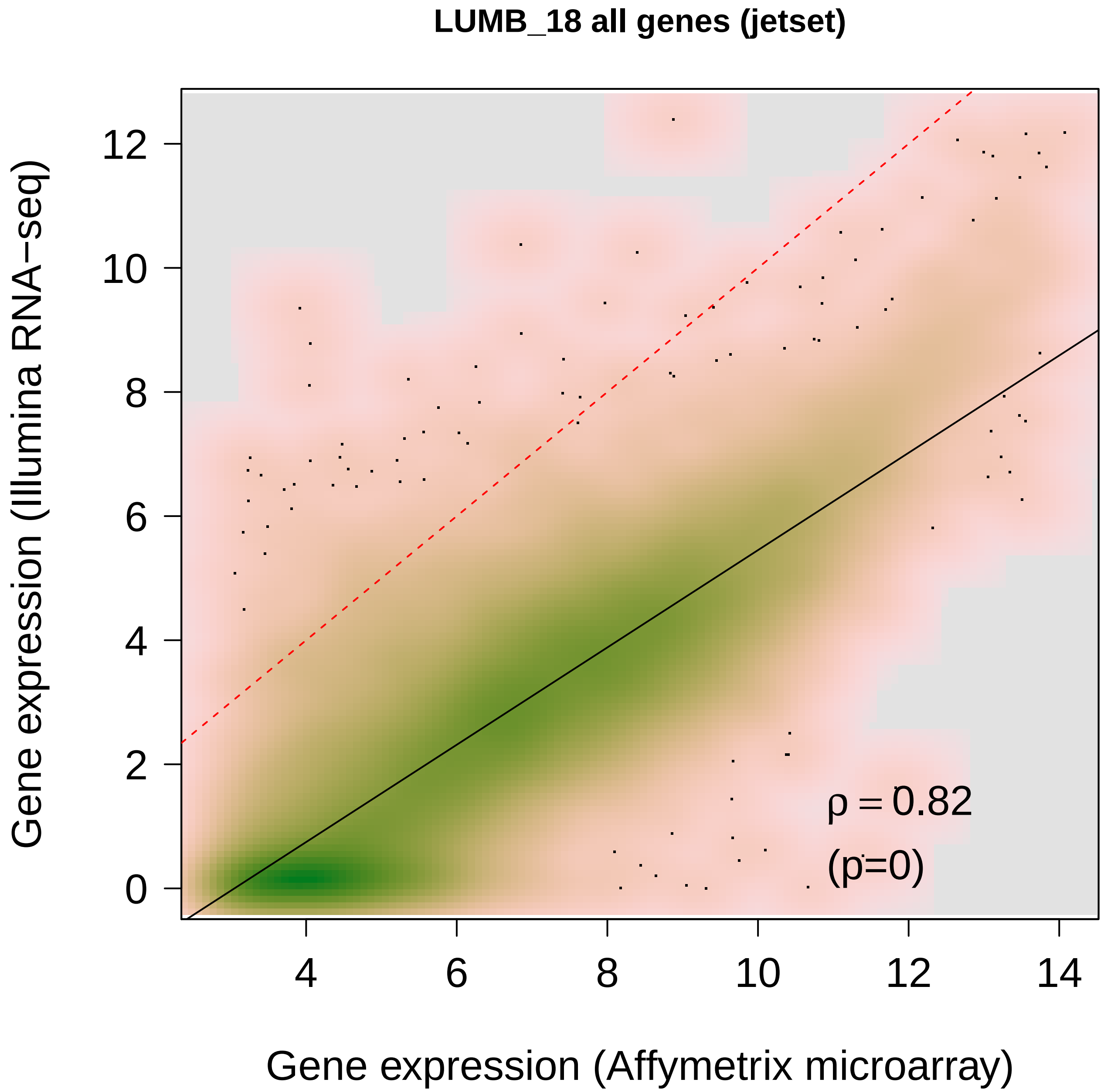

LUMB_18 all genes (jetset)
Gene expression (Illumina RNA−seq)
Gene expression (Affymetrix microarray)
ρ = 0.82
(p=0)
0
2
4
6
8
10
12
4
6
8
10
12
14

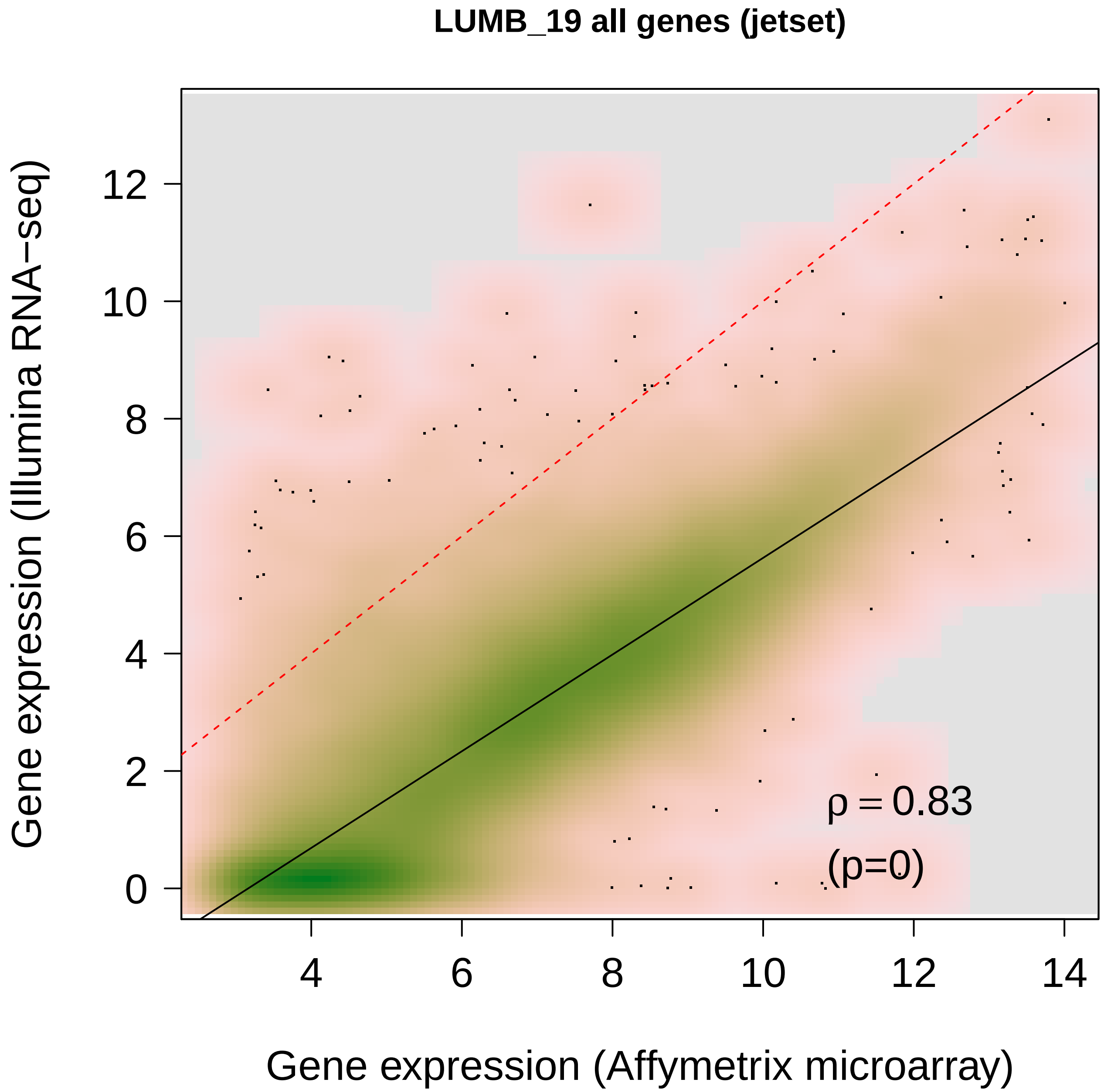

LUMB_19 all genes (jetset)
Gene expression (Illumina RNA−seq)
Gene expression (Affymetrix microarray)
ρ = 0.83
(p=0)
0
2
4
6
8
10
12
4
6
8
10
12
14

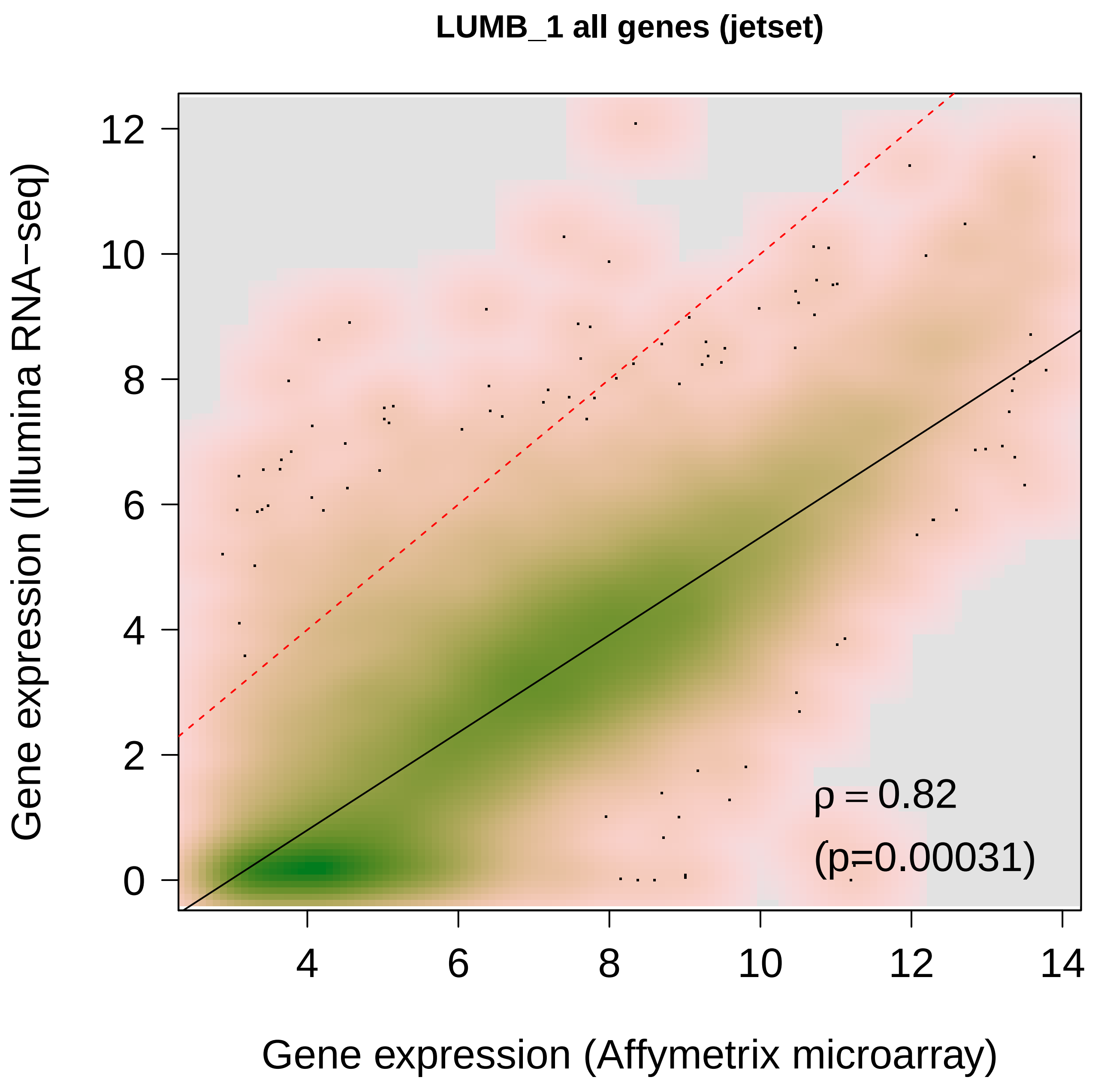

LUMB_1 all genes (jetset)
Gene expression (Illumina RNA−seq)
Gene expression (Affymetrix microarray)
0
2
4
6
8
10
12
4
6
8
10
12
14
ρ = 0.82
(p=0.00031)

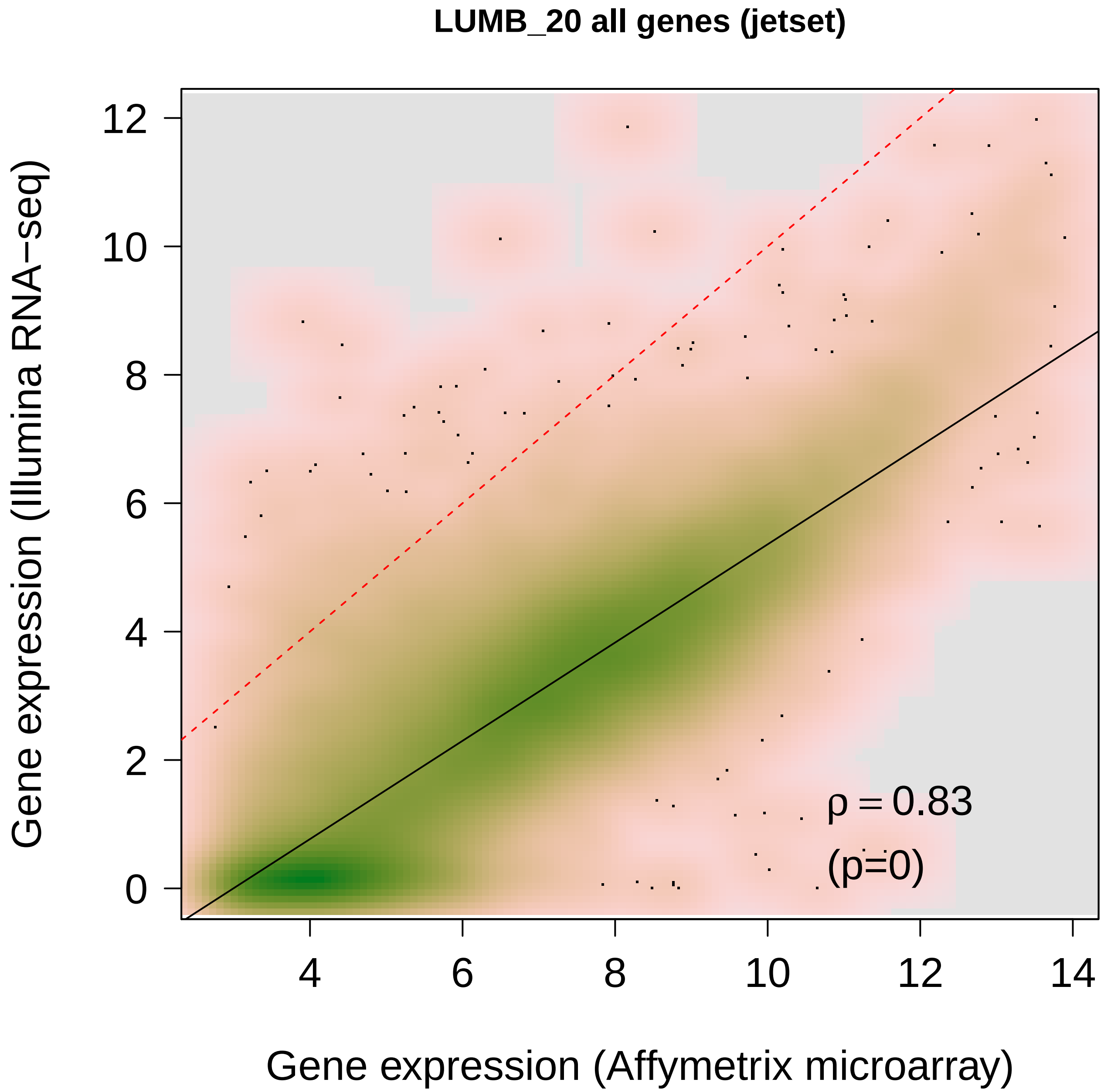

LUMB_20 all genes (jetset)
Gene expression (Illumina RNA−seq)
Gene expression (Affymetrix microarray)
ρ = 0.83
(p=0)
0
2
4
6
8
10
12
4
6
8
10
12
14

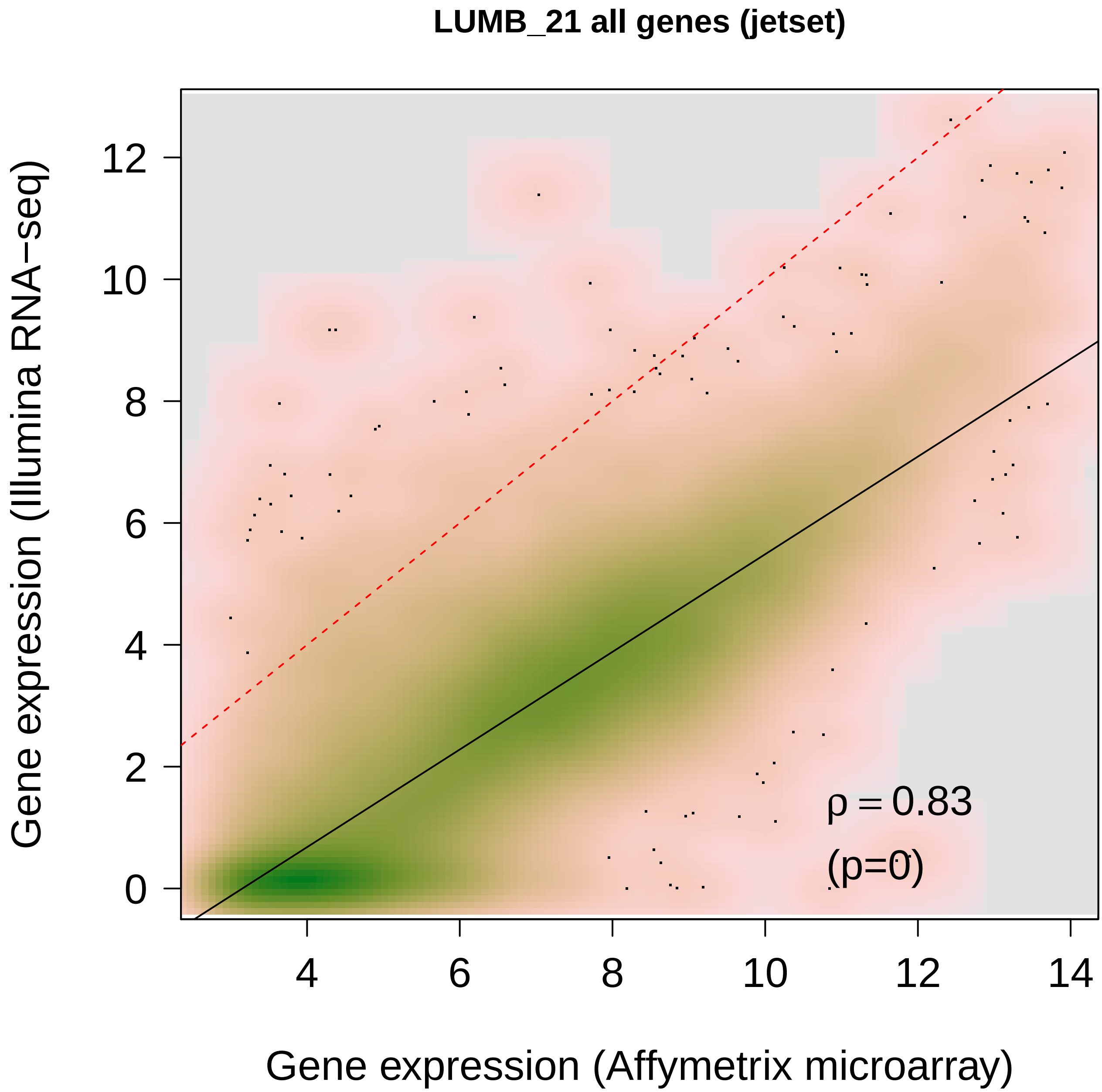

LUMB_21 all genes (jetset)
Gene expression (Illumina RNA−seq)
Gene expression (Affymetrix microarray)
ρ = 0.83
(p=0)
0
2
4
6
8
10
12
4
6
8
10
12
14

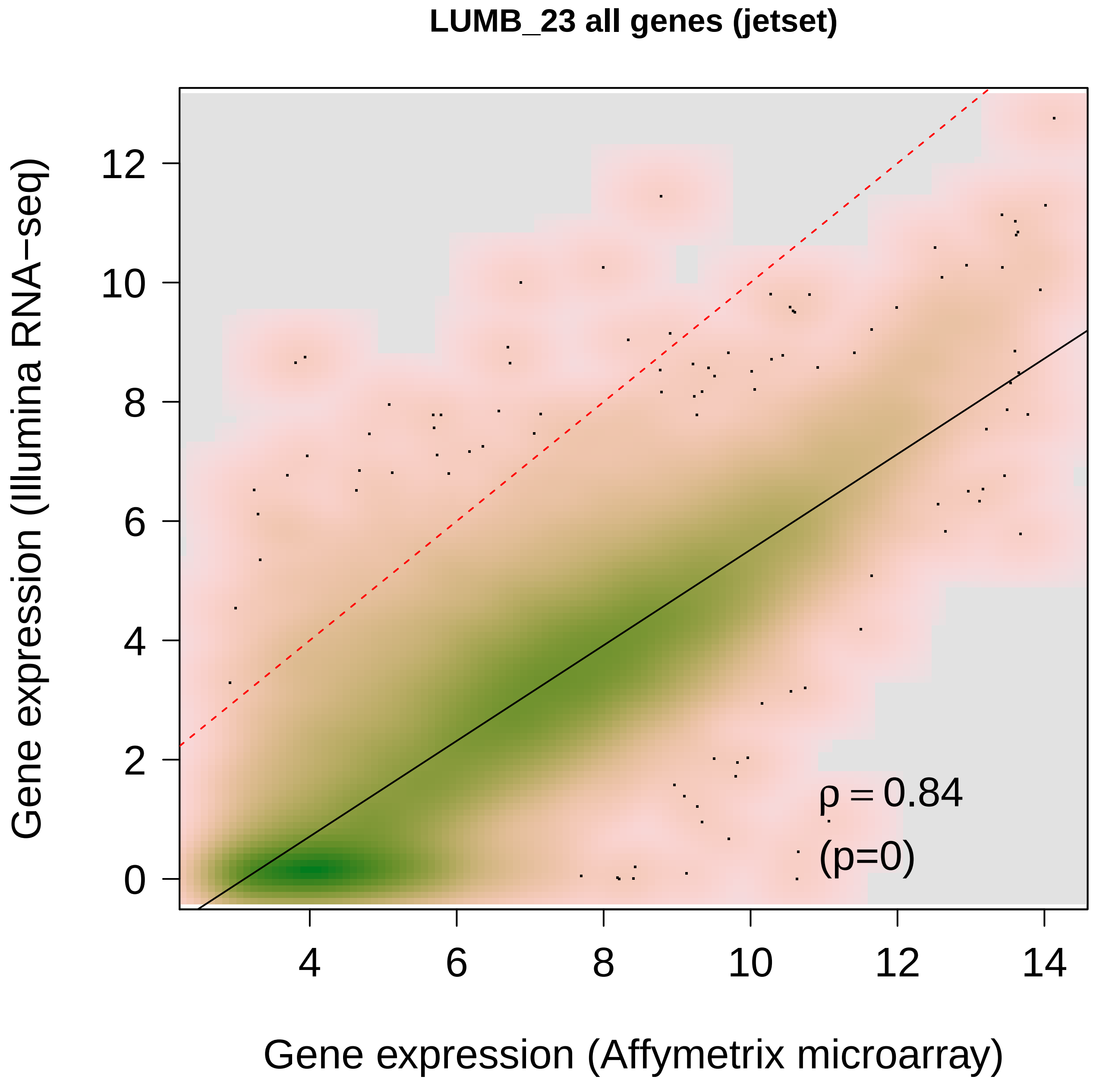

LUMB_23 all genes (jetset)
Gene expression (Illumina RNA−seq)
Gene expression (Affymetrix microarray)
0
2
4
6
8
10
12
4
6
8
10
12
14
ρ = 0.84
(p=0)

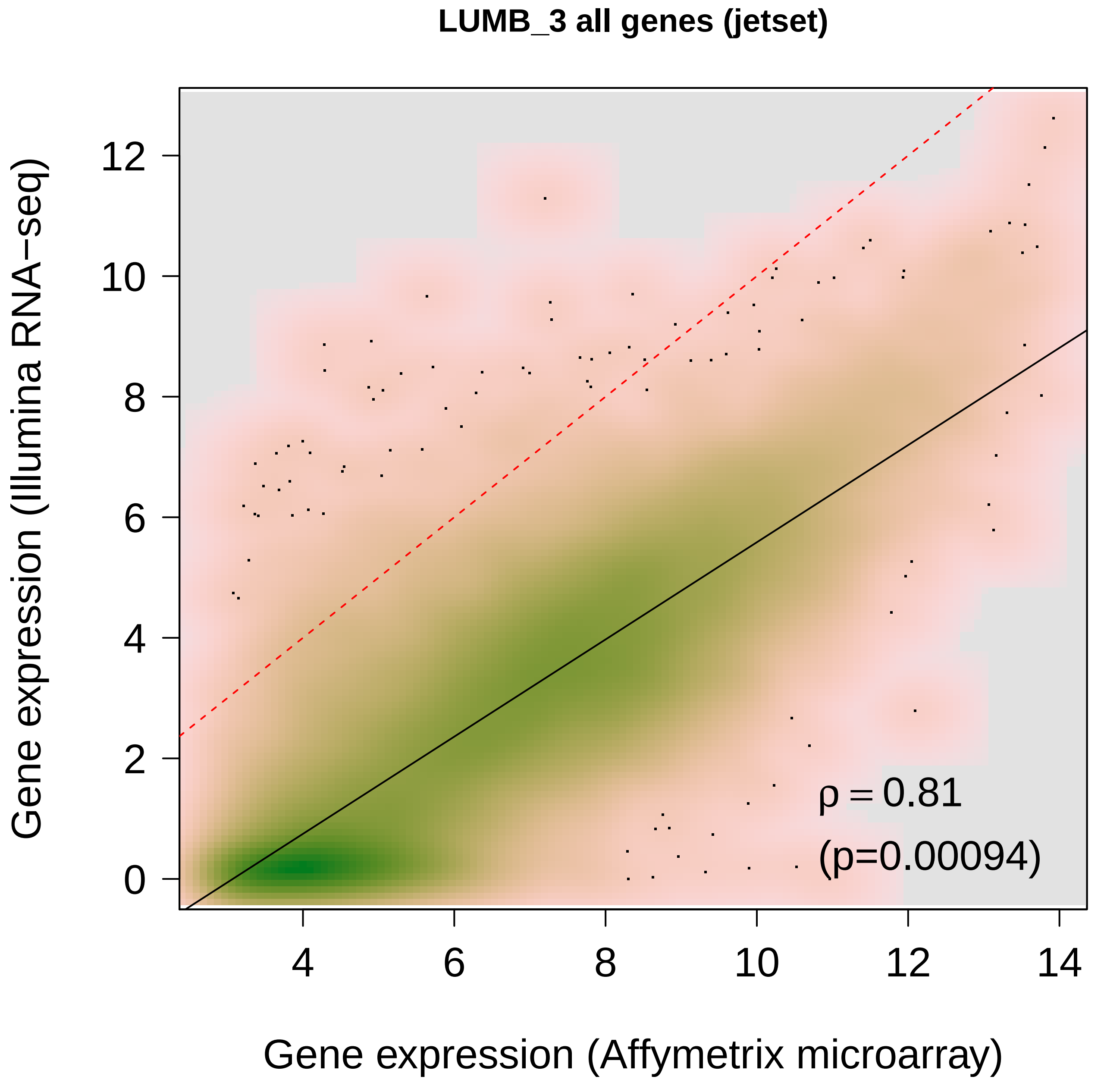
LUMB_3 all genes (jetset)
Gene expression (Illumina RNA−seq)
Gene expression (Affymetrix microarray)
0
2
4
6
8
10
12
4
6
8
10
12
14
ρ = 0.81
(p=0.00094)

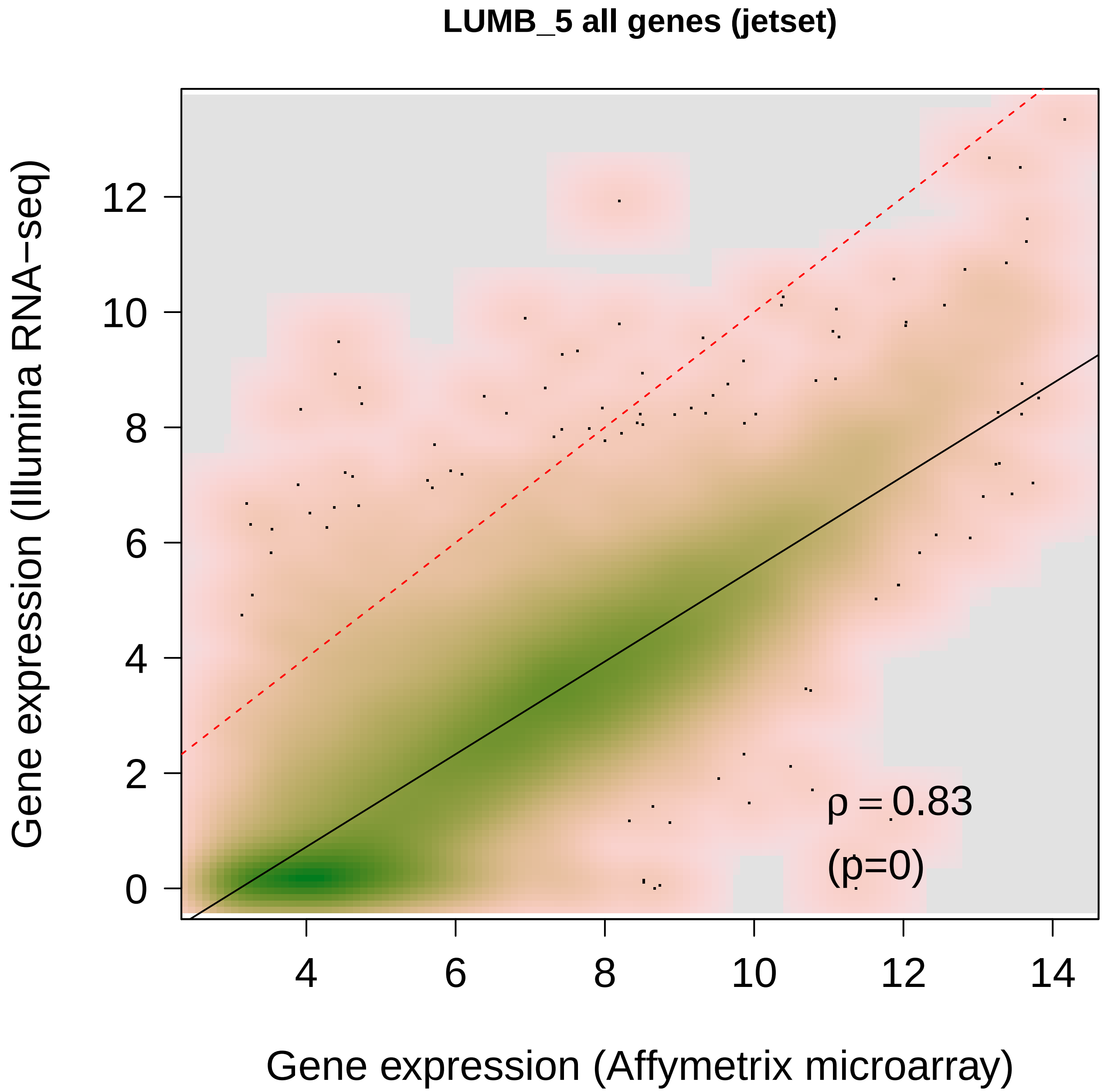
LUMB_5 all genes (jetset)
Gene expression (Illumina RNA−seq)
Gene expression (Affymetrix microarray)
0
2
4
6
8
10
12
4
6
8
10
12
14
ρ = 0.83
(p=0)

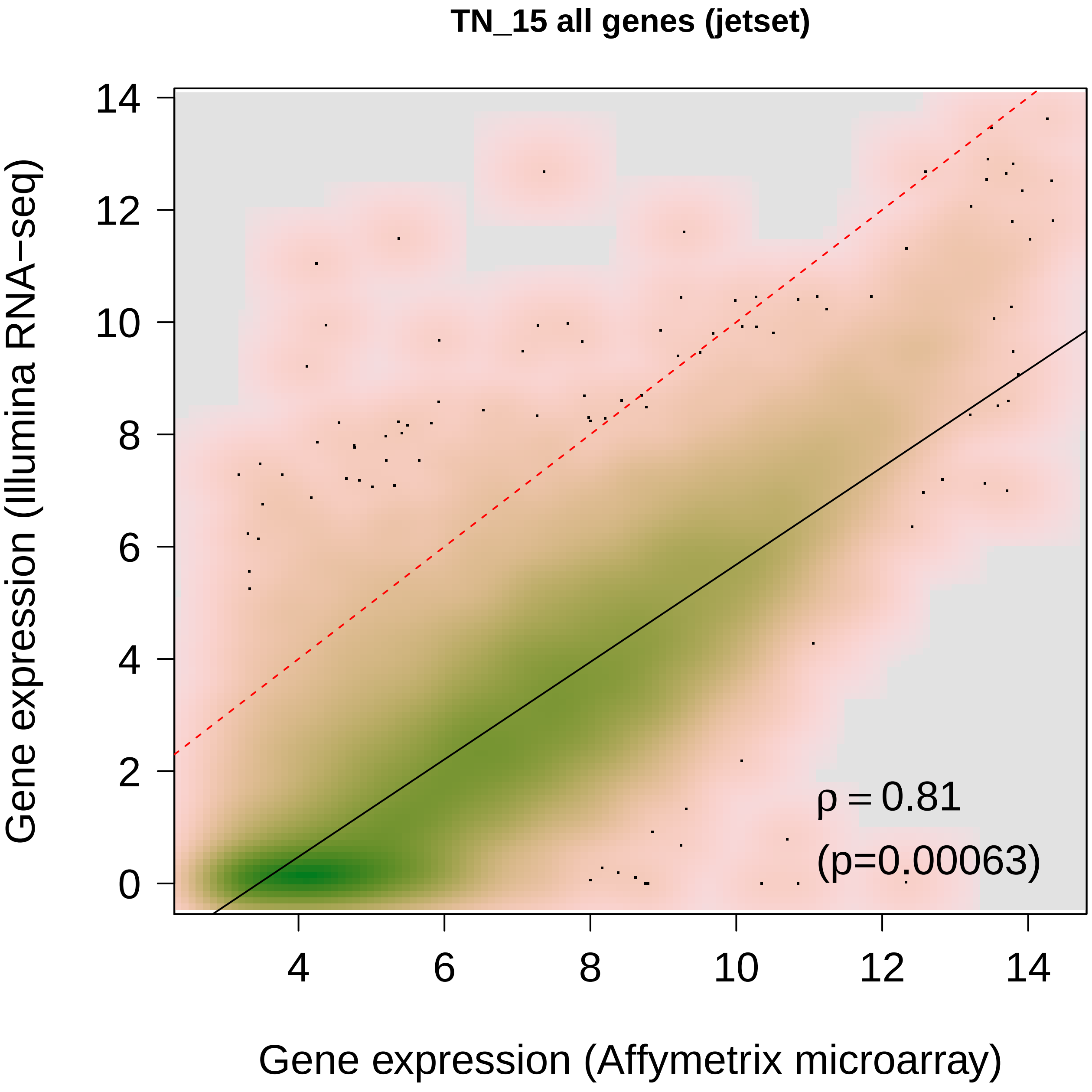

TN_15 all genes (jetset)
Gene expression (Illumina RNA−seq)
Gene expression (Affymetrix microarray)
ρ = 0.81
(p=0.00063)
0
2
4
6
8
10
12
14
4
6
8
10
12
14

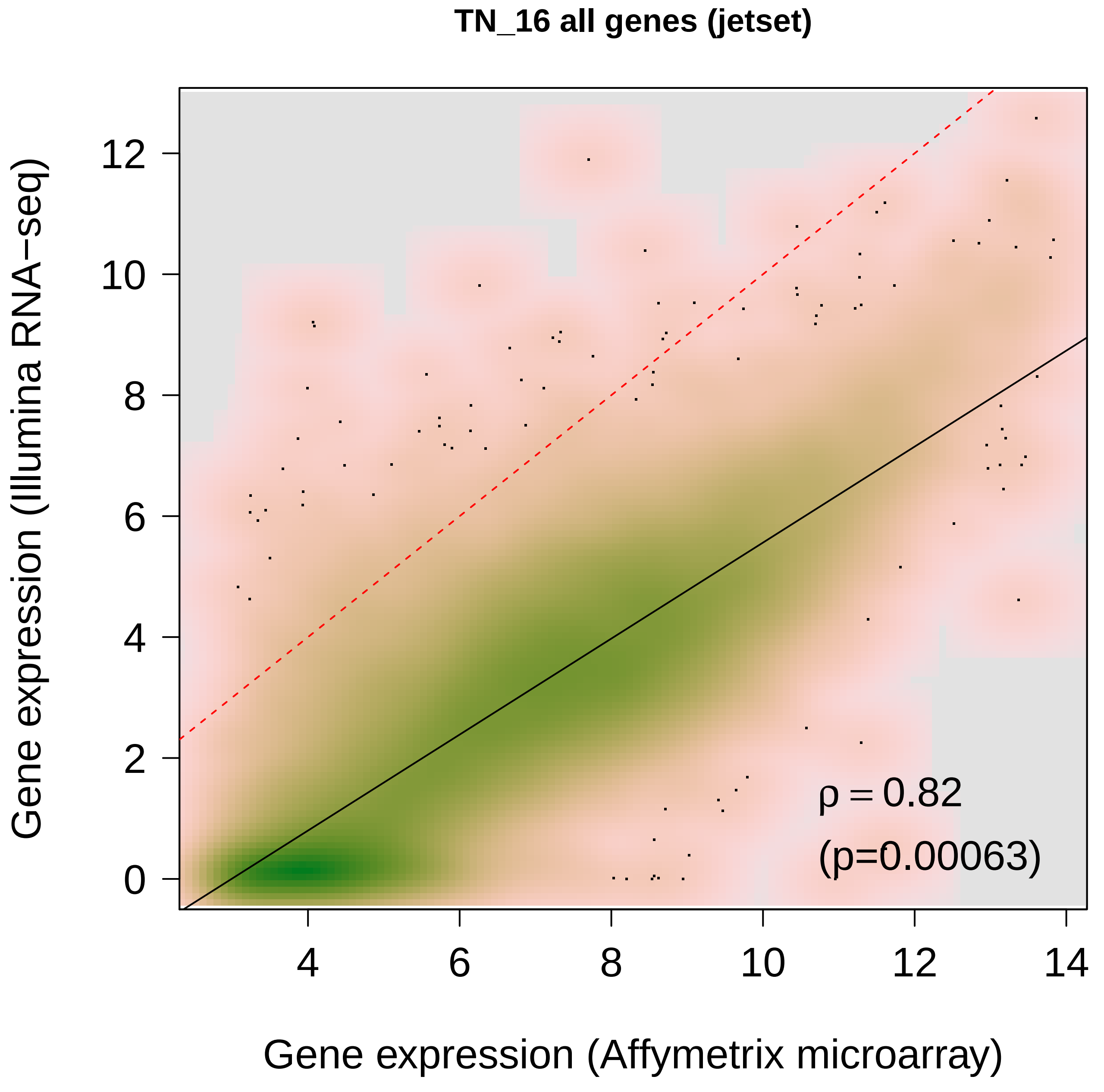
TN_16 all genes (jetset)
Gene expression (Illumina RNA−seq)
Gene expression (Affymetrix microarray)
ρ = 0.82
(p=0.00063)
0
2
4
6
8
10
12
4
6
8
10
12
14

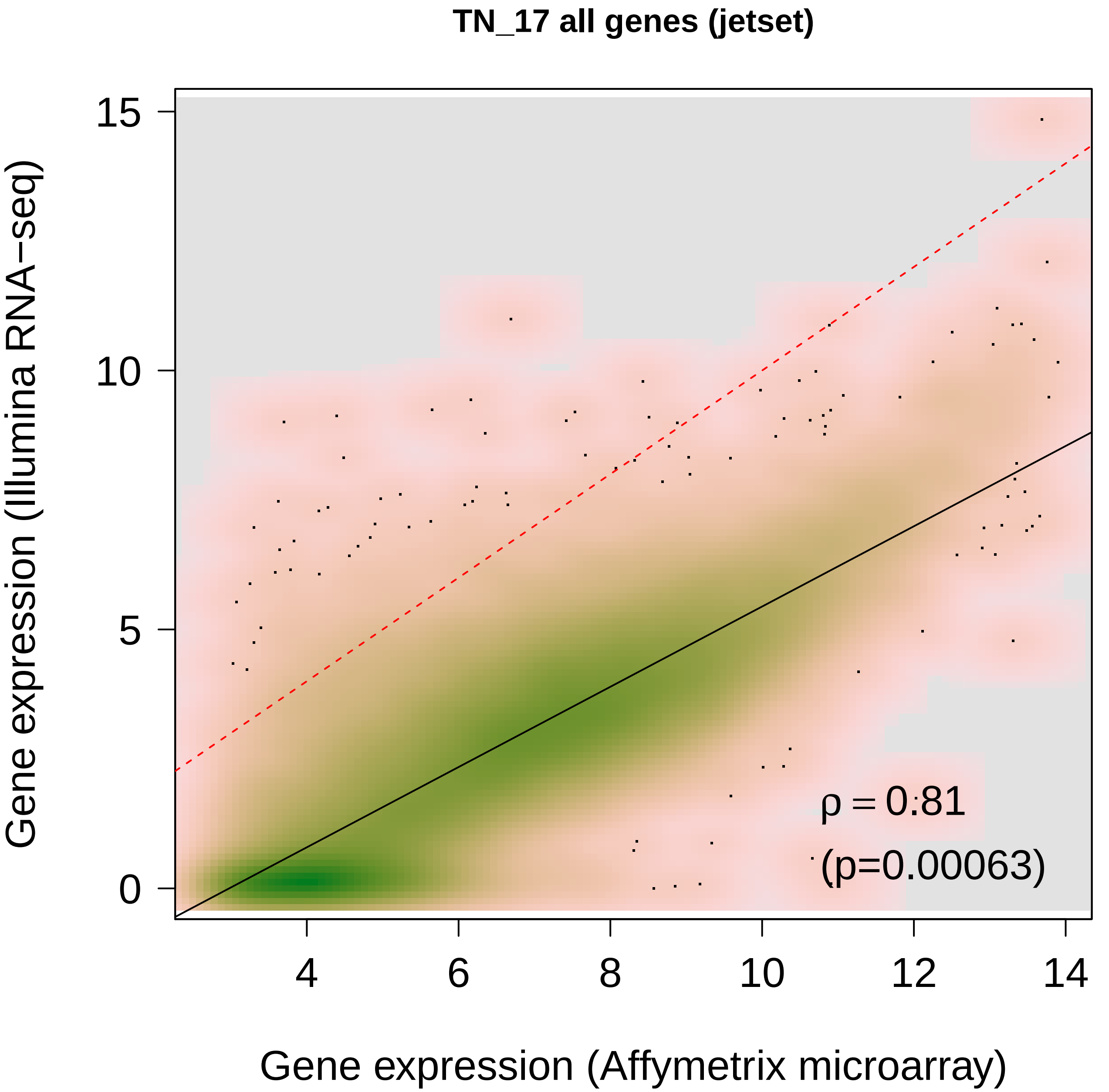

TN_17 all genes (jetset)
Gene expression (Illumina RNA−seq)
Gene expression (Affymetrix microarray)
15
10
5
0
4
6
8
10
12
14
ρ = 0.81
(p=0.00063)

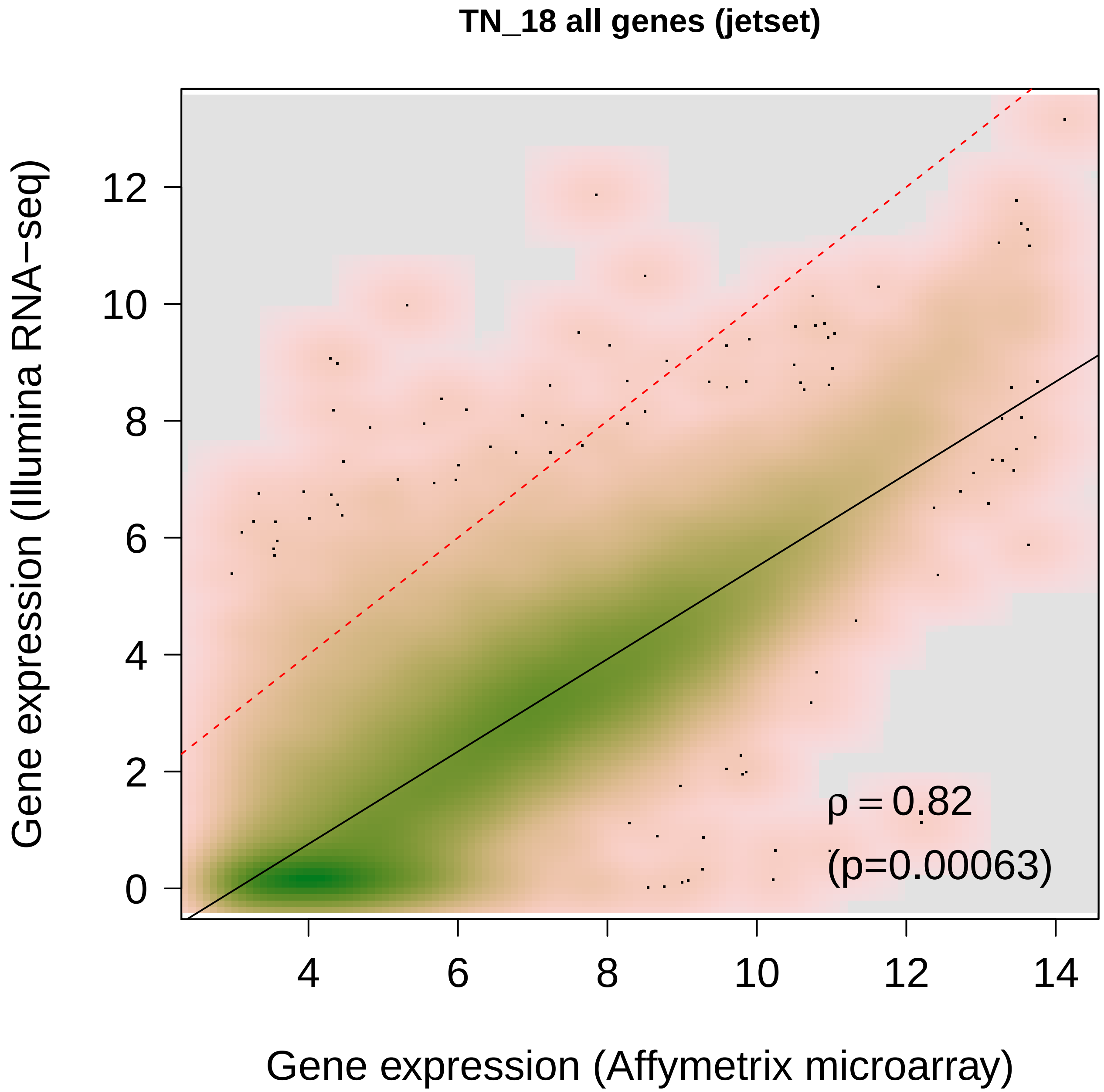
TN_18 all genes (jetset)
Gene expression (Illumina RNA−seq)
0
2
4
6
8
10
12
4
6
8
10
12
14
ρ = 0.82
(p=0.00063)
Gene expression (Affymetrix microarray)

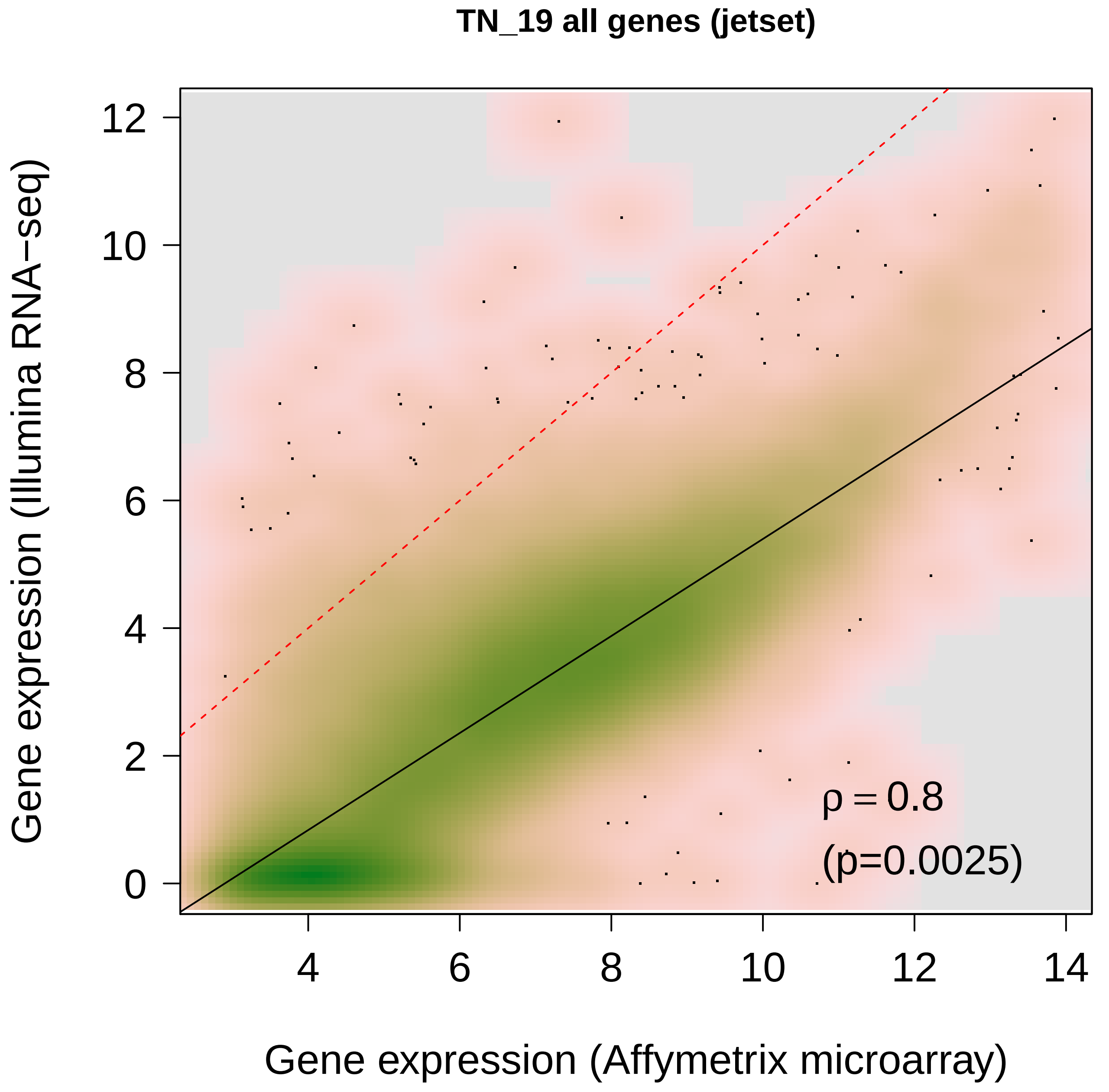

TN_19 all genes (jetset)
Gene expression (Illumina RNA−seq)
Gene expression (Affymetrix microarray)
12
10
8
6
4
2
0
4
6
8
10
12
14
ρ = 0.8
(p=0.0025)

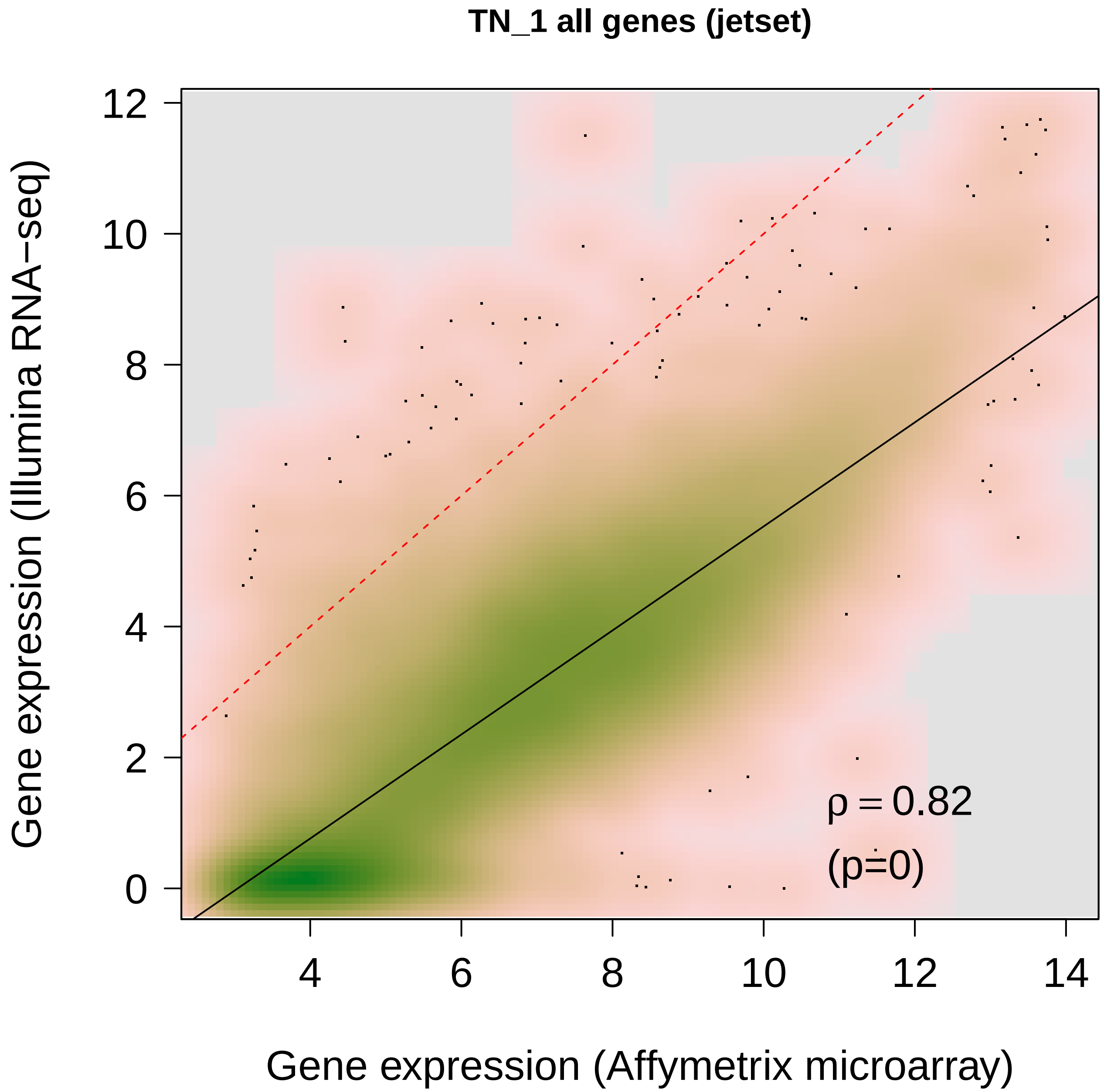

TN_1 all genes (jetset)
Gene expression (Illumina RNA−seq)
Gene expression (Affymetrix microarray)
0
2
4
6
8
10
12
4
6
8
10
12
14
ρ = 0.82
(p=0)

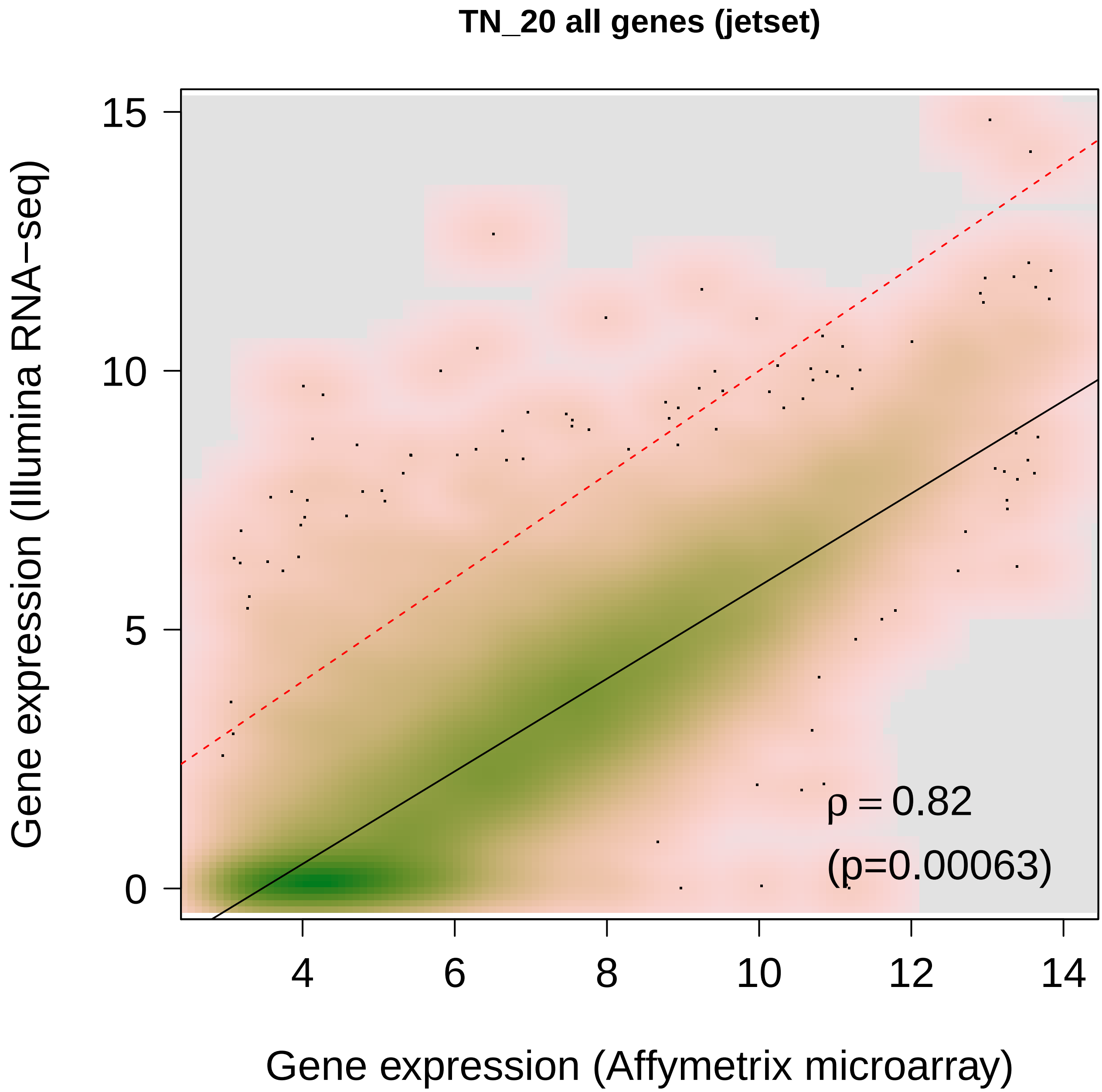
TN_20 all genes (jetset)
Gene expression (Illumina RNA−seq)
15
10
5
0
4
6
8
10
12
14
ρ = 0.82
(p=0.00063)
Gene expression (Affymetrix microarray)

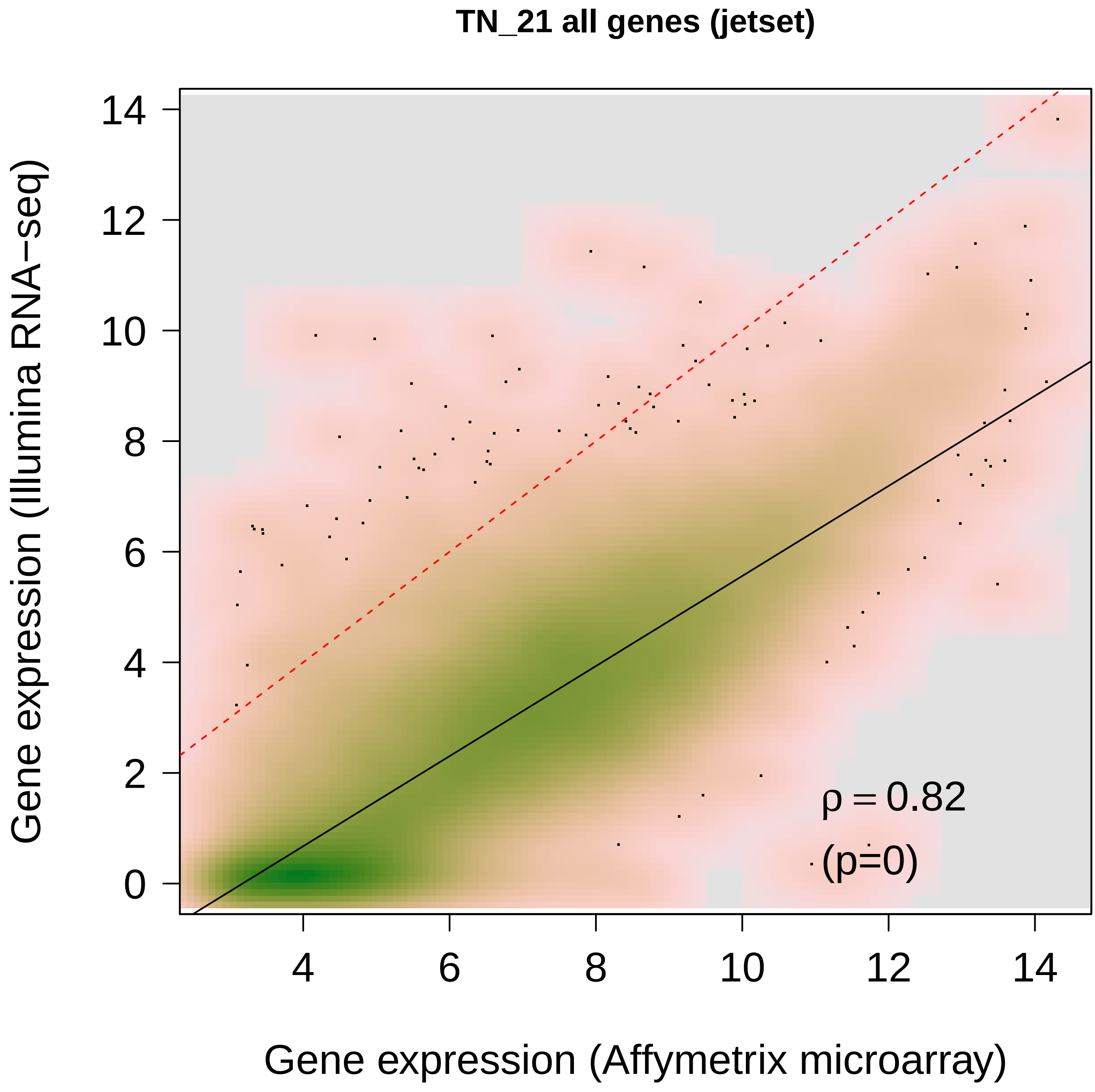
TN_21 all genes (jetset)
Gene expression (Illumina RNA−seq)
Gene expression (Affymetrix microarray)
ρ = 0.82
(p=0)
0
2
4
6
8
10
12
14
4
6
8
10
12
14

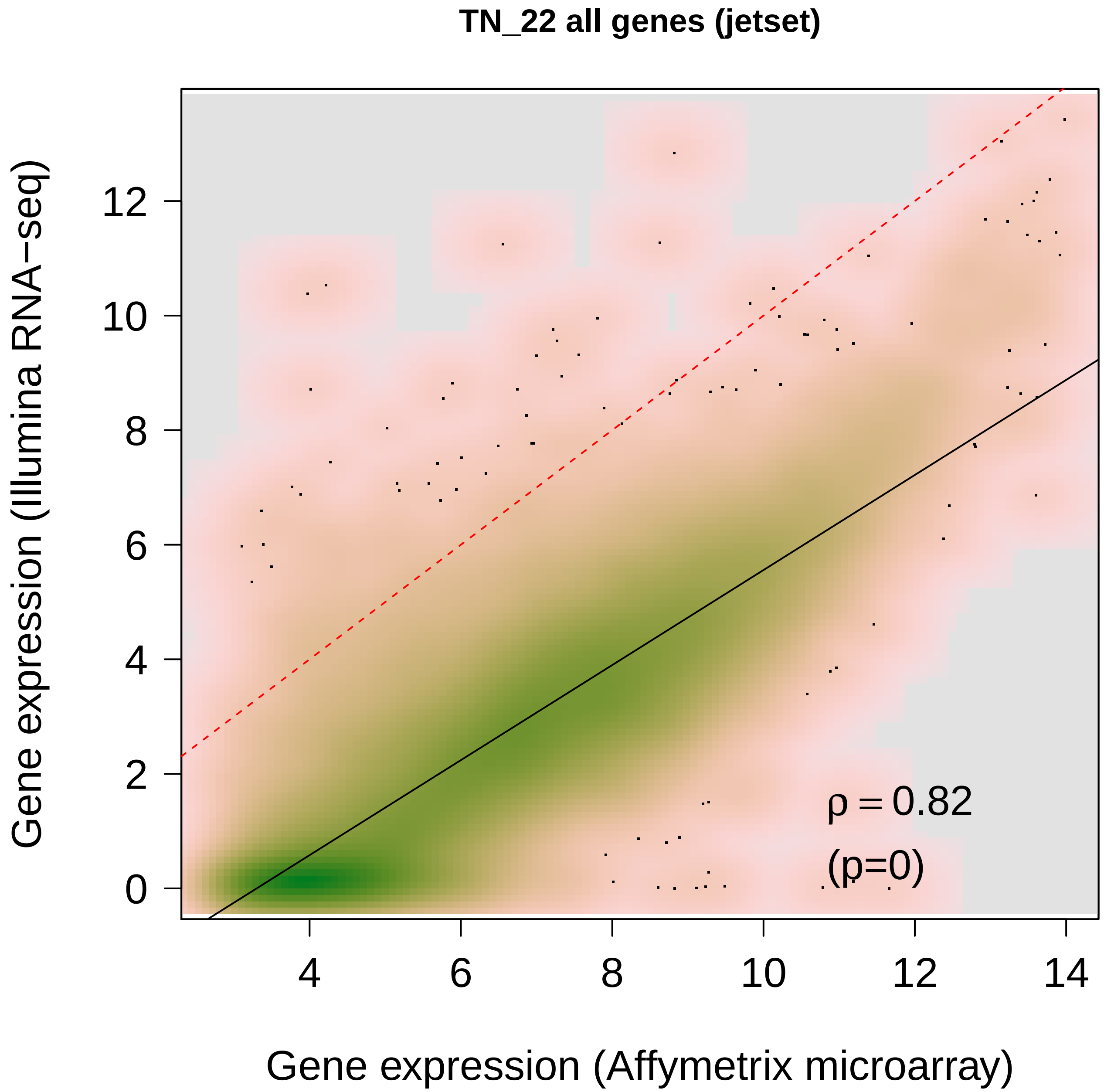
TN_22 all genes (jetset)
Gene expression (Illumina RNA−seq)
0
2
4
6
8
10
12
4
6
8
10
12
14
Gene expression (Affymetrix microarray)
ρ = 0.82
(p=0)

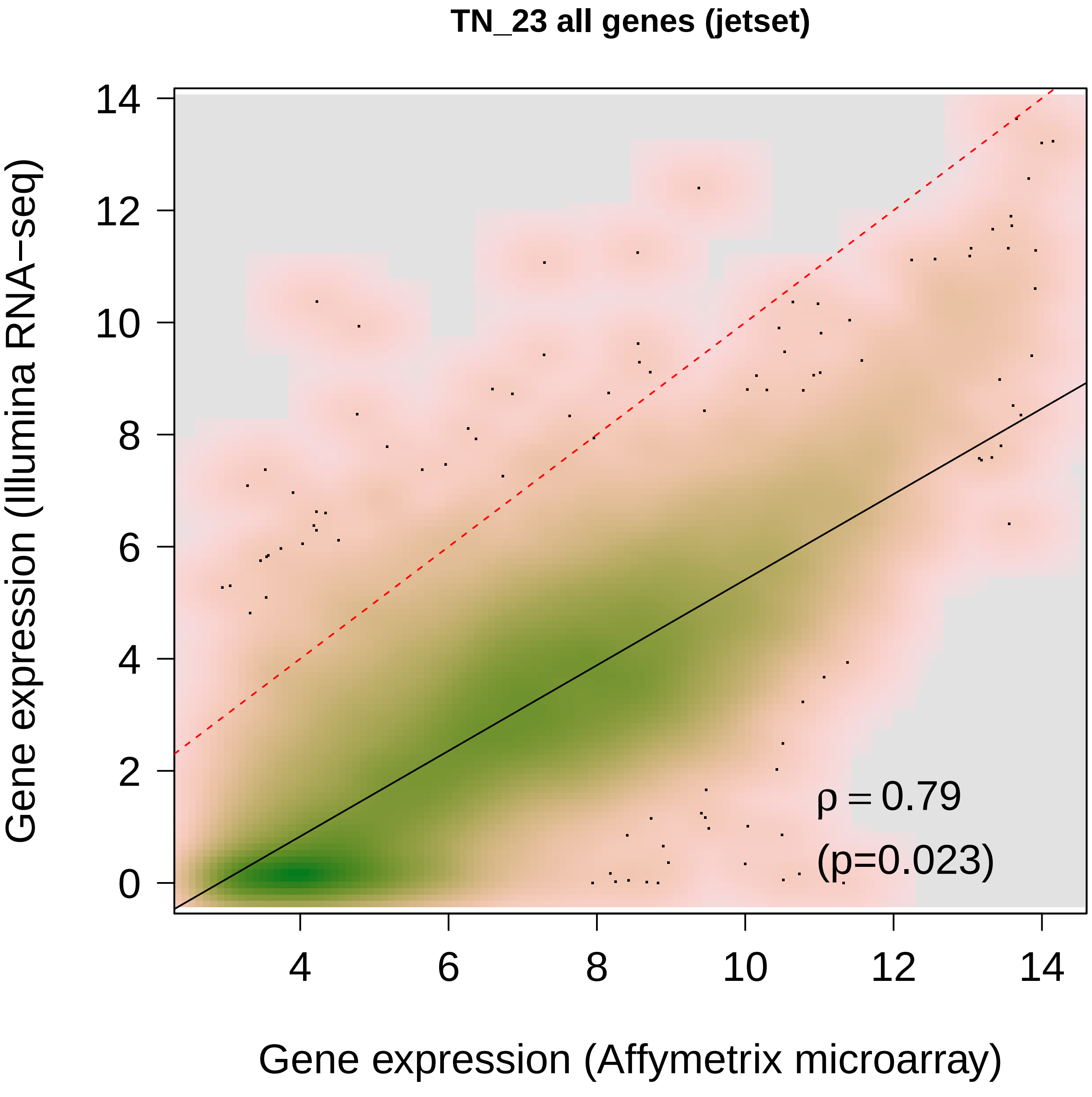
TN_23 all genes (jetset)
Gene expression (Illumina RNA−seq)
Gene expression (Affymetrix microarray)
ρ = 0.79
(p=0.023)
0
2
4
6
8
10
12
14
4
6
8
10
12
14

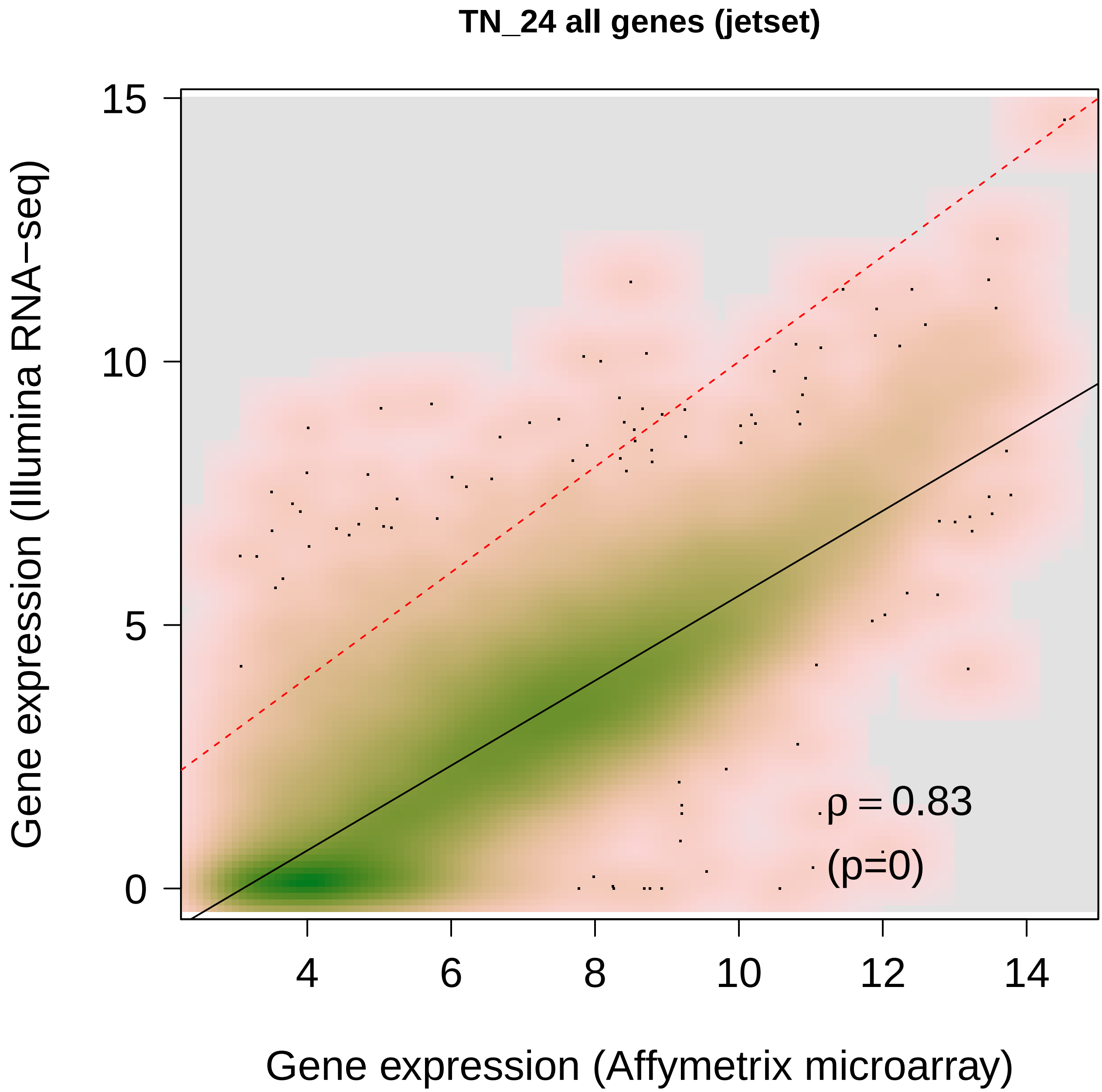

TN_24 all genes (jetset)
Gene expression (Illumina RNA−seq)
Gene expression (Affymetrix microarray)
15
10
5
0
4
6
8
10
12
14
ρ = 0.83
(p=0)

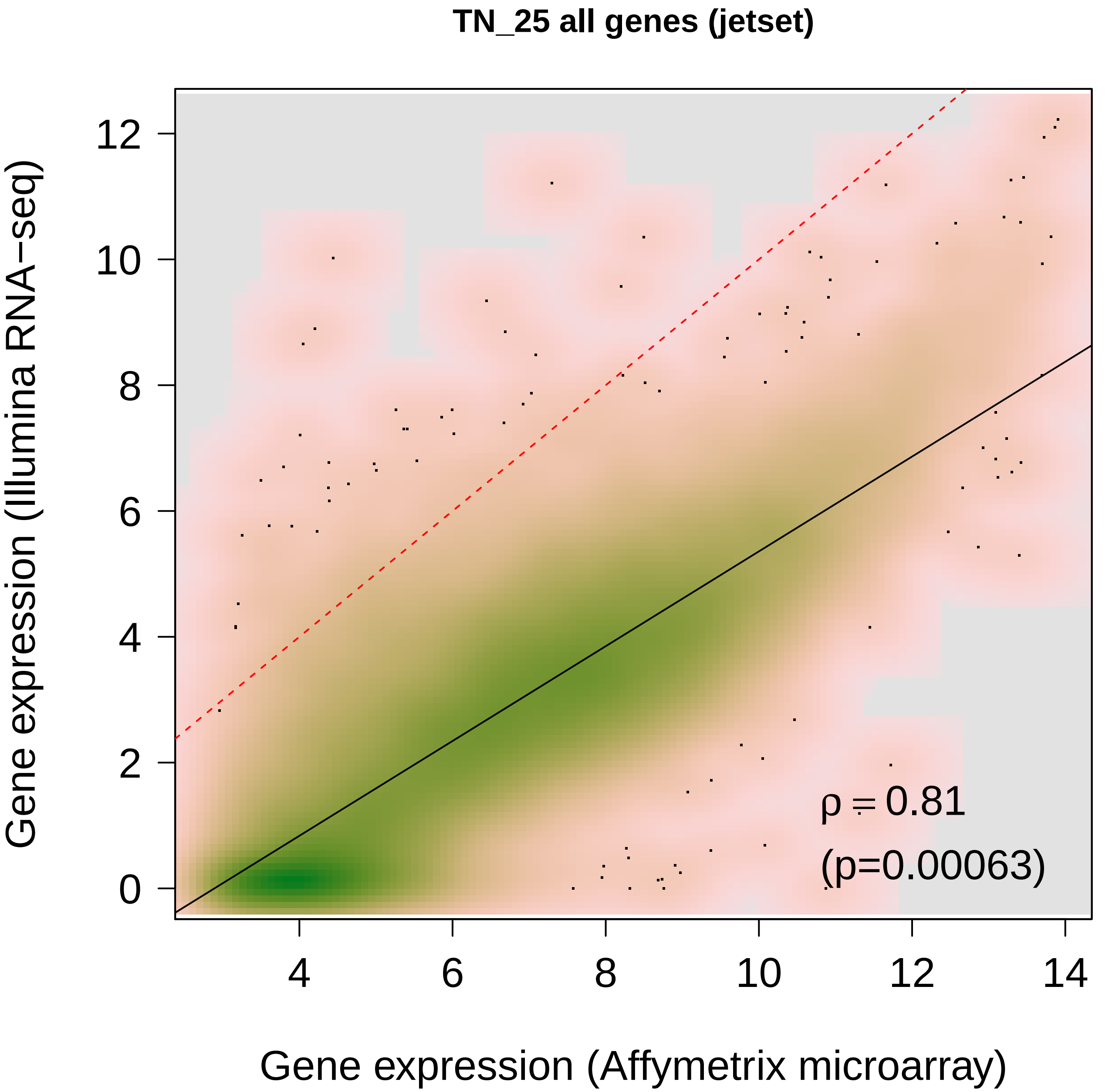

TN_25 all genes (jetset)
Gene expression (Illumina RNA−seq)
Gene expression (Affymetrix microarray)
0
2
4
6
8
10
12
4
6
8
10
12
14
ρ = 0.81
(p=0.00063)

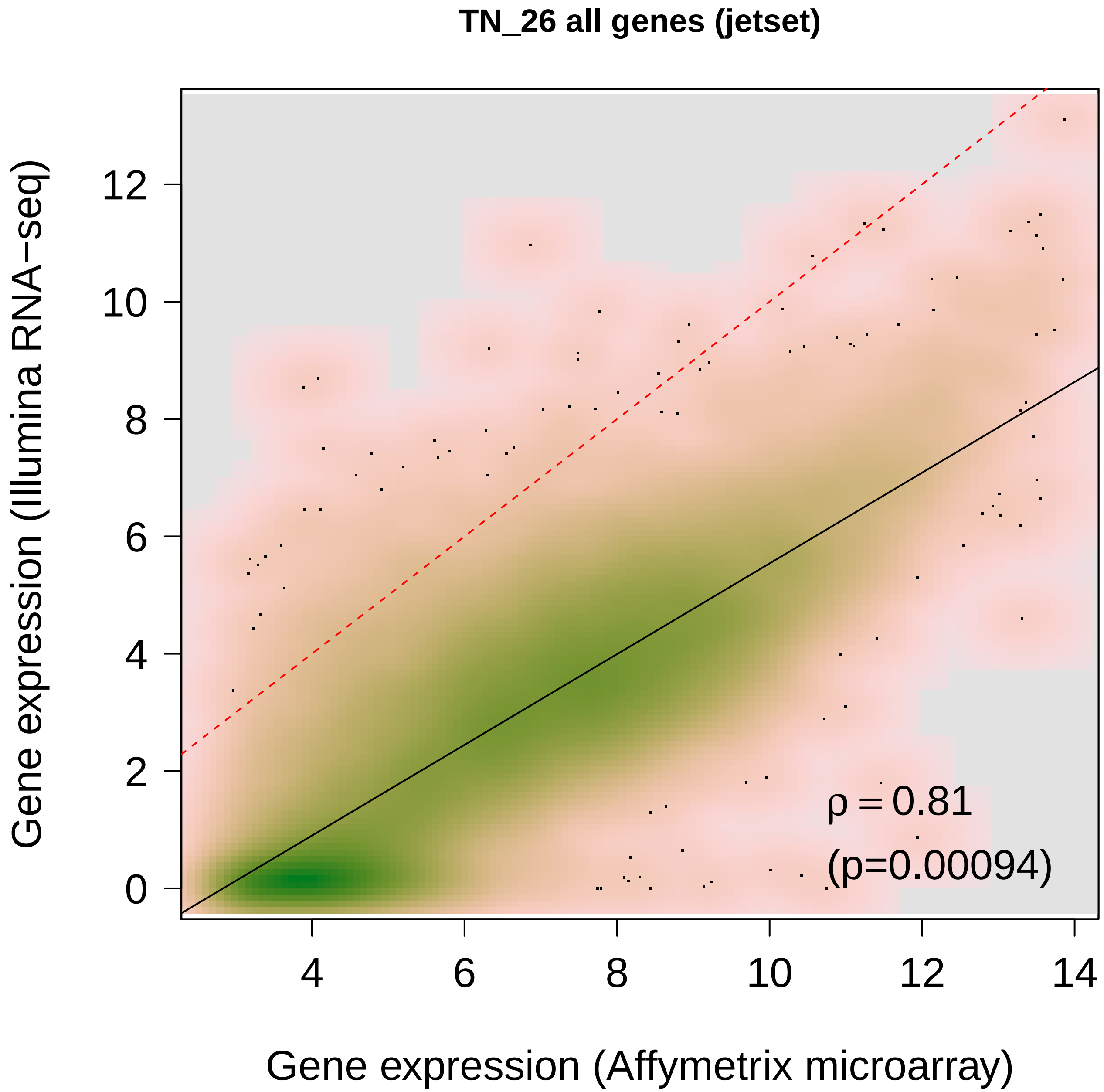
TN_26 all genes (jetset)
Gene expression (Illumina RNA−seq)
Gene expression (Affymetrix microarray)
ρ = 0.81
(p=0.00094)
0
2
4
6
8
10
12
4
6
8
10
12
14

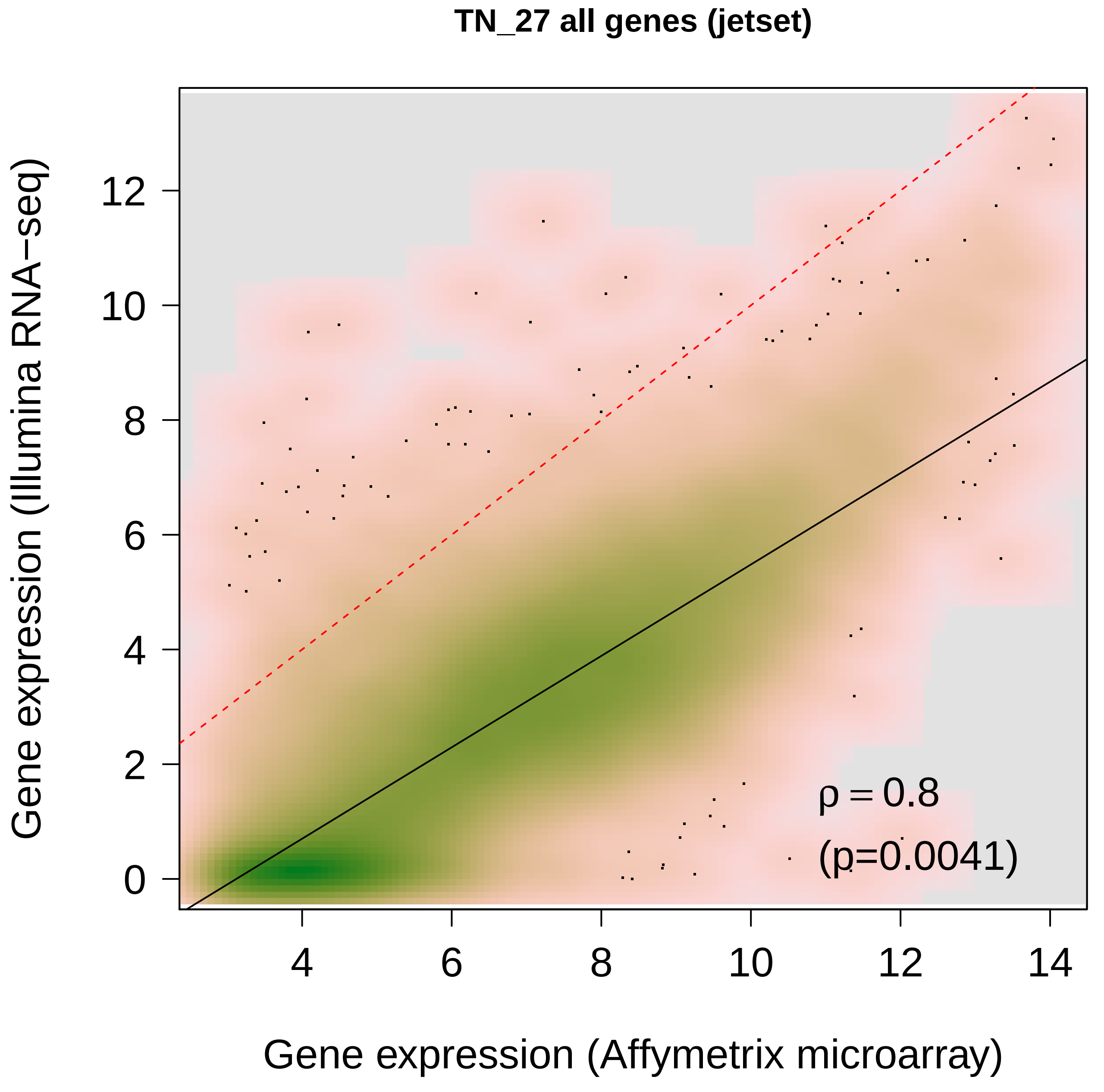
TN_27 all genes (jetset)
Gene expression (Illumina RNA−seq)
Gene expression (Affymetrix microarray)
ρ = 0.8
(p=0.0041)
0
2
4
6
8
10
12
4
6
8
10
12
14

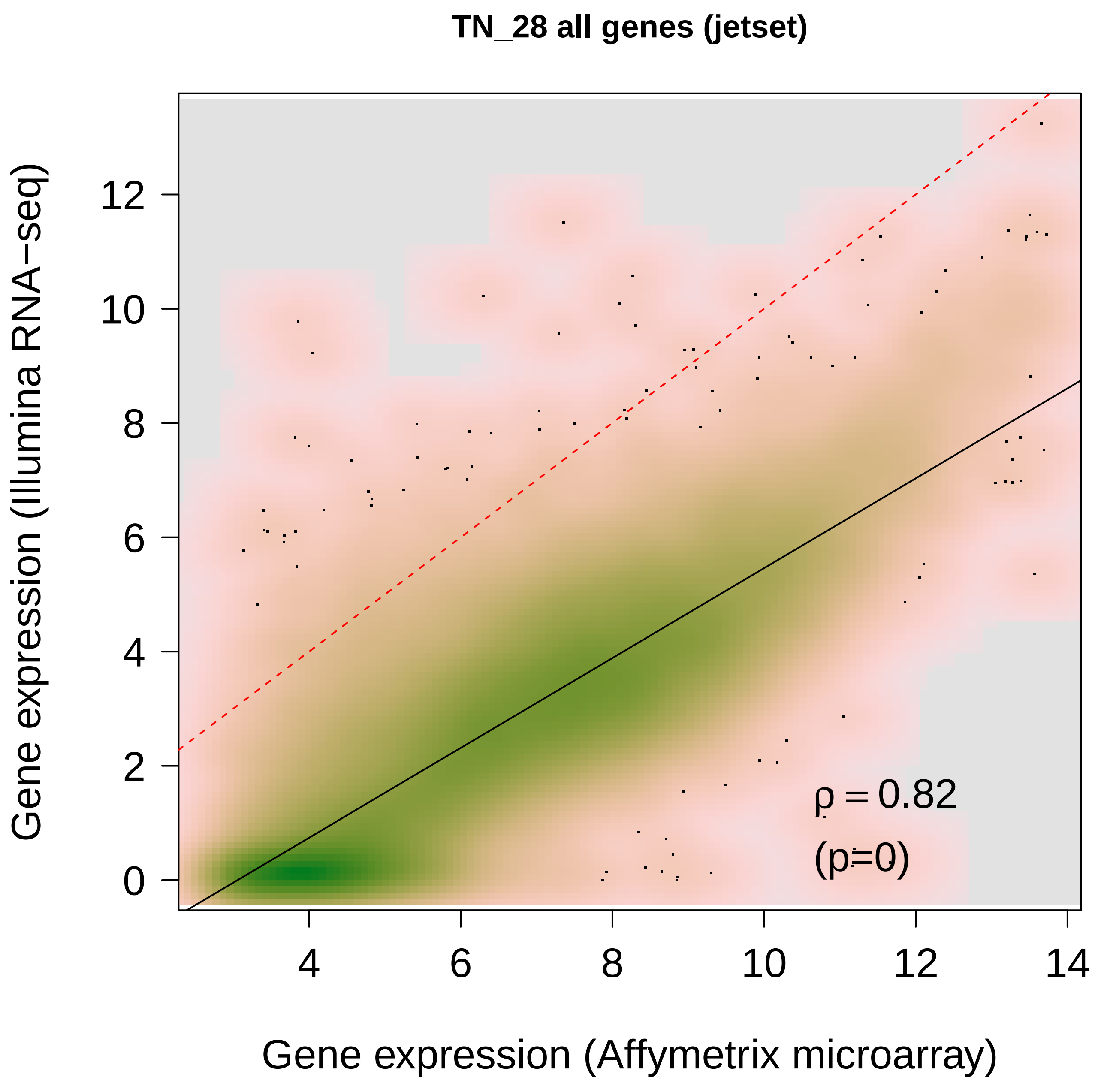
TN_28 all genes (jetset)
Gene expression (Illumina RNA−seq)
Gene expression (Affymetrix microarray)
ρ = 0.82
(p=0)
0
2
4
6
8
10
12
4
6
8
10
12
14

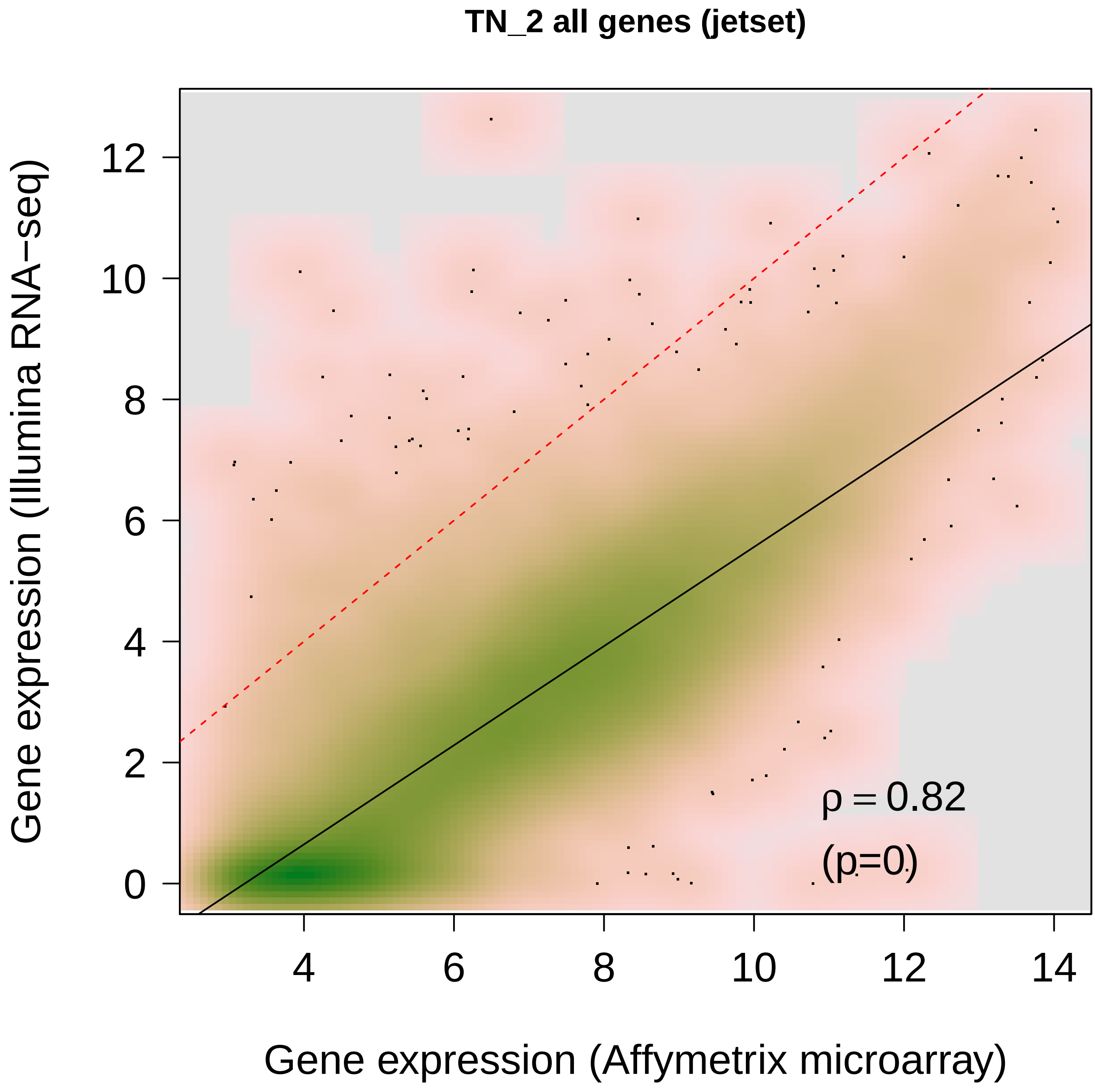
TN_2 all genes (jetset)
ρ = 0.82
(p=0)
Gene expression (Illumina RNA−seq)
Gene expression (Affymetrix microarray)
0
2
4
6
8
10
12
4
6
8
10
12
14

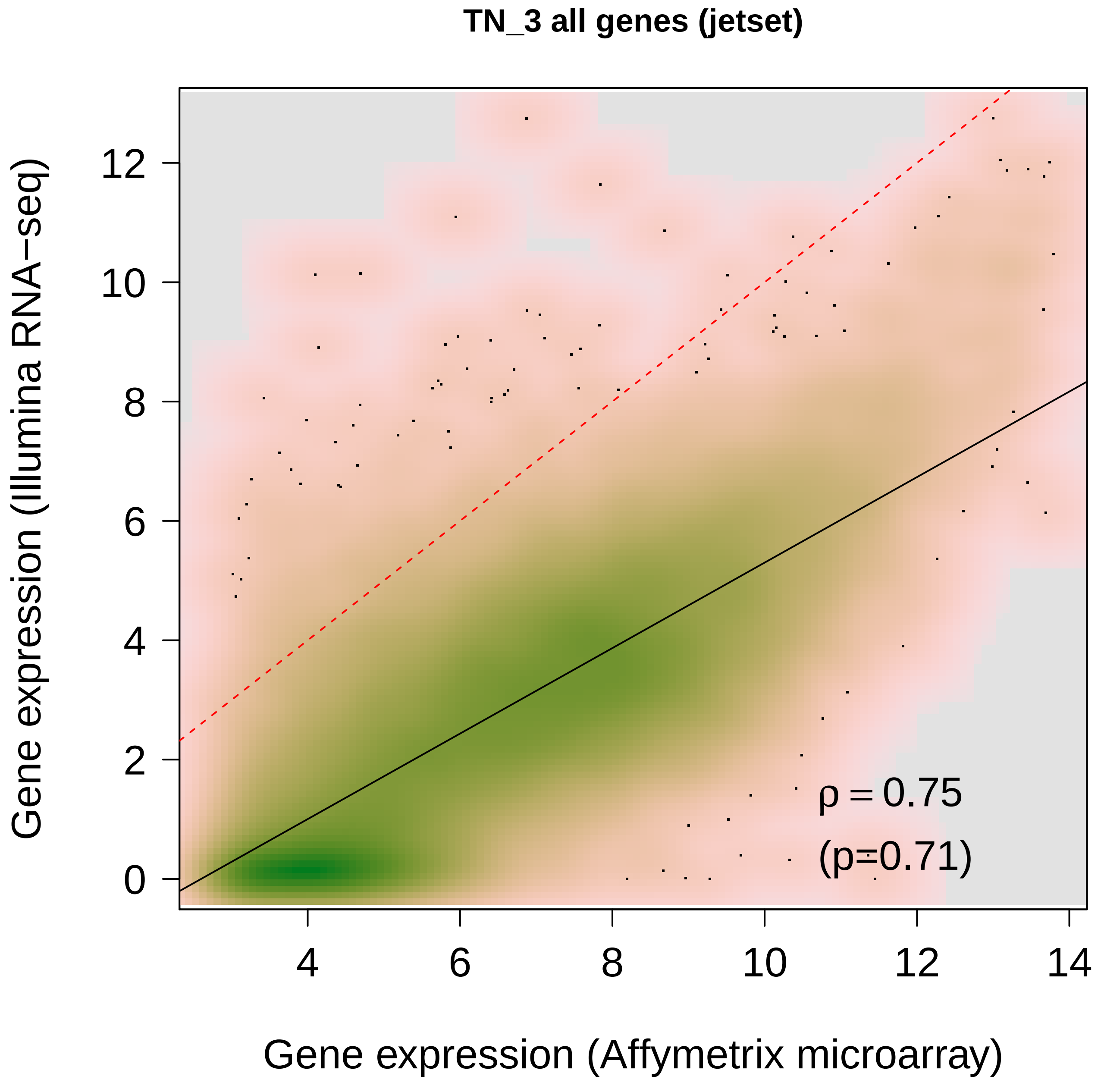
TN_3 all genes (jetset)
Gene expression (Illumina RNA−seq)
Gene expression (Affymetrix microarray)
0
2
4
6
8
10
12
4
6
8
10
12
14
ρ = 0.75
(p=0.71)

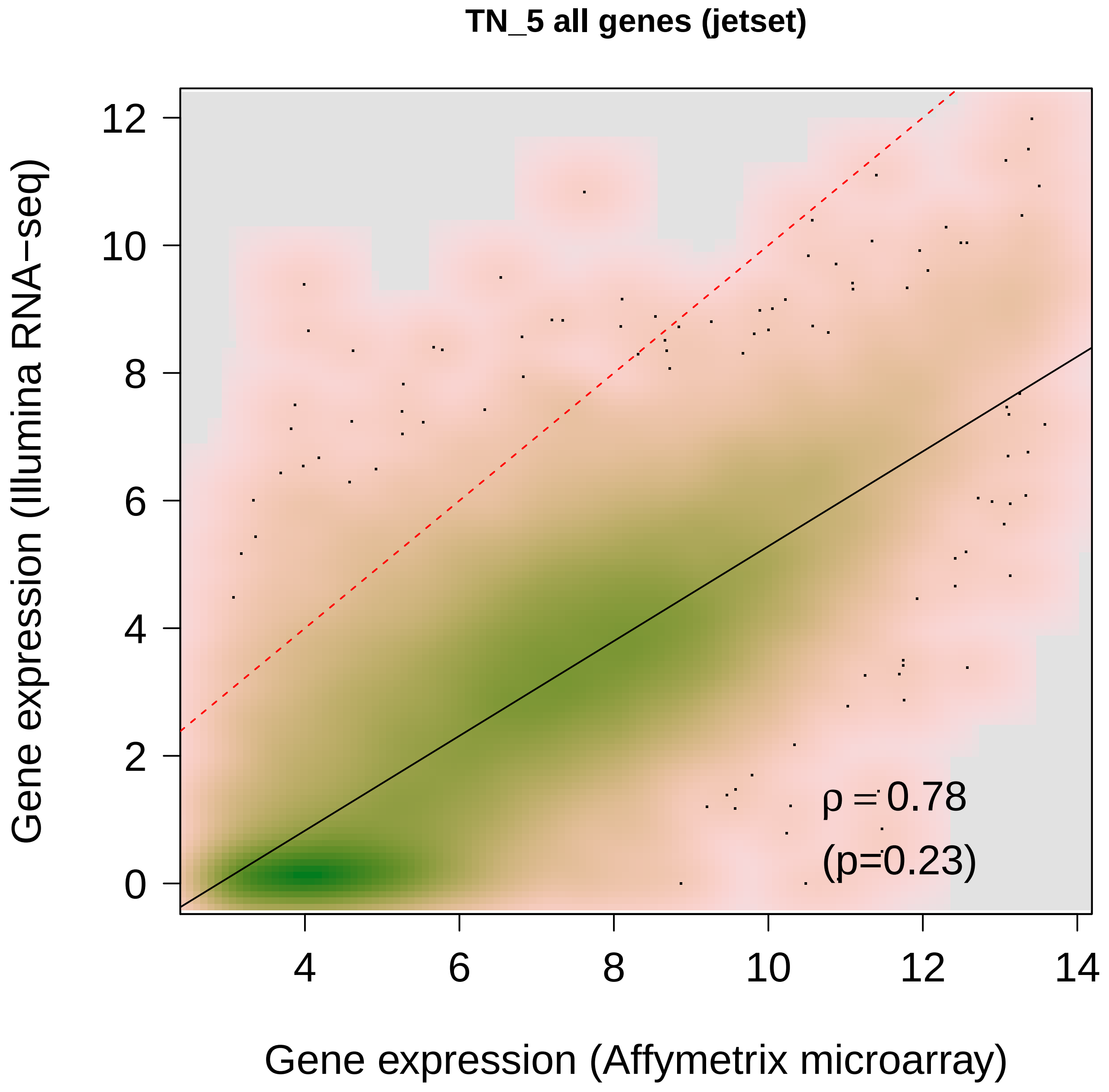

TN_5 all genes (jetset)
Gene expression (Illumina RNA−seq)
Gene expression (Affymetrix microarray)
ρ = 0.78
(p=0.23)
0
2
4
6
8
10
12
4
6
8
10
12
14
