# Supplementary figures and images for: Transfer of clinically relevant gene expression signatures in breast cancer: from Affymetrix microarray to Illumina RNA-Sequencing technology
Source: BMC Genomics. 2014 Nov 21;15(1):1008. doi: 10.1186/1471-2164-15-1008 (PMC4289354; doi:10.1186/1471-2164-15-1008)

ER

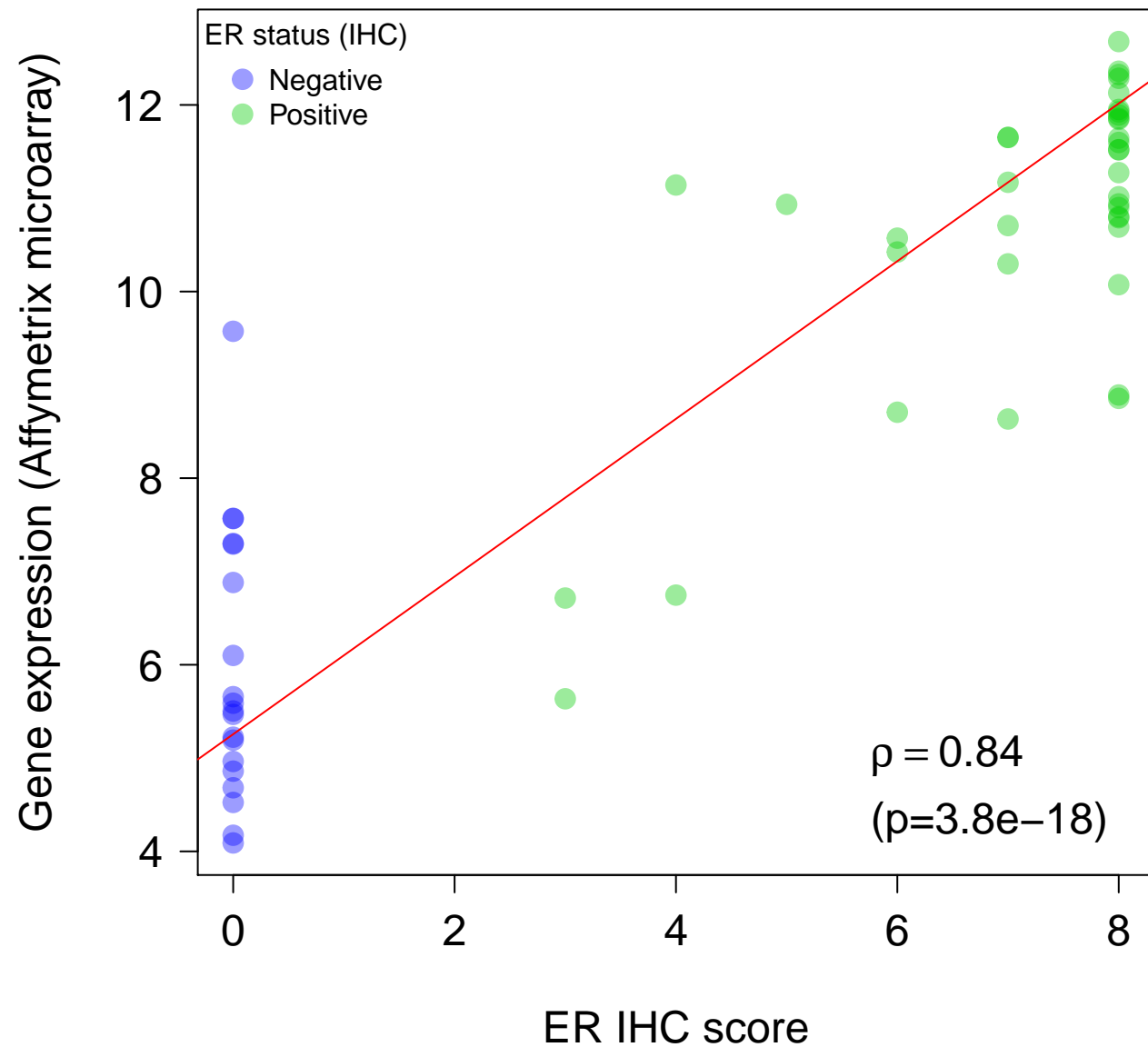

ER

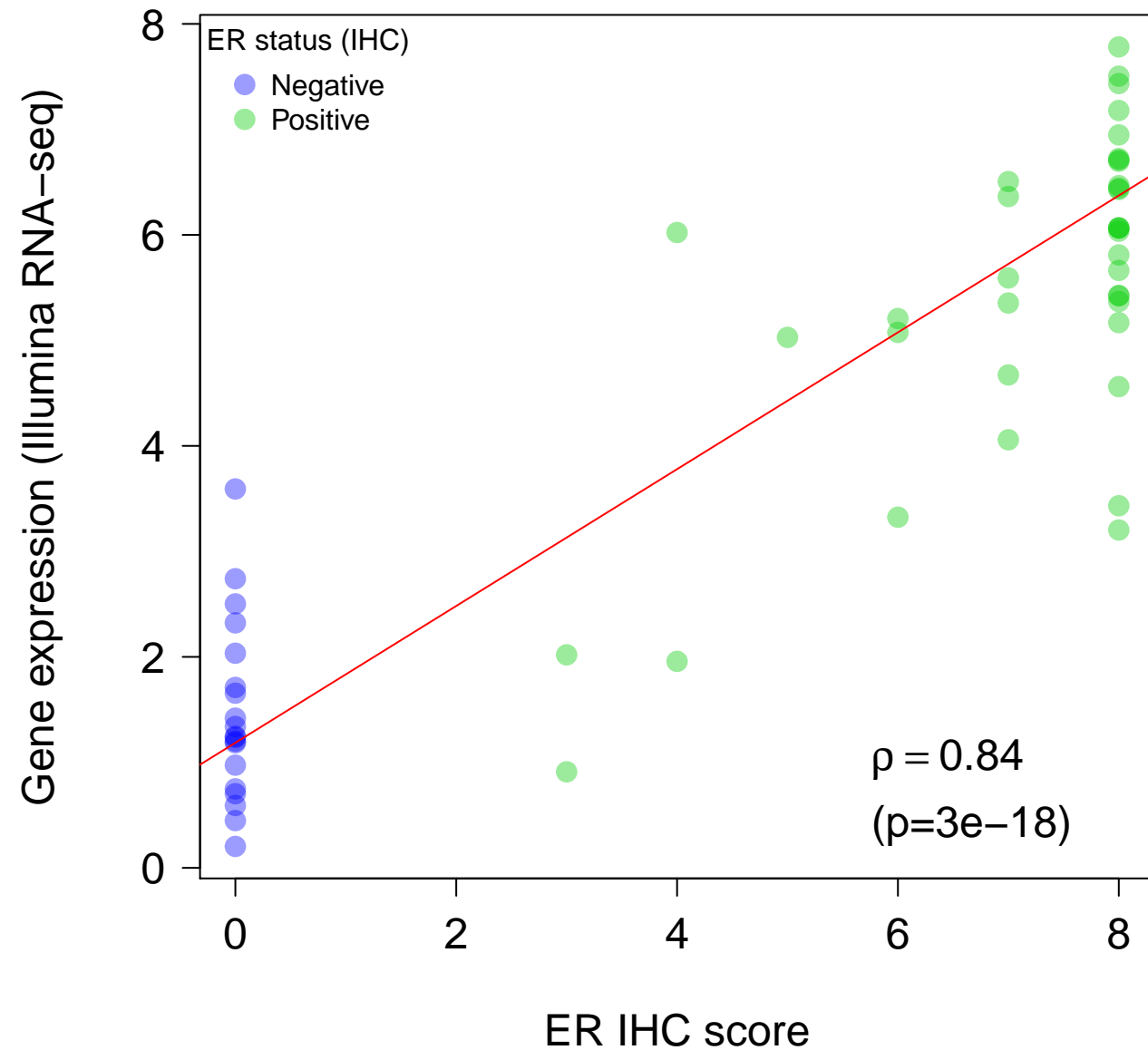

## PGR

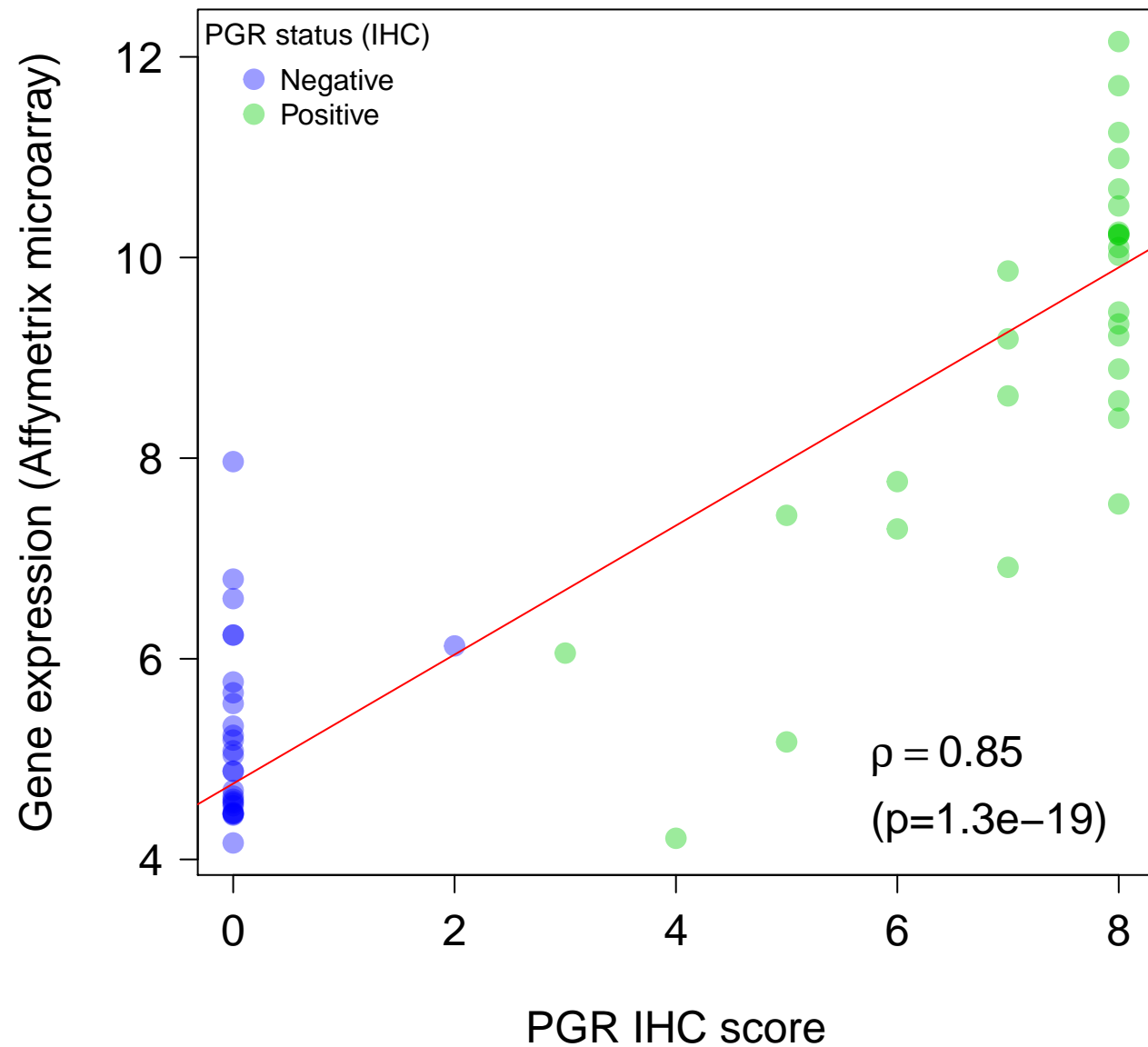

## PGR

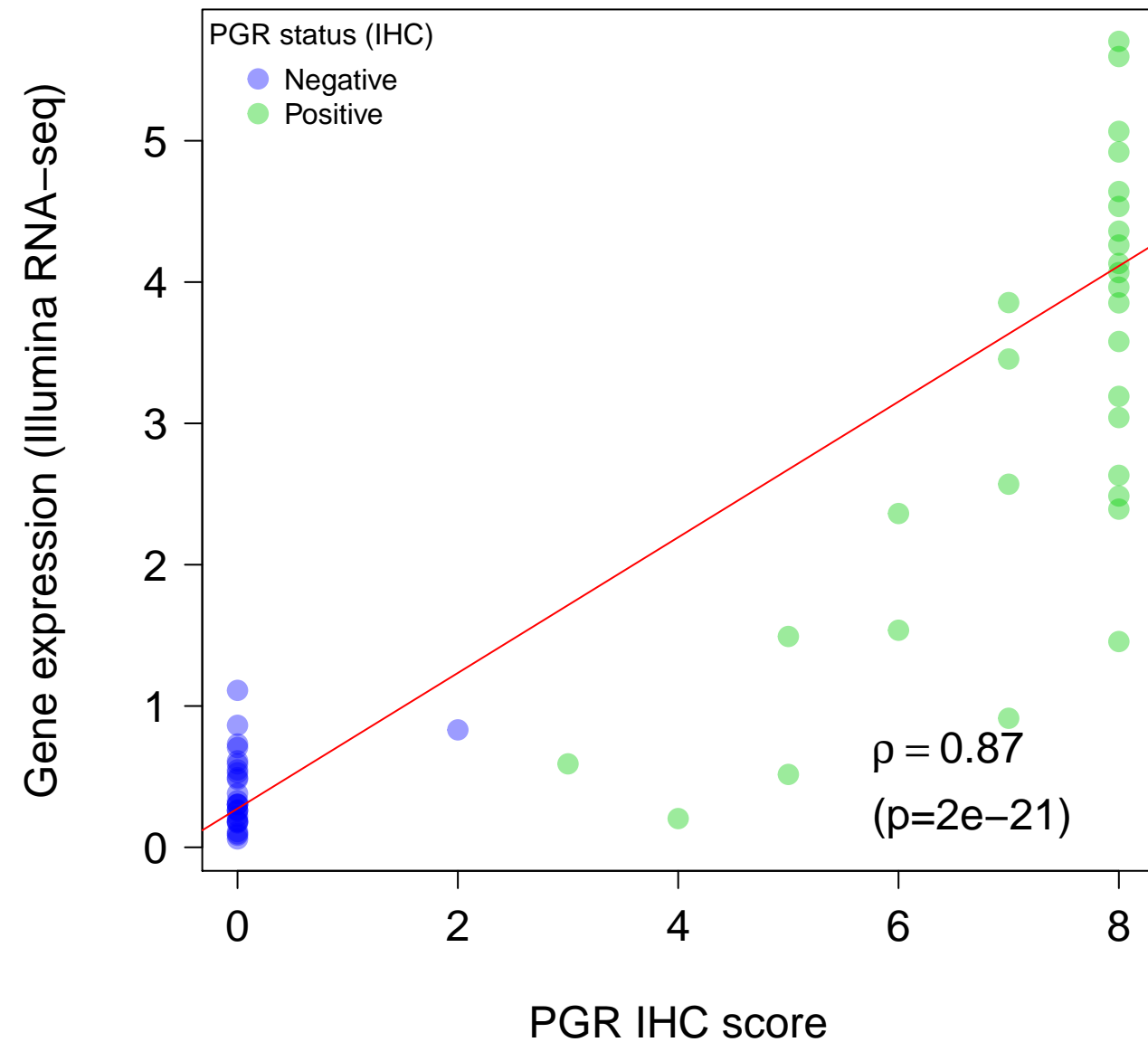

## HER2

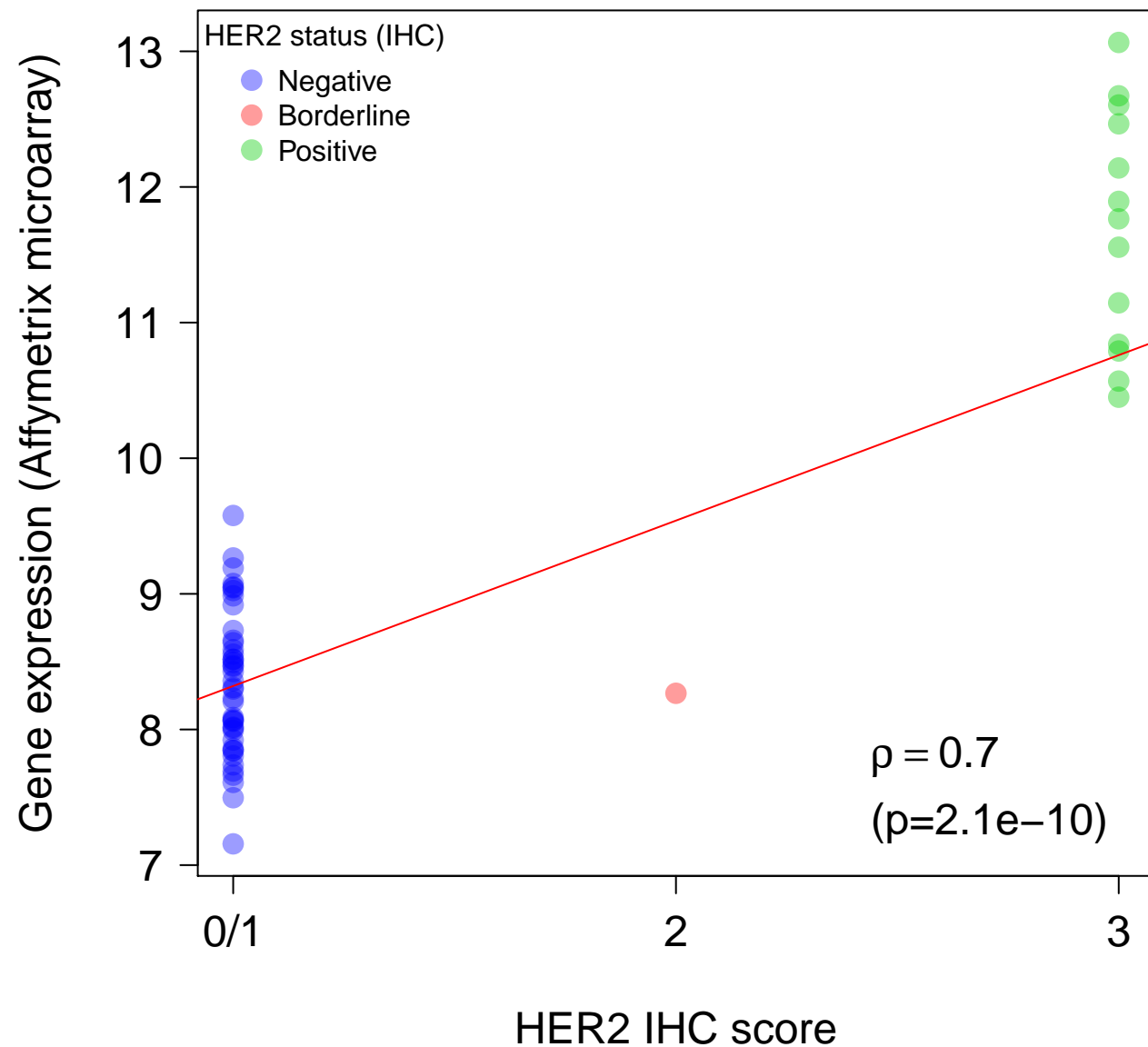

## HER2

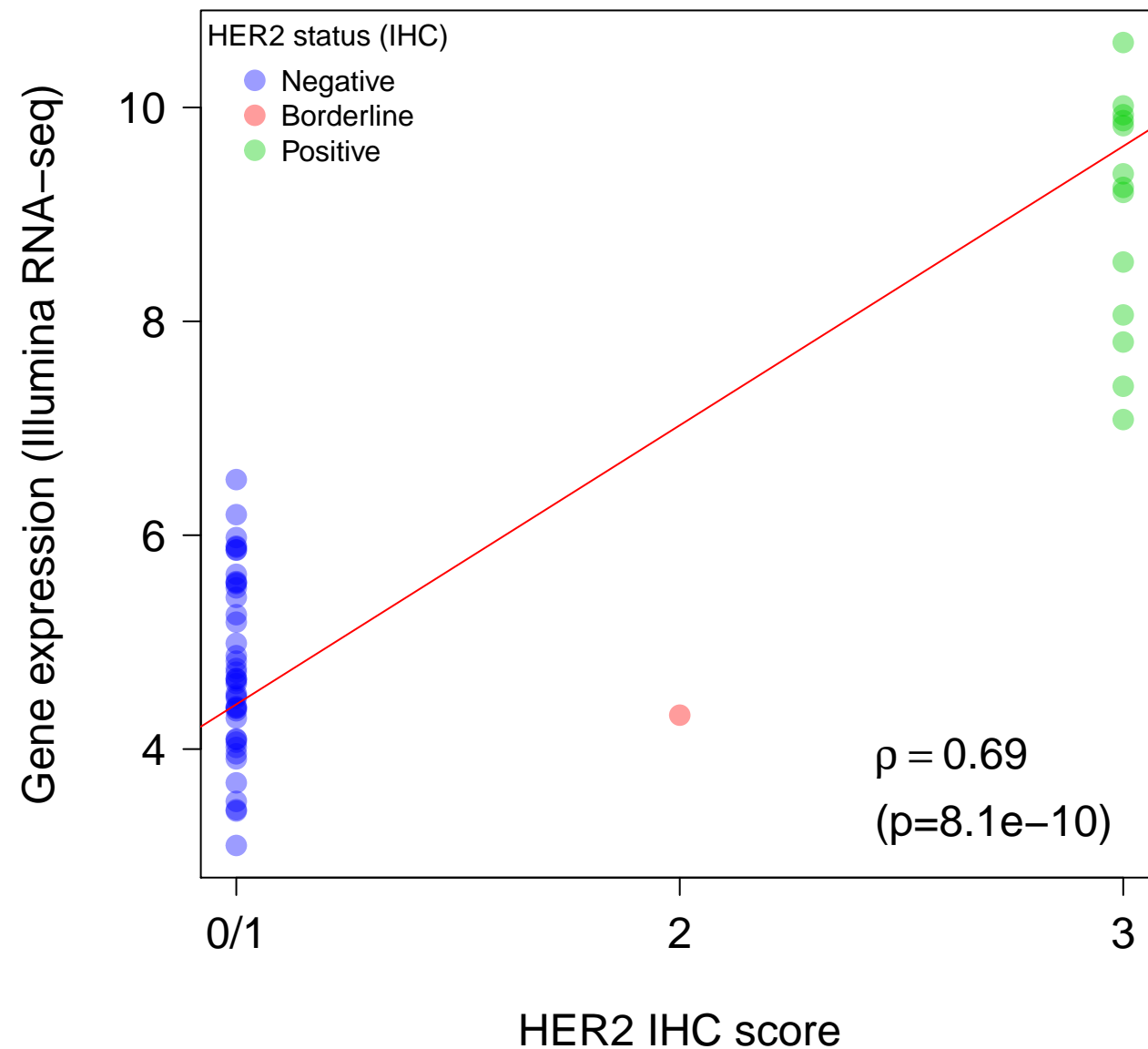

Supplement: Supplementary file 5 — Additional file 5: Figure S4: Spearman correlation for the quantification of three clinically relevant genes (ER, PgR, and HER2) as defined by IHC vs Affymetrix microarray and Illumina RNA-Seq, respectively. (PDF 37 KB) [file 12864_2014_6829_MOESM5_ESM.pdf]

## All signature genes combined

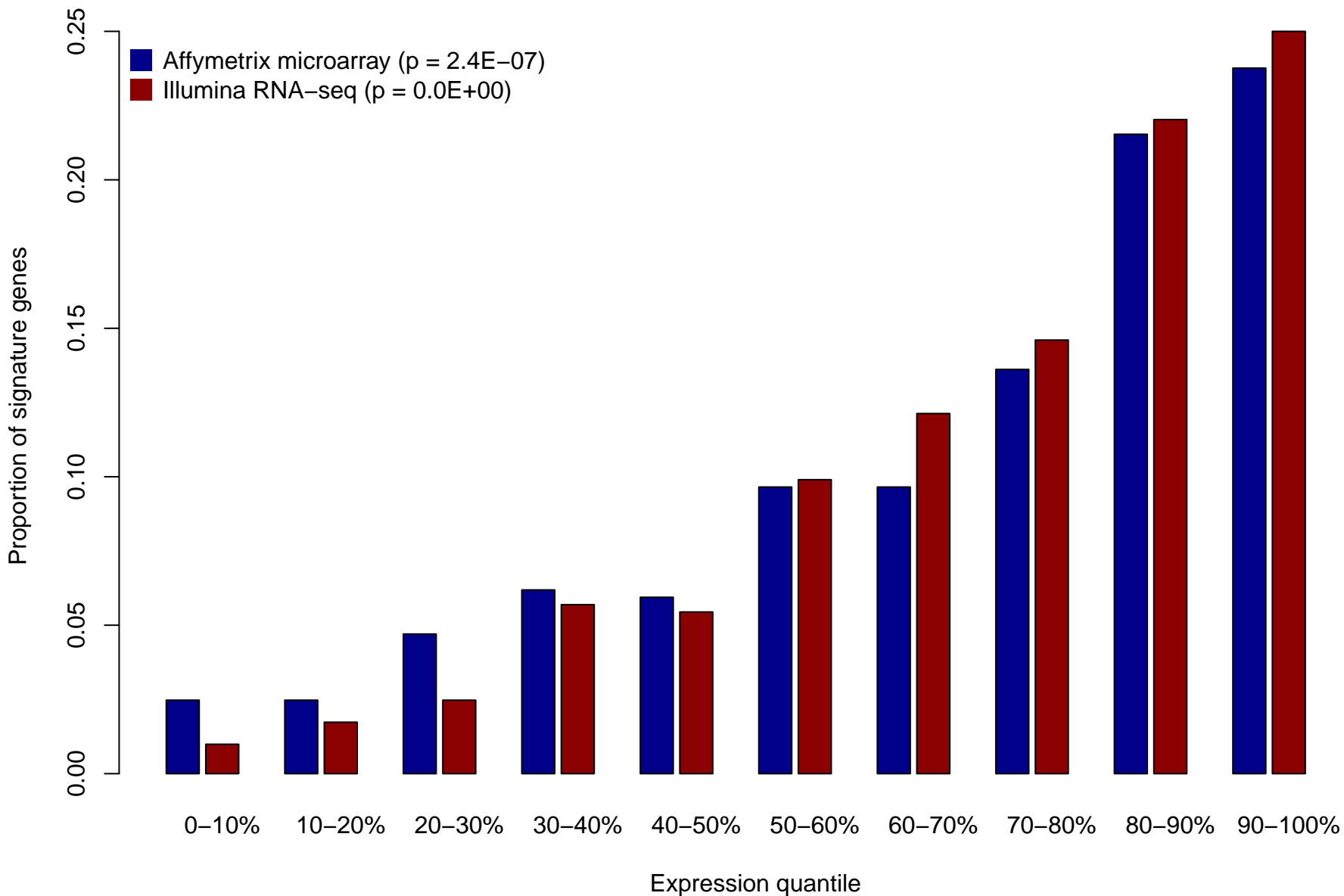

Supplement: Supplementary file 6 — Additional file 6: Figure S5: Bar plots representing the proportion of genes present in all signatures combined (3,663 unique genes in 27 signatures) with respect to their quantiles of expression for Affymetrix microarray (blue) and Illumina RNA-seq (red) platforms. The p-value reports the significance of the enrichment of signature genes with increasing quantiles of expression (Spearman’s rank-based correlation). (PDF 9 KB) [file 12864_2014_6829_MOESM6_ESM.pdf]
