# Supplementary material for: Transfer of clinically relevant gene expression signatures in breast cancer: from Affymetrix microarray to Illumina RNA-Sequencing technology
Source: BMC Genomics. 2014 Nov 21;15(1):1008. doi: 10.1186/1471-2164-15-1008 (PMC4289354; doi:10.1186/1471-2164-15-1008)
Supplement: Supplementary file 7 — Additional file 7: Figure S6: Bar plots representing, for each signature (27), the proportion of genes present in all signatures combined with respect to their quantiles of expression for Affymetrix microarray (blue) and Illumina RNA-seq (red) platforms. The p-value reports the significance of the enrichment of signature genes with increasing quantiles of expression (Spearman’s rank-based correlation). Note that the SCMGENE and IRMODULE signatures contains few genes (3 and 6 genes, respectively) while the median signature size is 95 genes; for these small signatures, the p-value is expected to be large due to reduce sample size for the correlation analysis. (PDF 36 KB) [file 12864_2014_6829_MOESM7_ESM.pdf]

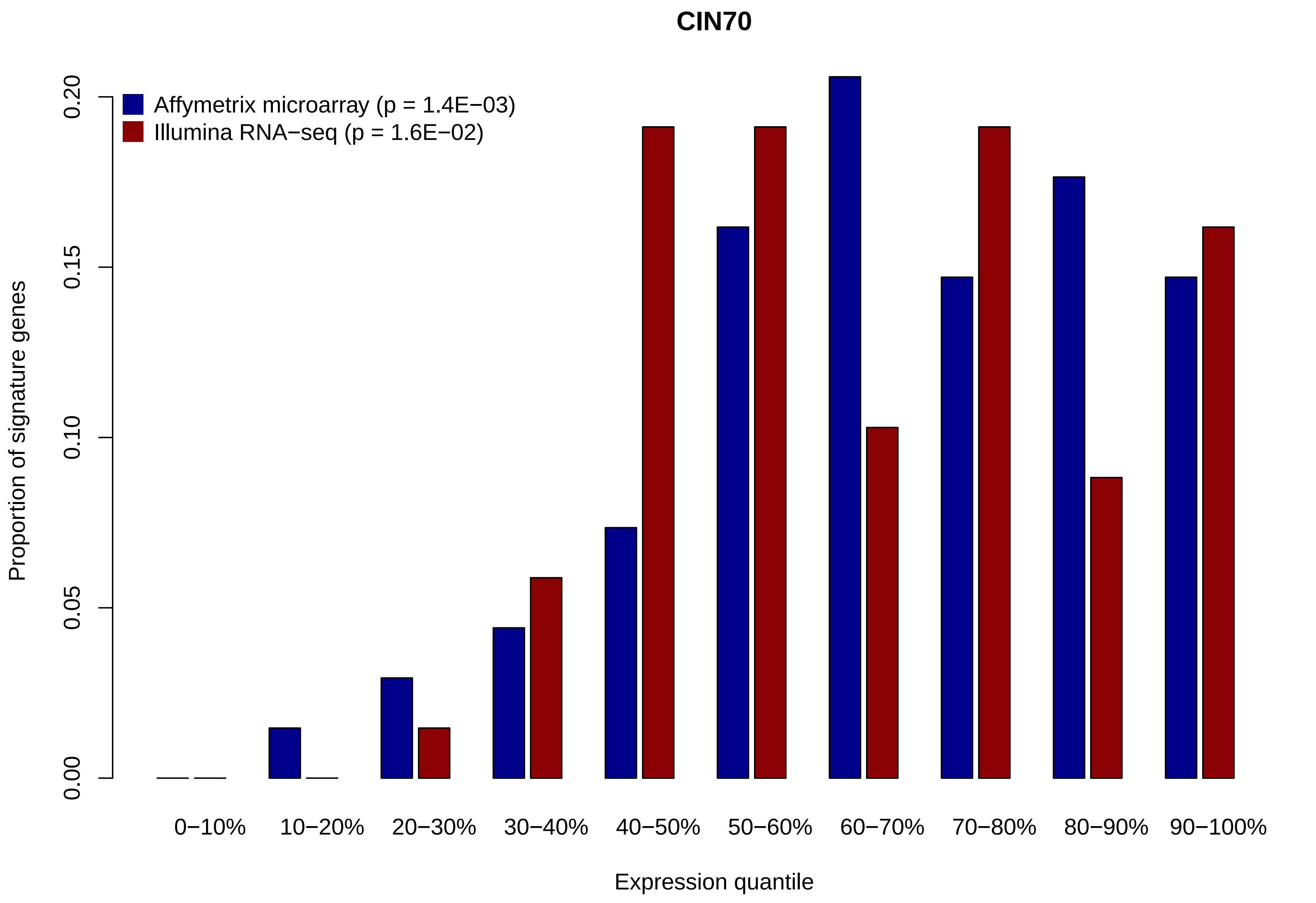
CIN70
Affymetrix microarray (p = 1.4E−03)
Illumina RNA−seq (p = 1.6E−02)
Proportion of signature genes
0.00
0.05
0.10
0.15
0.20
0−10%
10−20%
20−30%
30−40%
40−50%
50−60%
60−70%
70−80%
80−90%
90−100%
Expression quantile

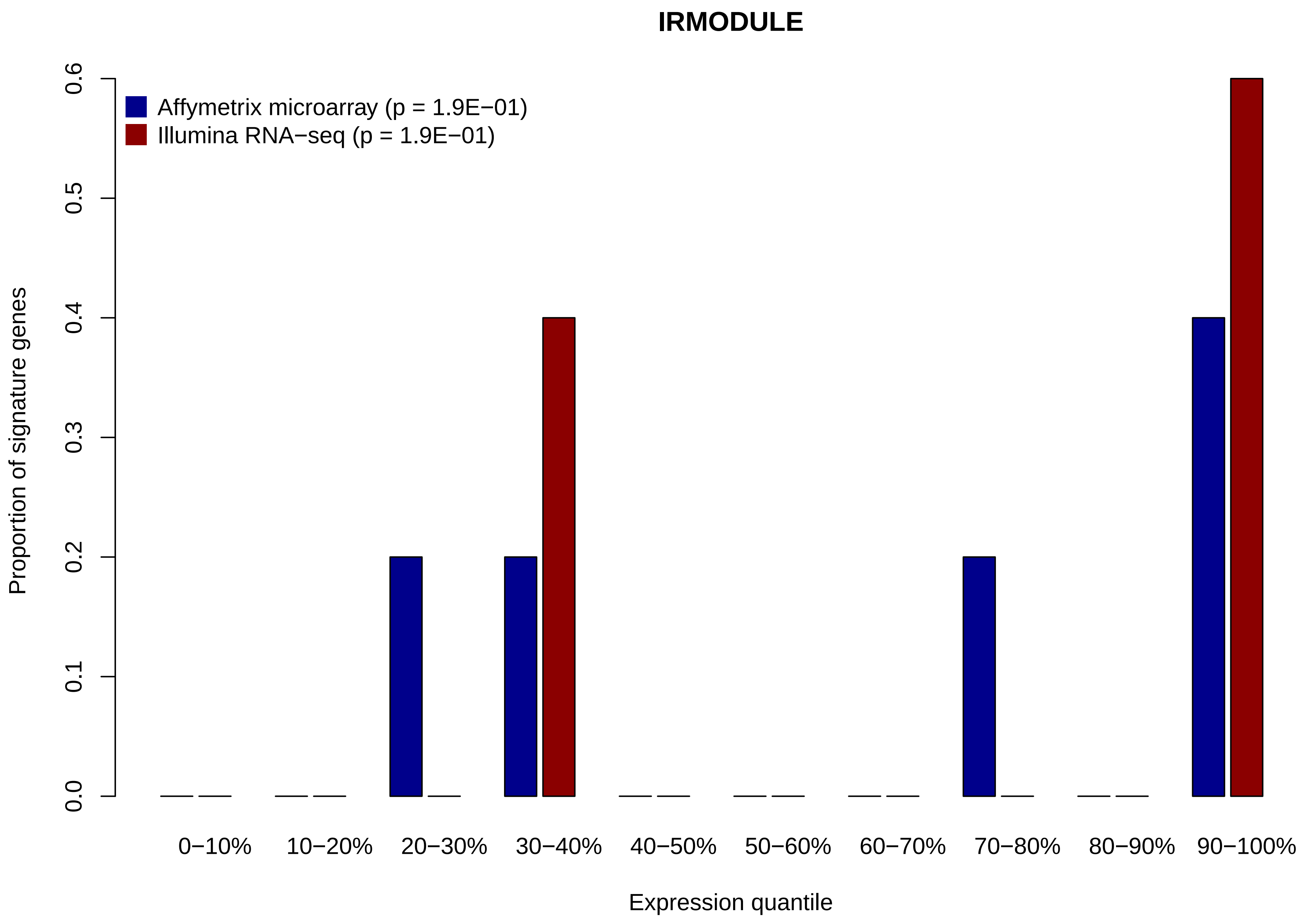
IRMODULE
Affymetrix microarray (p = 1.9E−01)
Illumina RNA−seq (p = 1.9E−01)
Proportion of signature genes
0.0
0.1
0.2
0.3
0.4
0.5
0.6
0−10%
10−20%
20−30%
30−40%
40−50%
50−60%
60−70%
70−80%
80−90%
90−100%
Expression quantile

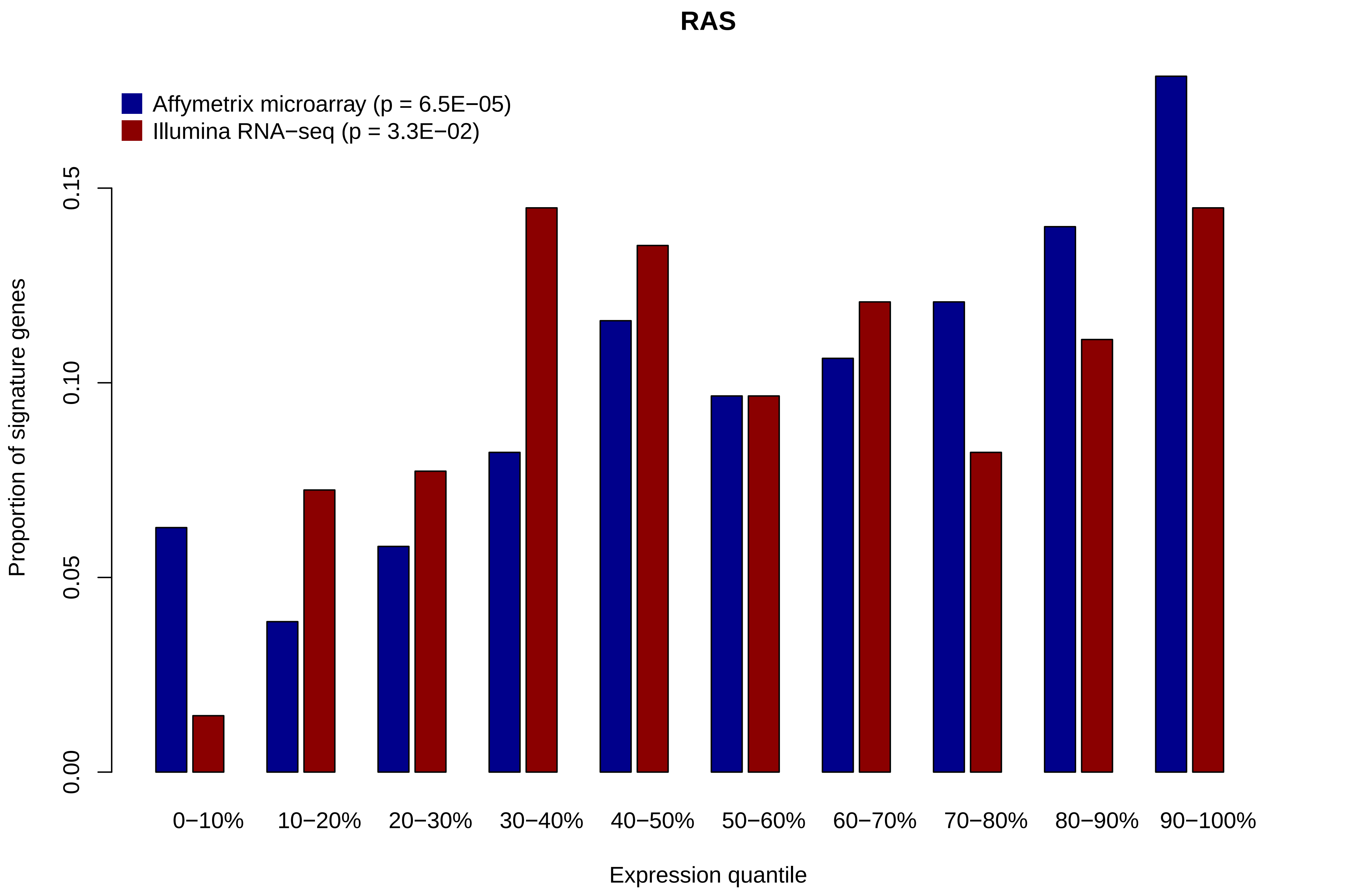

RAS
Affymetrix microarray (p = 6.5E−05)
Illumina RNA−seq (p = 3.3E−02)
Proportion of signature genes
0.00
0.05
0.10
0.15
0−10%
10−20%
20−30%
30−40%
40−50%
50−60%
60−70%
70−80%
80−90%
90−100%
Expression quantile

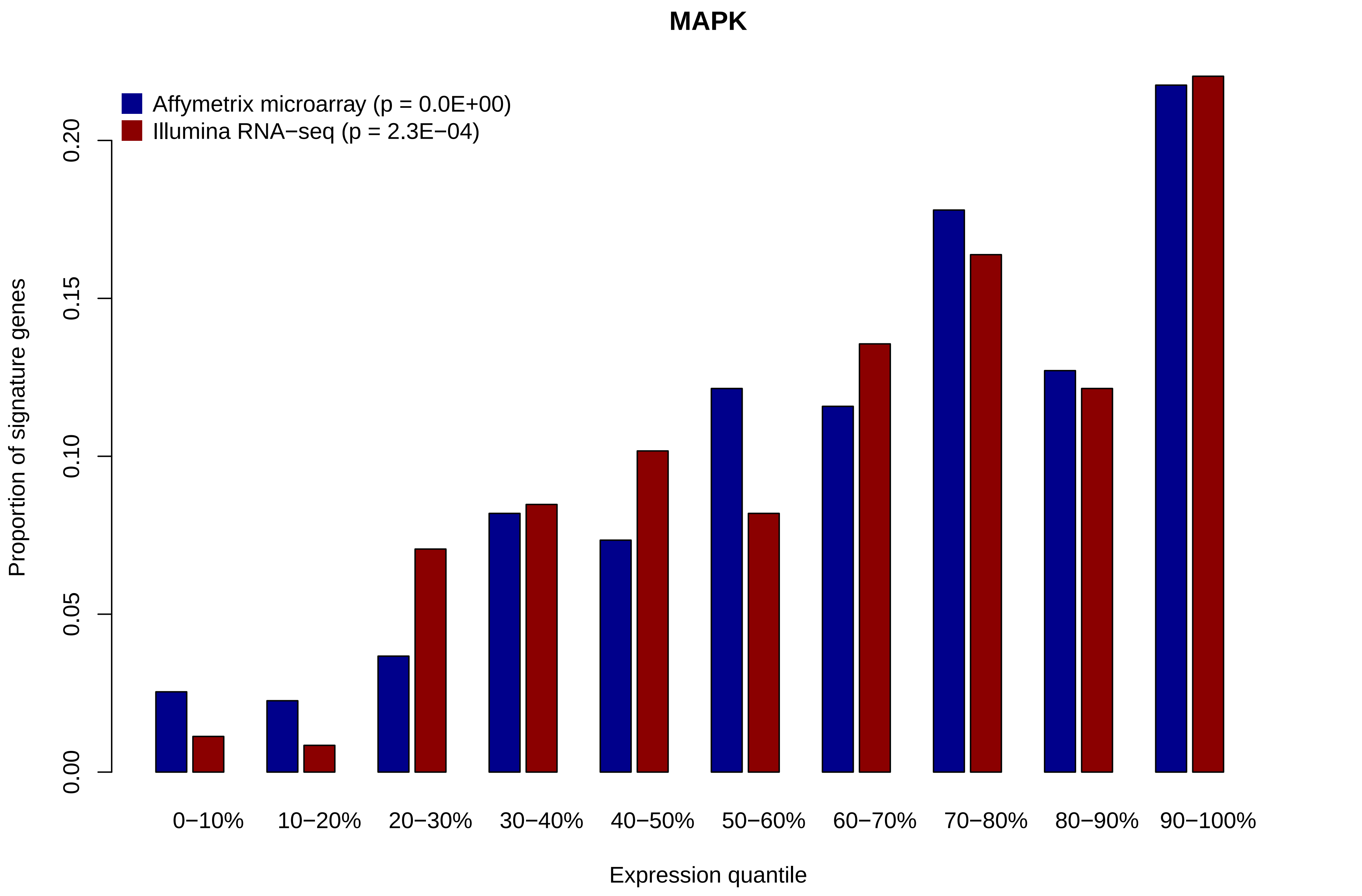
MAPK
Affymetrix microarray (p = 0.0E+00)
Illumina RNA−seq (p = 2.3E−04)
Proportion of signature genes
0.00
0.05
0.10
0.15
0.20
0−10%
10−20%
20−30%
30−40%
40−50%
50−60%
60−70%
70−80%
80−90%
90−100%
Expression quantile

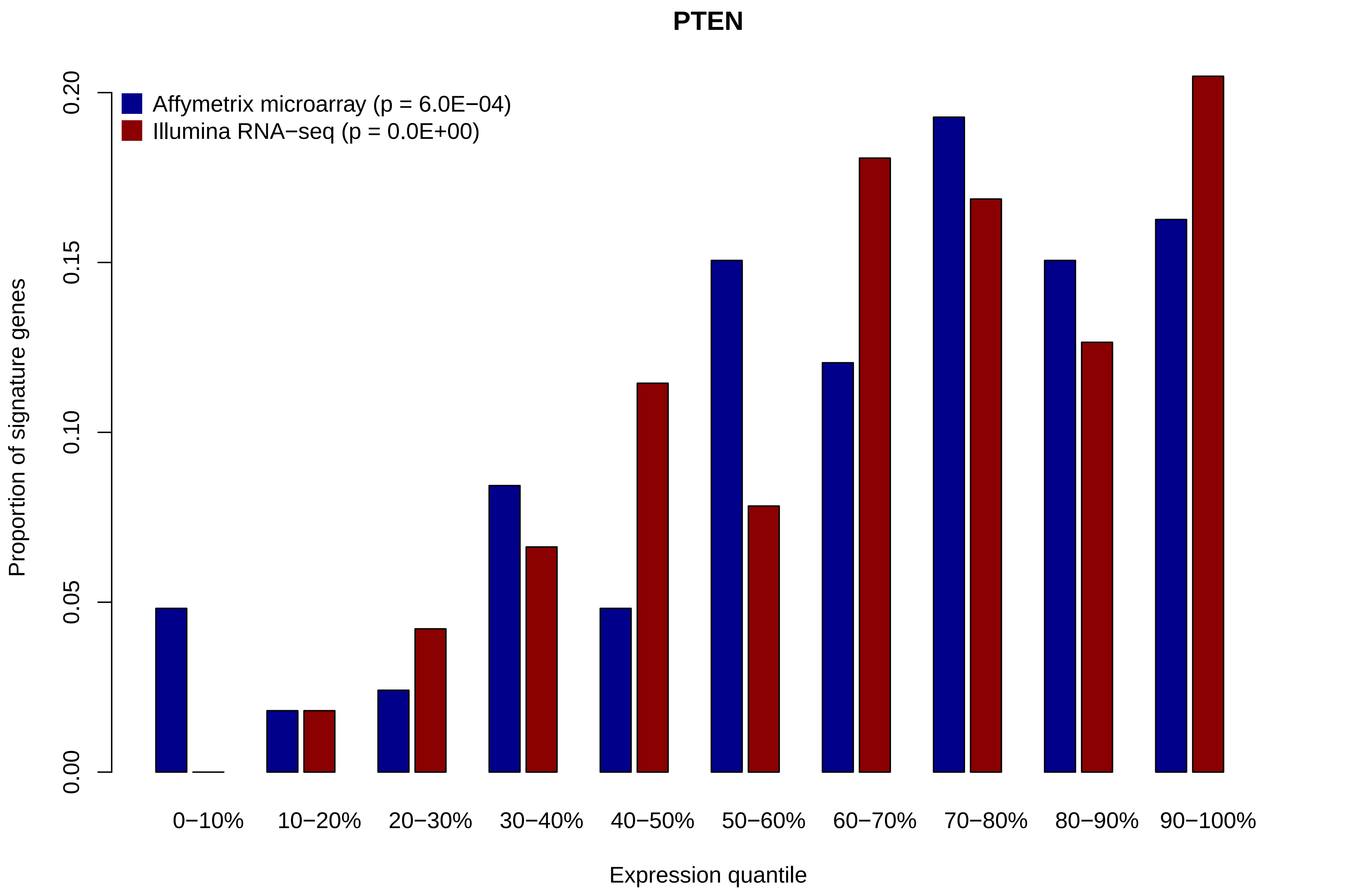
PTEN
Affymetrix microarray (p = 6.0E−04)
Illumina RNA−seq (p = 0.0E+00)
Proportion of signature genes
0.00
0.05
0.10
0.15
0.20
0−10%
10−20%
20−30%
30−40%
40−50%
50−60%
60−70%
70−80%
80−90%
90−100%
Expression quantile

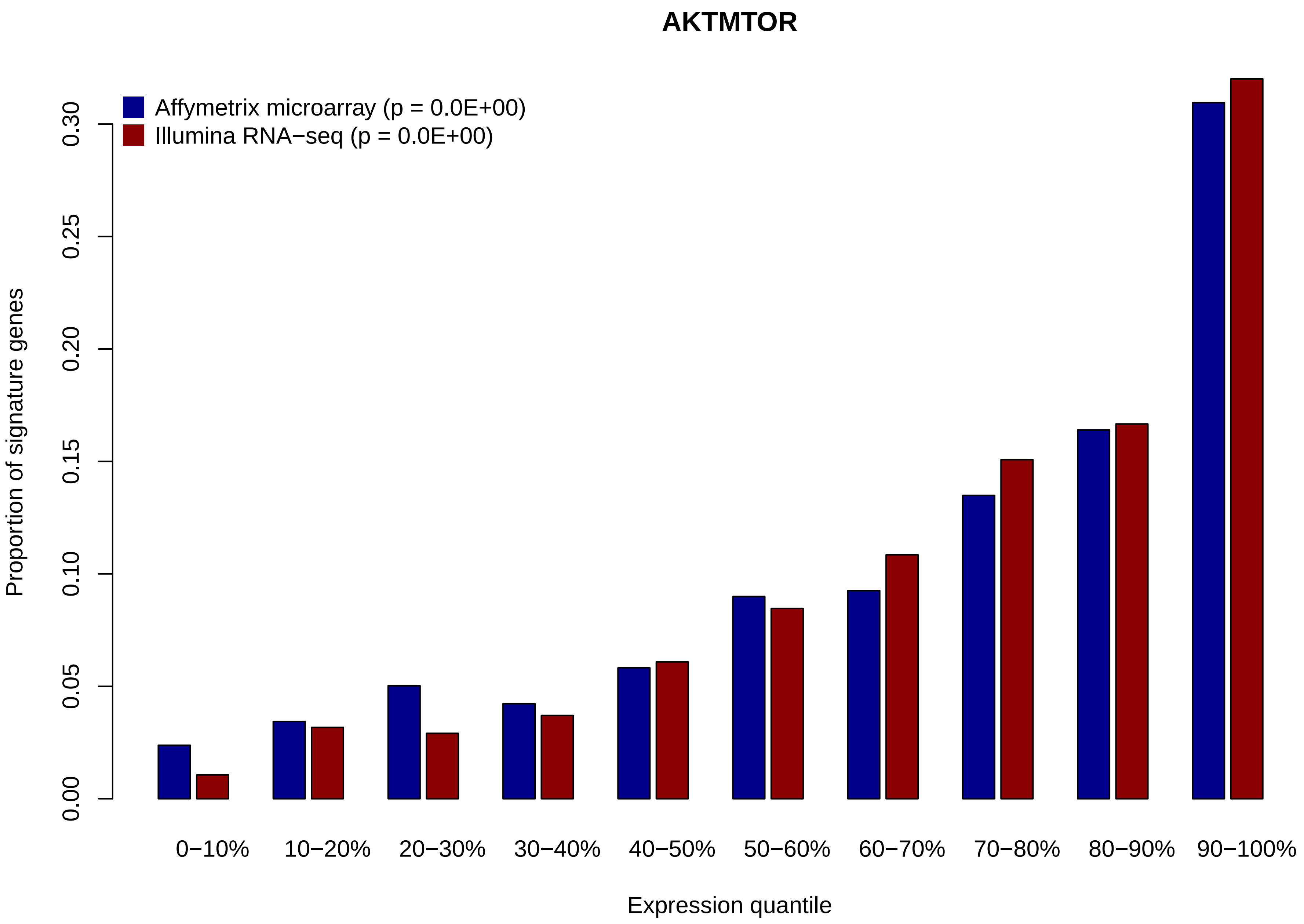

AKTMTOR
Affymetrix microarray (p = 0.0E+00)
Illumina RNA−seq (p = 0.0E+00)
Proportion of signature genes
0.00
0.05
0.10
0.15
0.20
0.25
0.30
0−10%
10−20%
20−30%
30−40%
40−50%
50−60%
60−70%
70−80%
80−90%
90−100%
Expression quantile

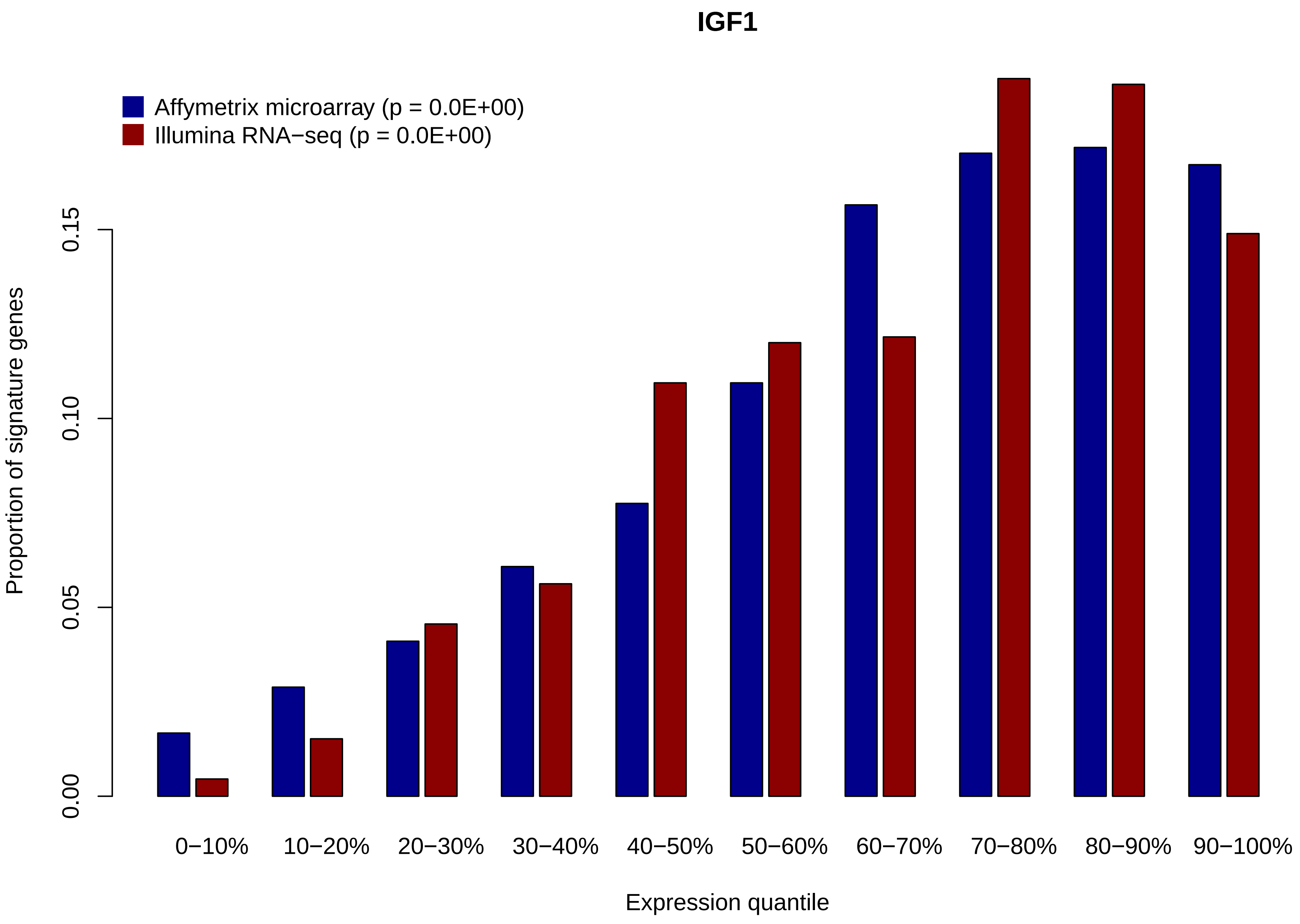
IGF1
Affymetrix microarray (p = 0.0E+00)
Illumina RNA−seq (p = 0.0E+00)
Proportion of signature genes
0.00
0.05
0.10
0.15
0−10%
10−20%
20−30%
30−40%
40−50%
50−60%
60−70%
70−80%
80−90%
90−100%
Expression quantile

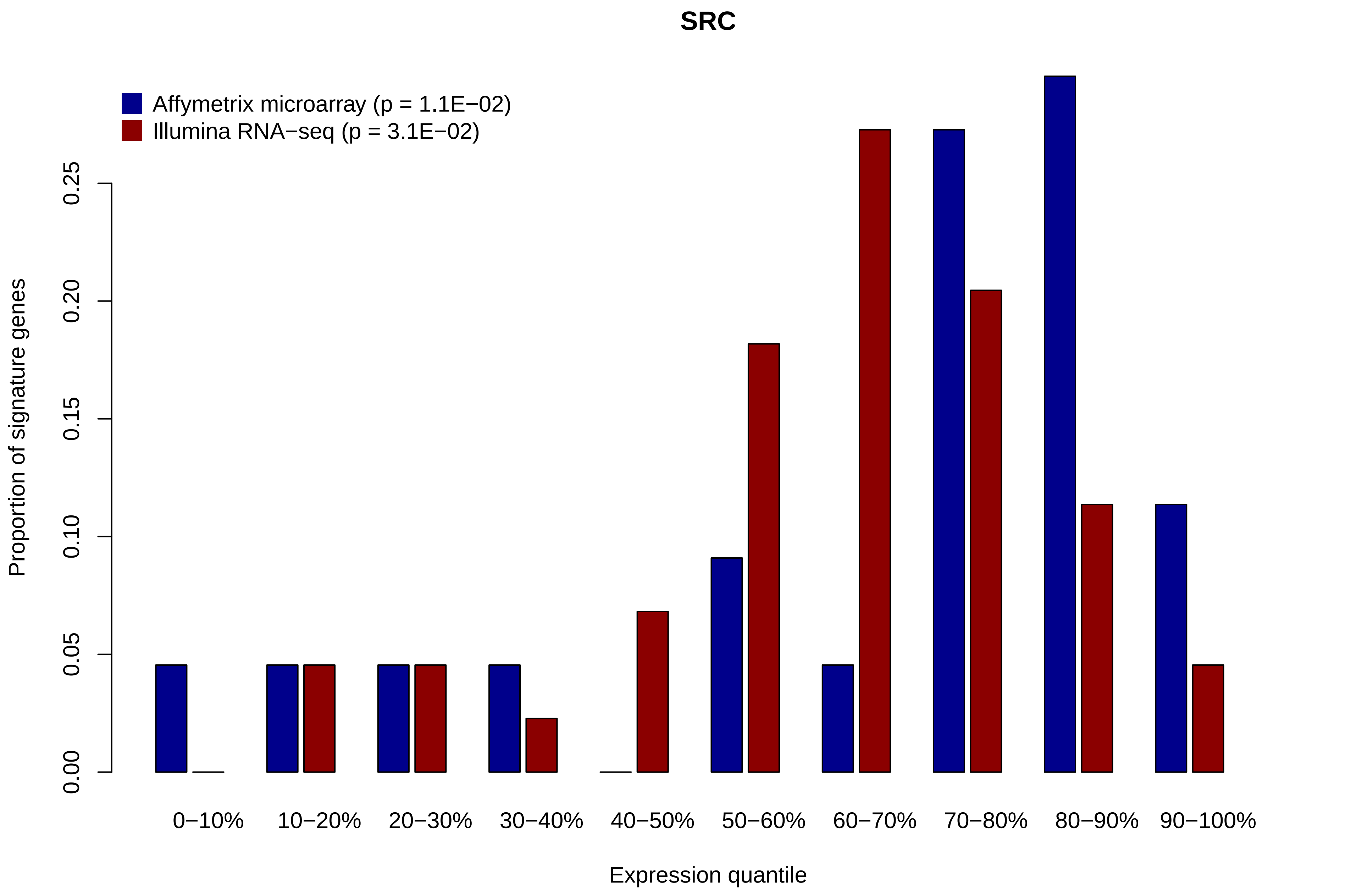
SRC
Affymetrix microarray (p = 1.1E−02)
Illumina RNA−seq (p = 3.1E−02)
Proportion of signature genes
0.00
0.05
0.10
0.15
0.20
0.25
0−10%
10−20%
20−30%
30−40%
40−50%
50−60%
60−70%
70−80%
80−90%
90−100%
Expression quantile

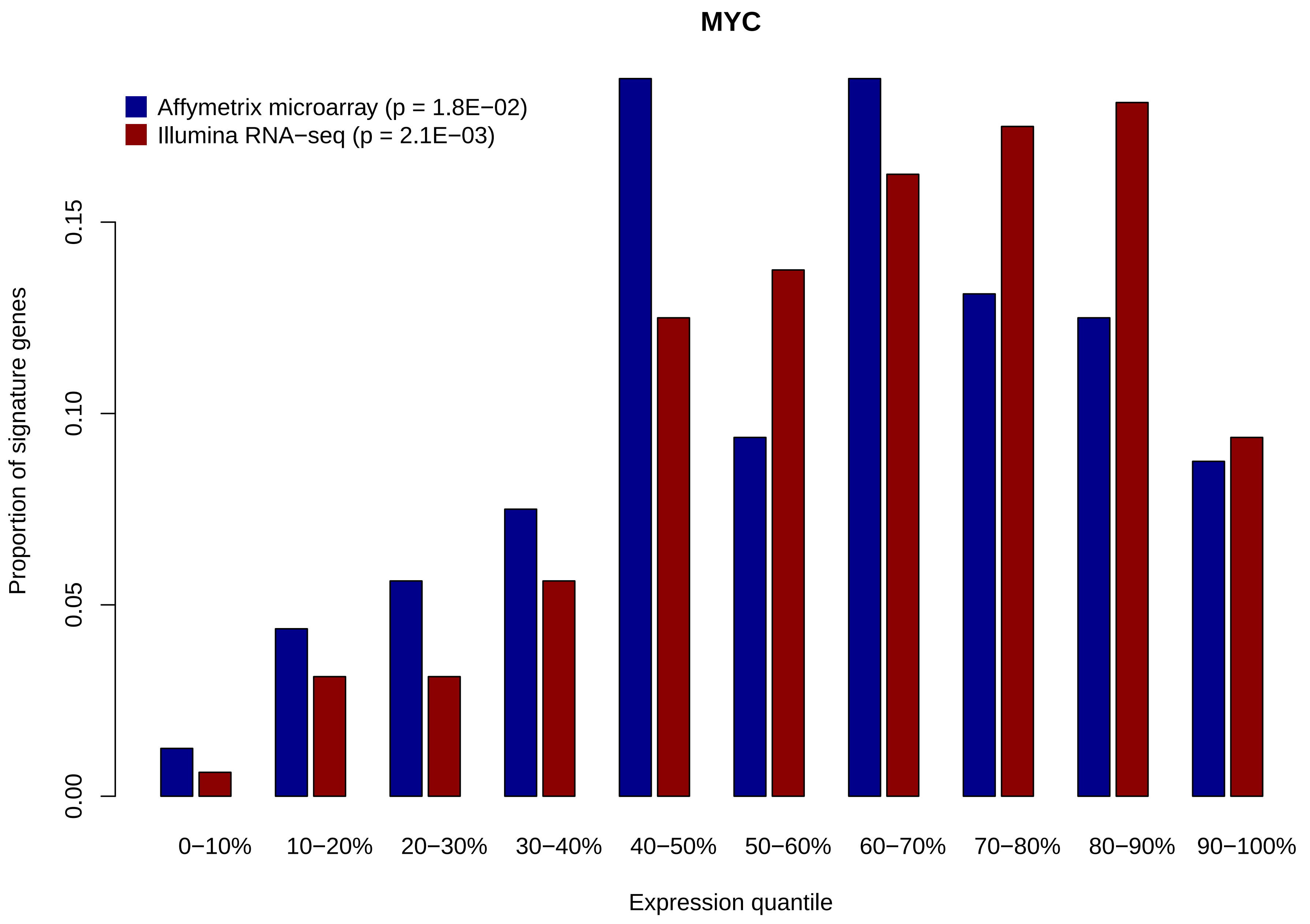

MYC
Affymetrix microarray (p = 1.8E−02)
Illumina RNA−seq (p = 2.1E−03)
Proportion of signature genes
0.00
0.05
0.10
0.15
0−10%
10−20%
20−30%
30−40%
40−50%
50−60%
60−70%
70−80%
80−90%
90−100%
Expression quantile

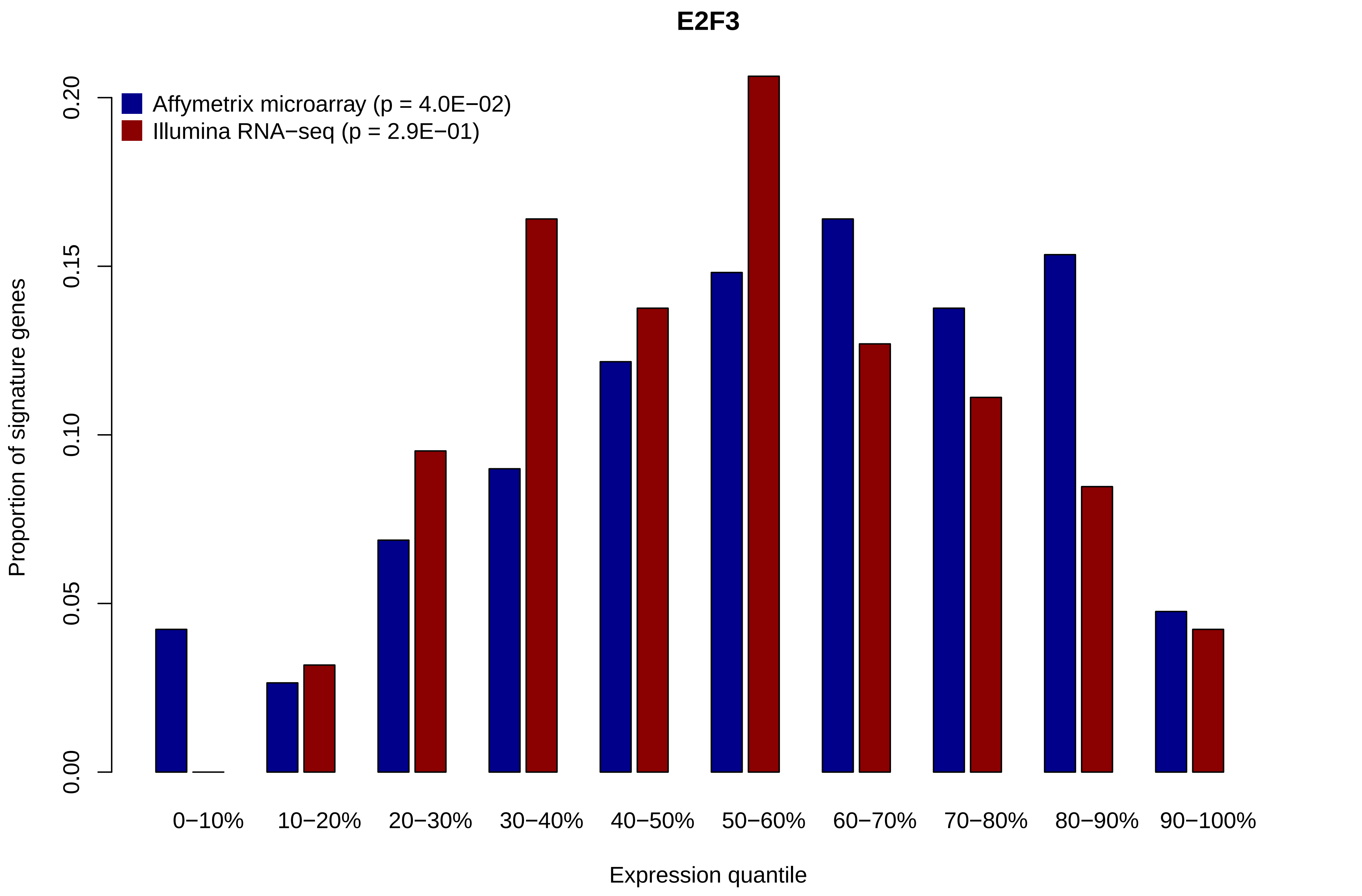
E2F3
Affymetrix microarray (p = 4.0E−02)
Illumina RNA−seq (p = 2.9E−01)
Proportion of signature genes
0.00
0.05
0.10
0.15
0.20
0−10%
10−20%
20−30%
30−40%
40−50%
50−60%
60−70%
70−80%
80−90%
90−100%
Expression quantile

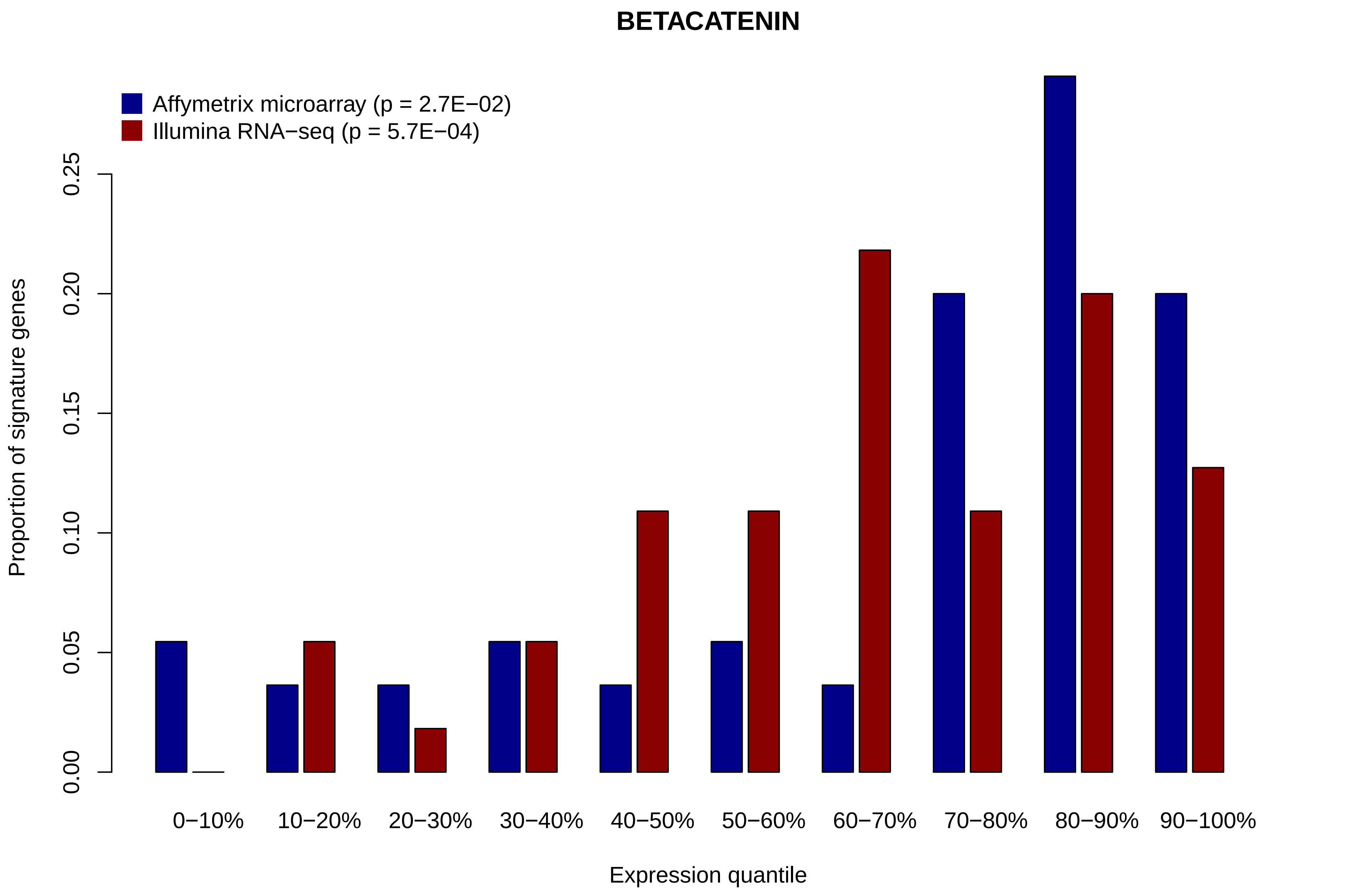
BETACATENIN
Affymetrix microarray (p = 2.7E−02)
Illumina RNA−seq (p = 5.7E−04)
Proportion of signature genes
0.00
0.05
0.10
0.15
0.20
0.25
0−10%
10−20%
20−30%
30−40%
40−50%
50−60%
60−70%
70−80%
80−90%
90−100%
Expression quantile

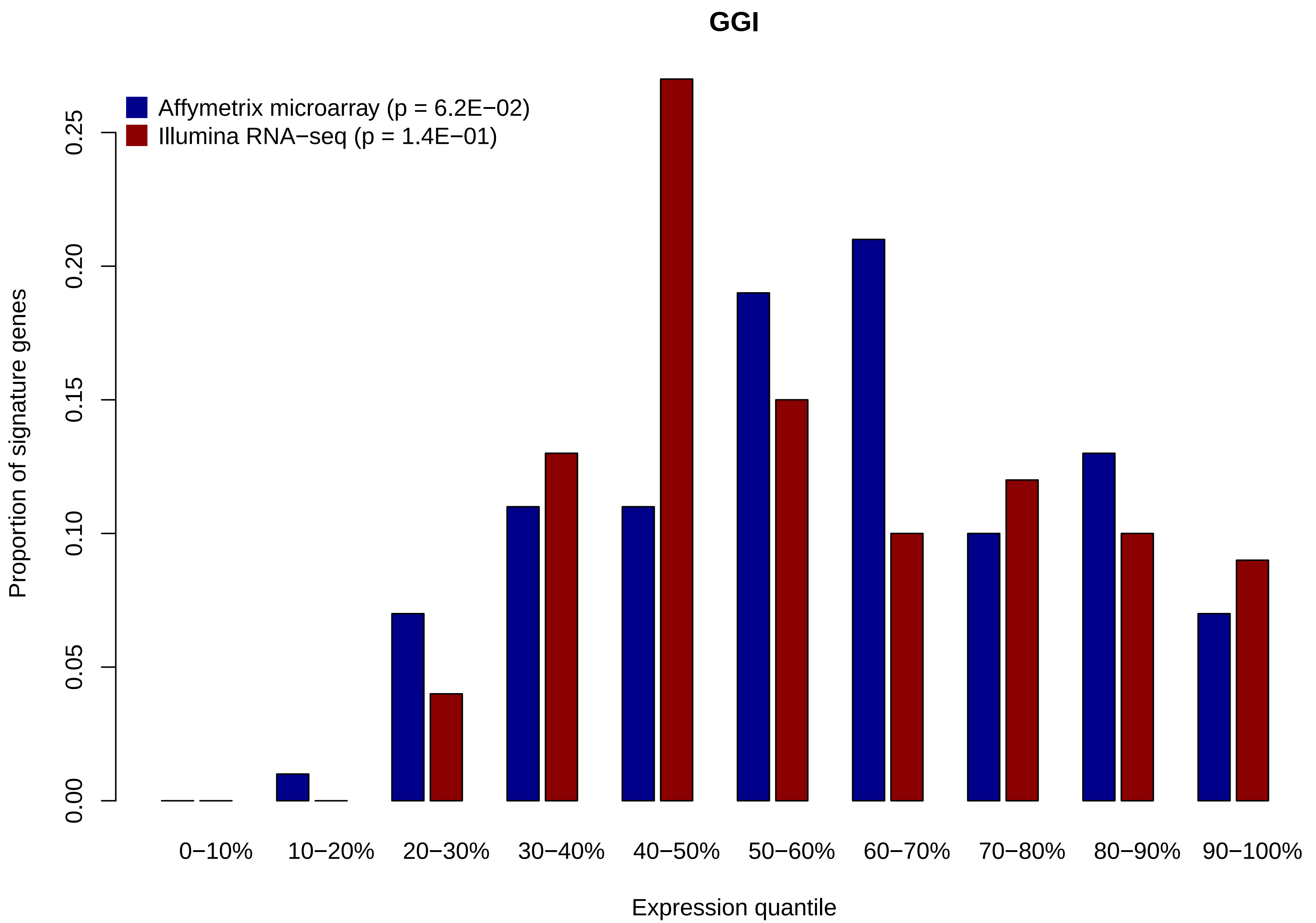
GGI
Affymetrix microarray (p = 6.2E−02)
Illumina RNA−seq (p = 1.4E−01)
Proportion of signature genes
0.00
0.05
0.10
0.15
0.20
0.25
0−10%
10−20%
20−30%
30−40%
40−50%
50−60%
60−70%
70−80%
80−90%
90−100%
Expression quantile

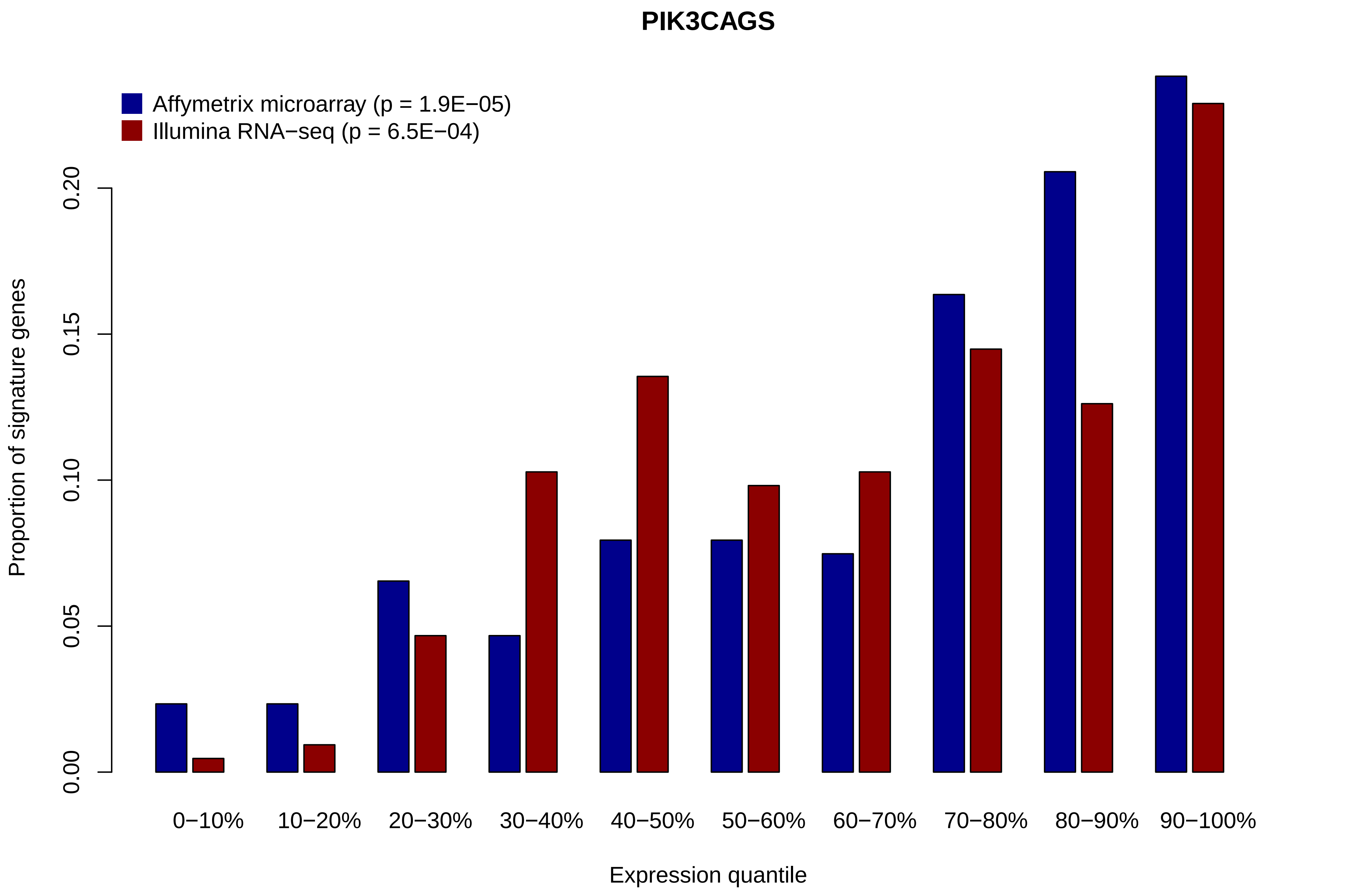
PIK3CAGS
Affymetrix microarray (p = 1.9E−05)
Illumina RNA−seq (p = 6.5E−04)
Proportion of signature genes
0.00
0.05
0.10
0.15
0.20
0−10%
10−20%
20−30%
30−40%
40−50%
50−60%
60−70%
70−80%
80−90%
90−100%
Expression quantile

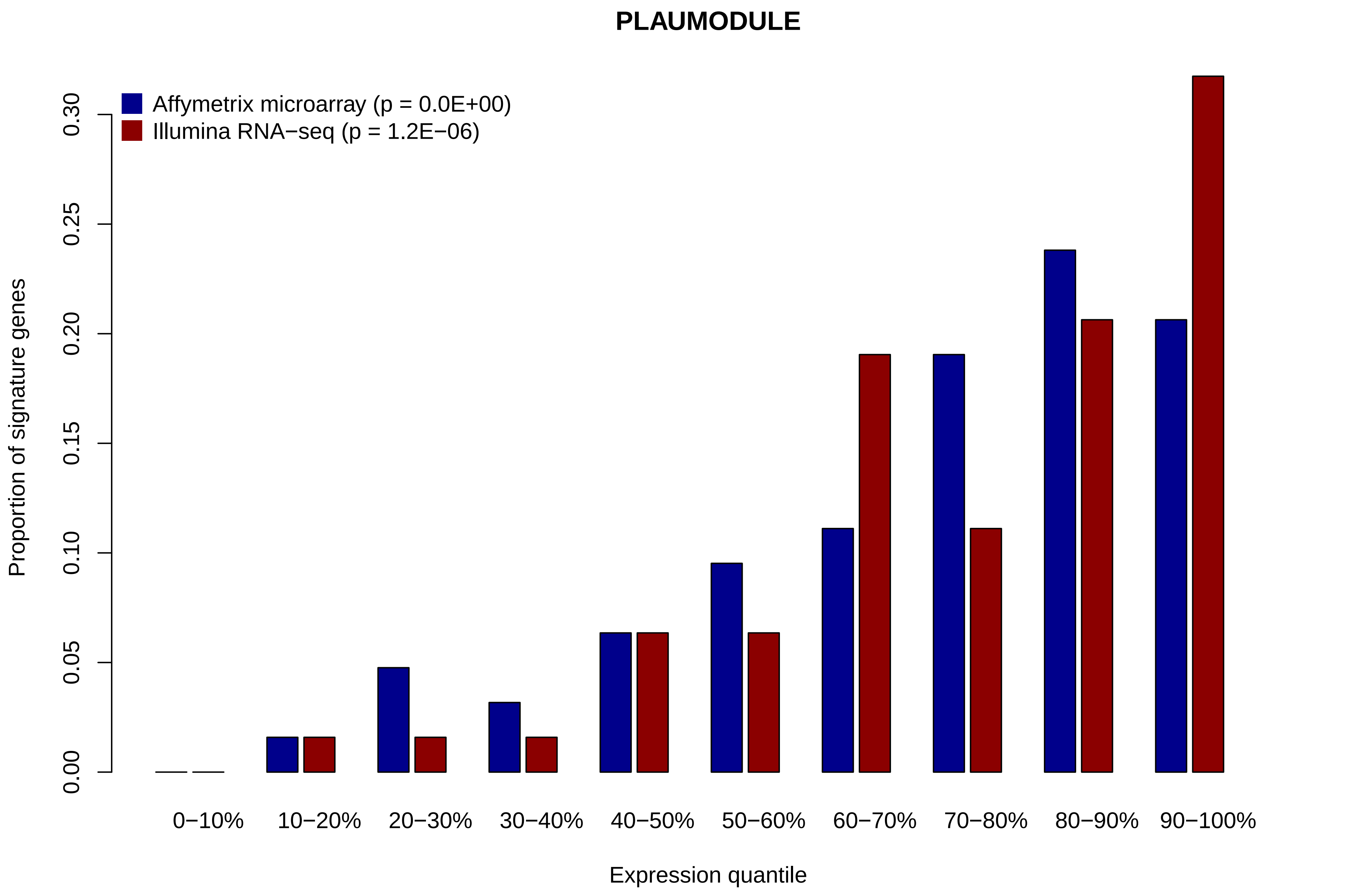
PLAUMODULE
Affymetrix microarray (p = 0.0E+00)
Illumina RNA−seq (p = 1.2E−06)
Proportion of signature genes
0.00
0.05
0.10
0.15
0.20
0.25
0.30
0−10%
10−20%
20−30%
30−40%
40−50%
50−60%
60−70%
70−80%
80−90%
90−100%
Expression quantile

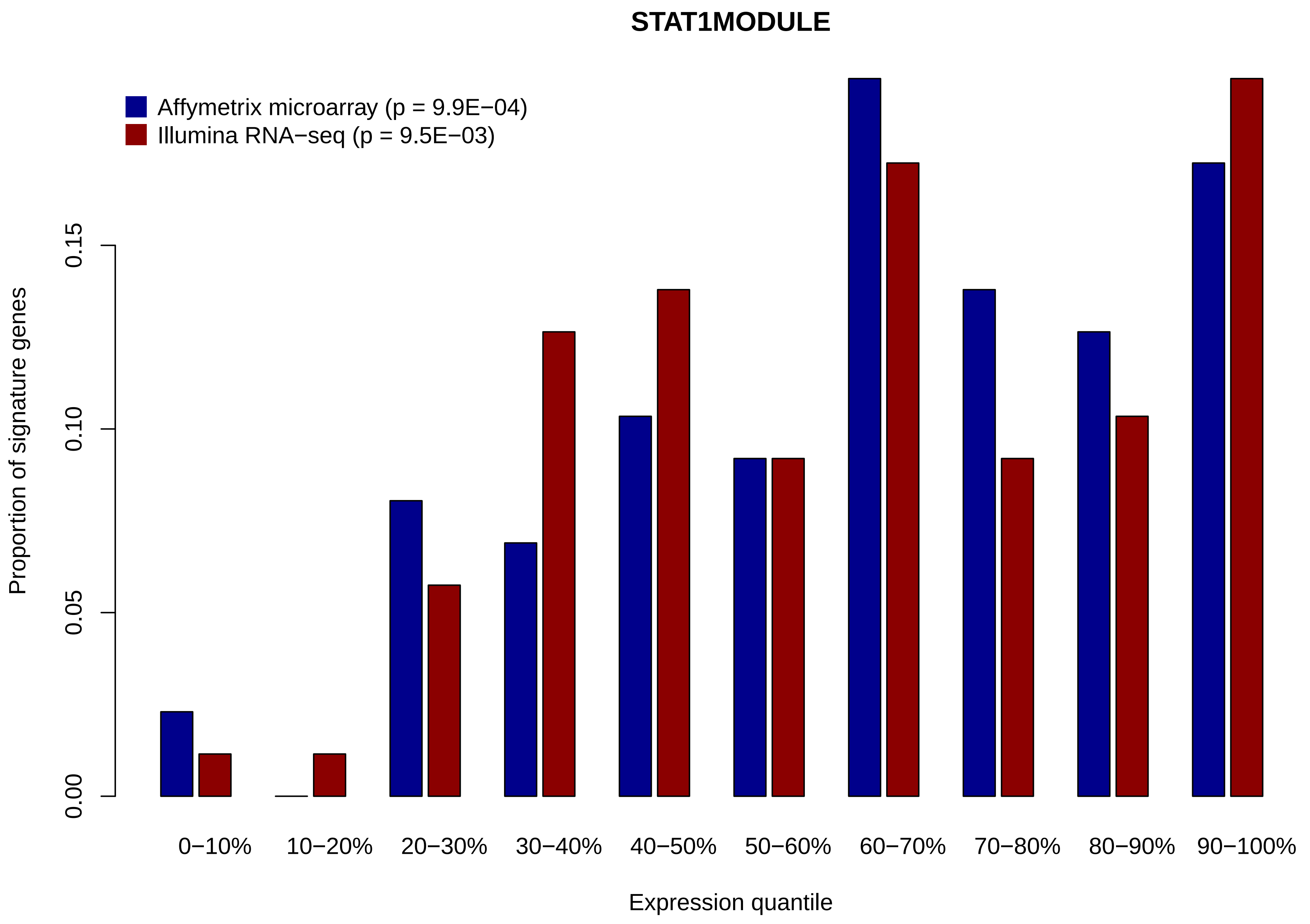
STAT1MODULE
Affymetrix microarray (p = 9.9E−04)
Illumina RNA−seq (p = 9.5E−03)
Proportion of signature genes
0.00
0.05
0.10
0.15
0−10%
10−20%
20−30%
30−40%
40−50%
50−60%
60−70%
70−80%
80−90%
90−100%
Expression quantile

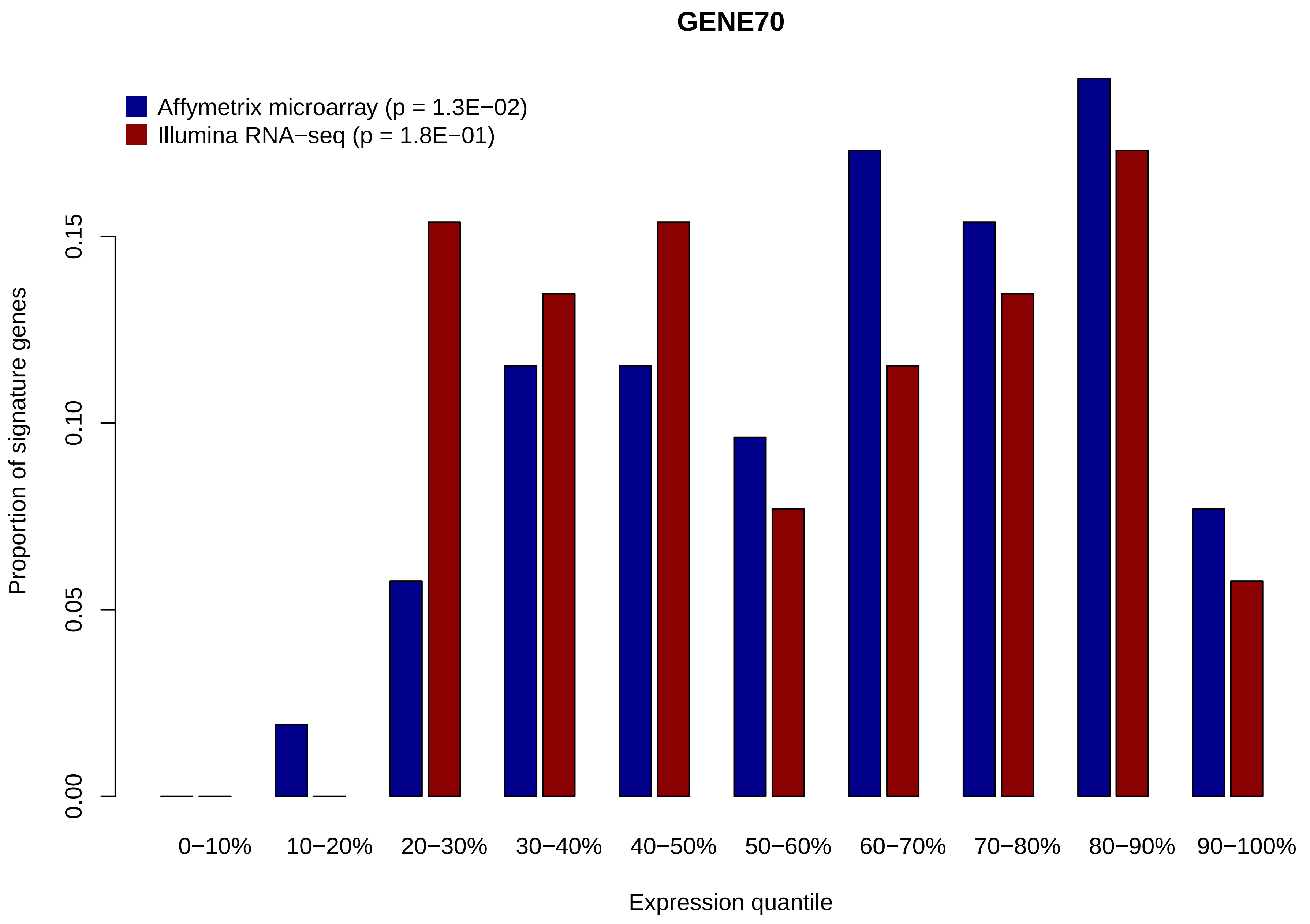

GENE70
Affymetrix microarray (p = 1.3E−02)
Illumina RNA−seq (p = 1.8E−01)
Proportion of signature genes
0.00
0.05
0.10
0.15
0−10%
10−20%
20−30%
30−40%
40−50%
50−60%
60−70%
70−80%
80−90%
90−100%
Expression quantile

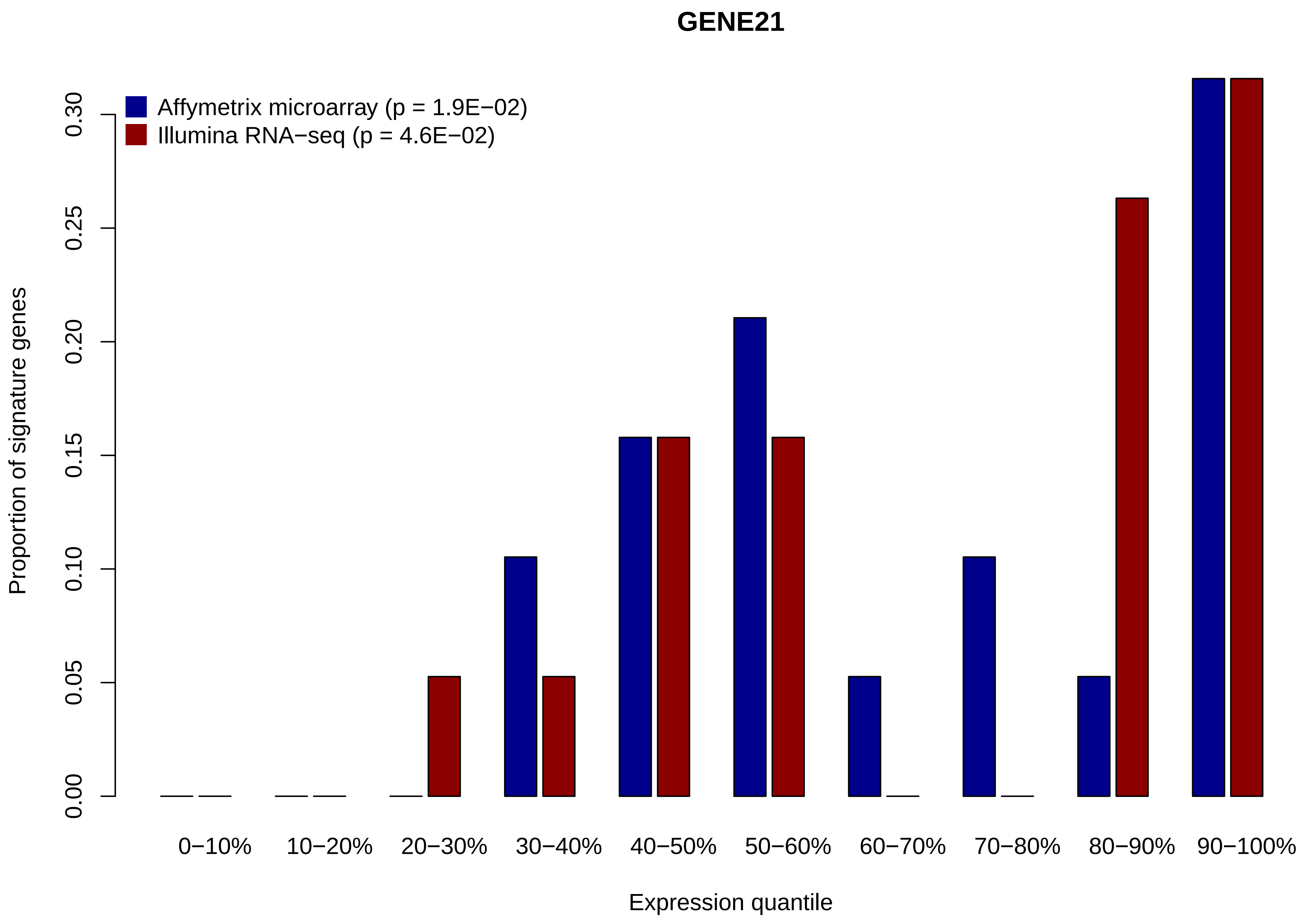
GENE21
Affymetrix microarray (p = 1.9E−02)
Illumina RNA−seq (p = 4.6E−02)
Proportion of signature genes
0.00
0.05
0.10
0.15
0.20
0.25
0.30
0−10%
10−20%
20−30%
30−40%
40−50%
50−60%
60−70%
70−80%
80−90%
90−100%
Expression quantile

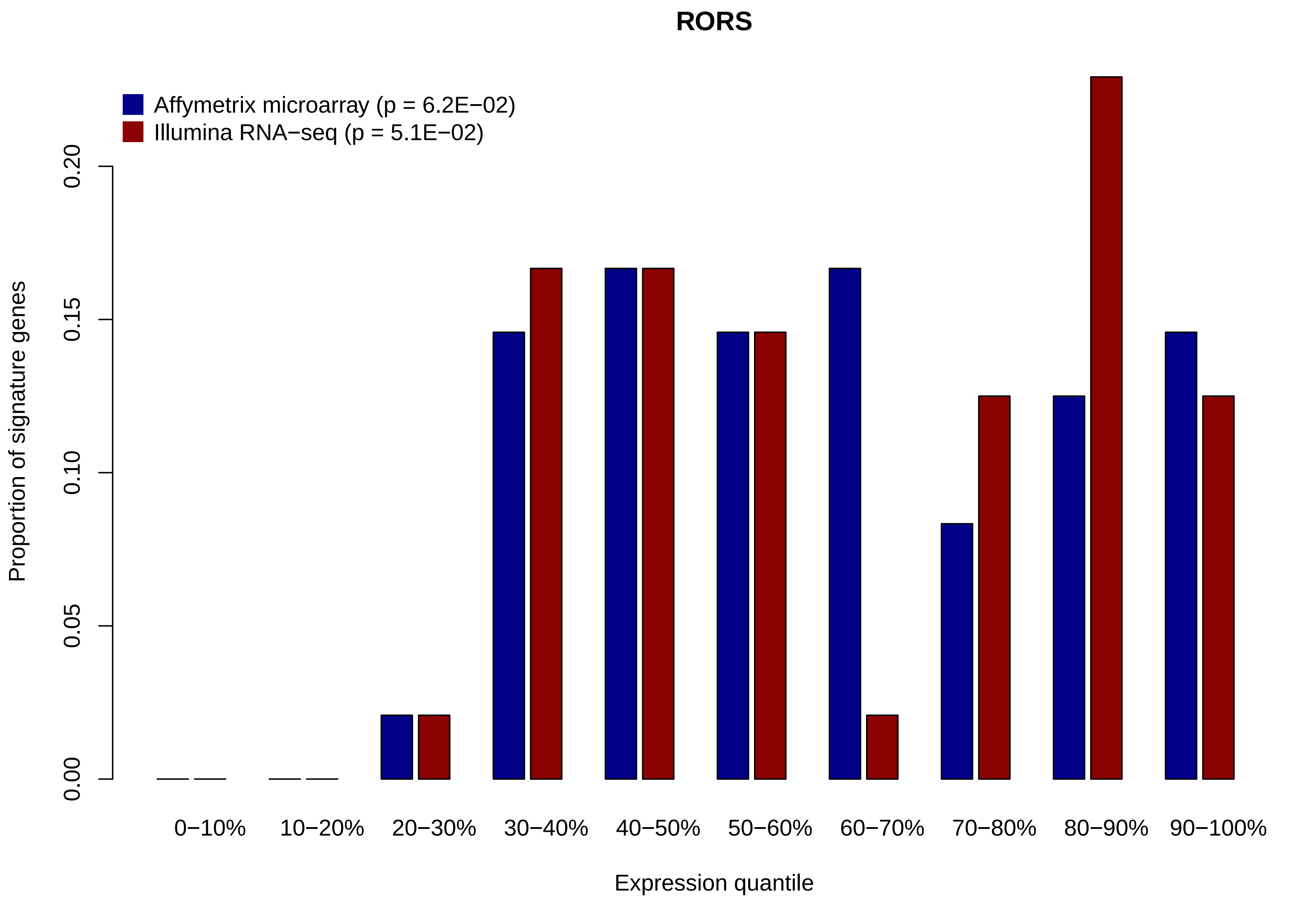

RORS
Affymetrix microarray (p = 6.2E−02)
Illumina RNA−seq (p = 5.1E−02)
Proportion of signature genes
0.00
0.05
0.10
0.15
0.20
0−10%
10−20%
20−30%
30−40%
40−50%
50−60%
60−70%
70−80%
80−90%
90−100%
Expression quantile

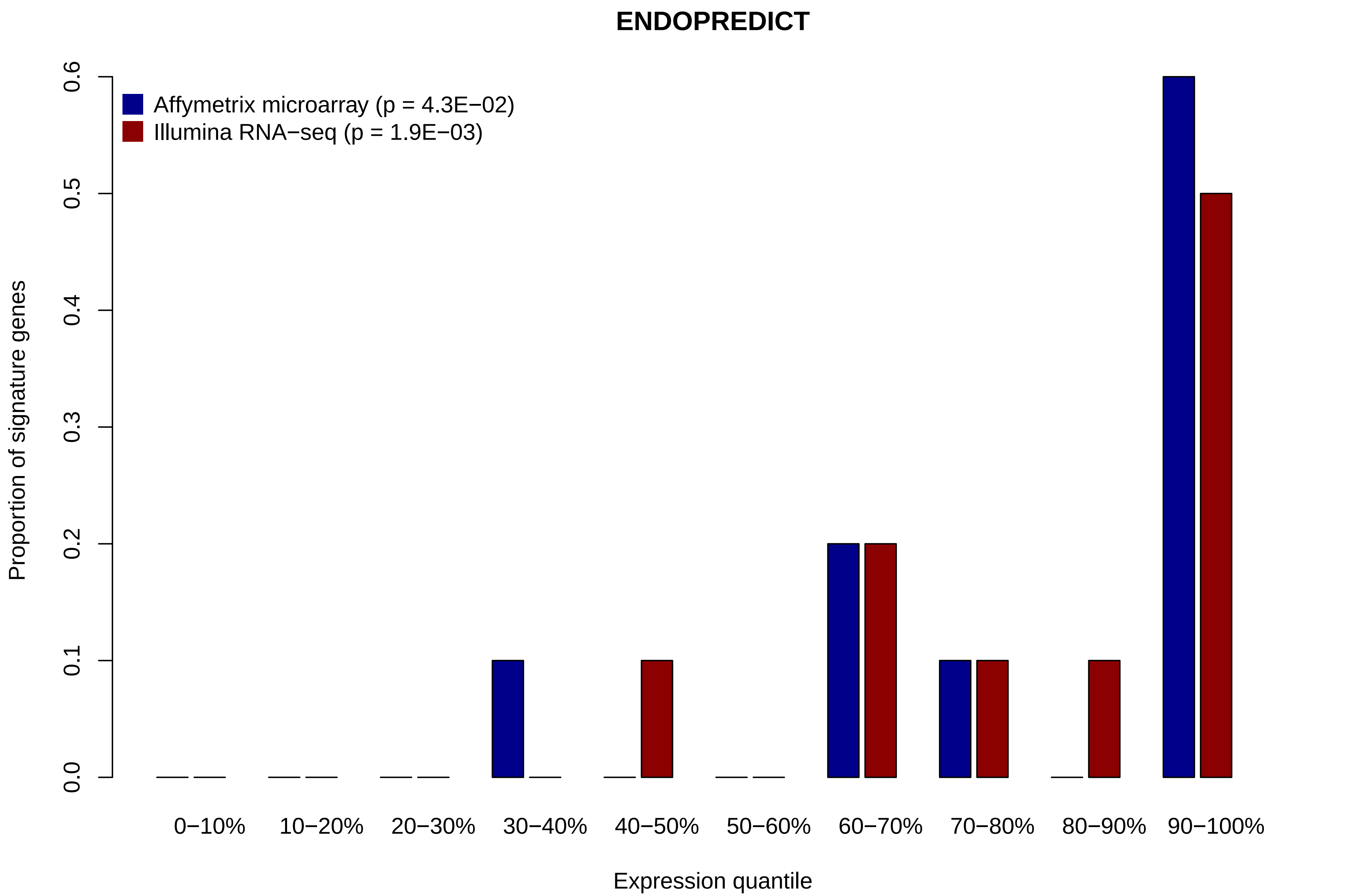
ENDOPREDICT
Affymetrix microarray (p = 4.3E−02)
Illumina RNA−seq (p = 1.9E−03)
Proportion of signature genes
0.0
0.1
0.2
0.3
0.4
0.5
0.6
0−10%
10−20%
20−30%
30−40%
40−50%
50−60%
60−70%
70−80%
80−90%
90−100%
Expression quantile

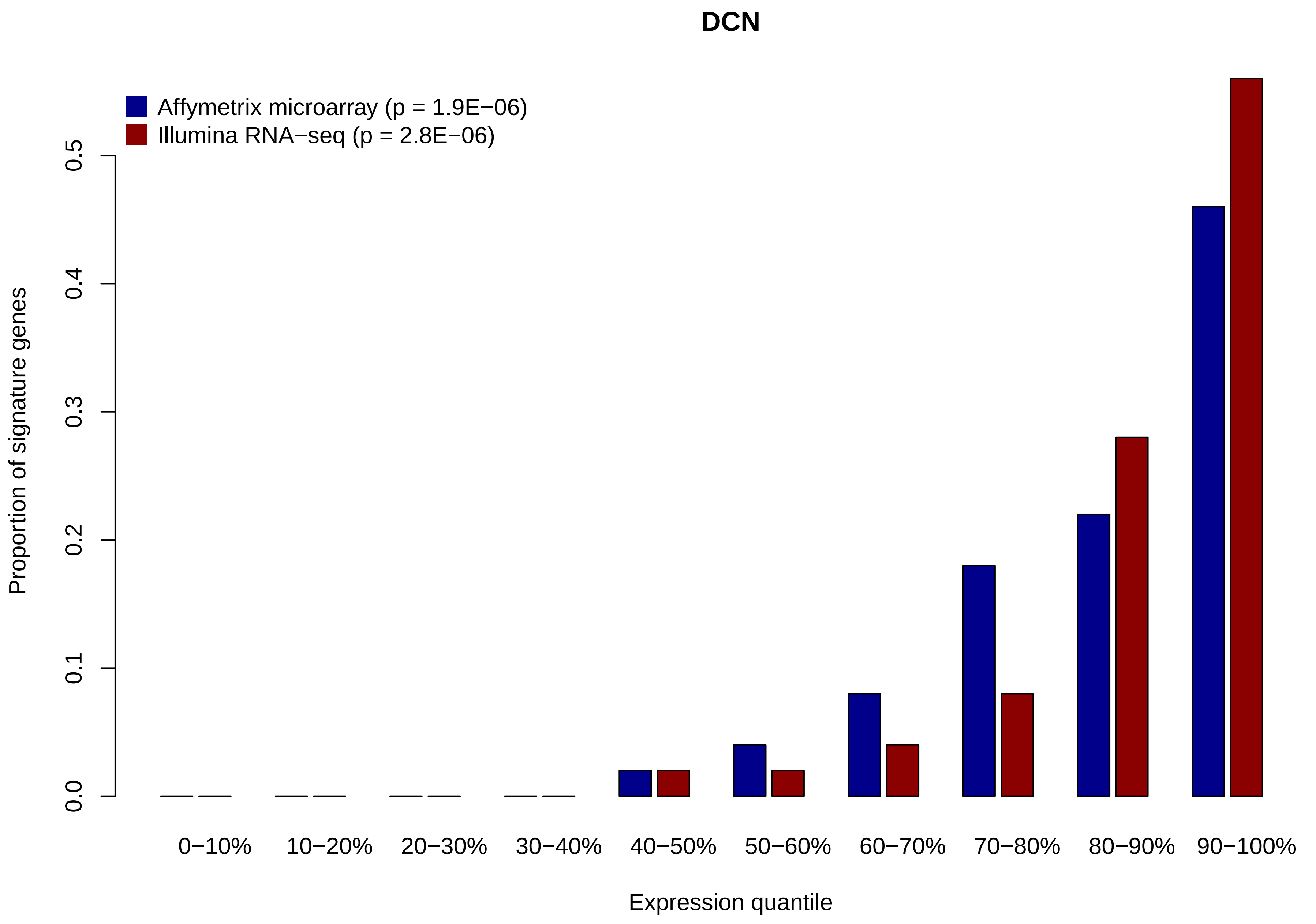
DCN
Affymetrix microarray (p = 1.9E−06)
Illumina RNA−seq (p = 2.8E−06)
Proportion of signature genes
0.0
0.1
0.2
0.3
0.4
0.5
0−10%
10−20%
20−30%
30−40%
40−50%
50−60%
60−70%
70−80%
80−90%
90−100%
Expression quantile

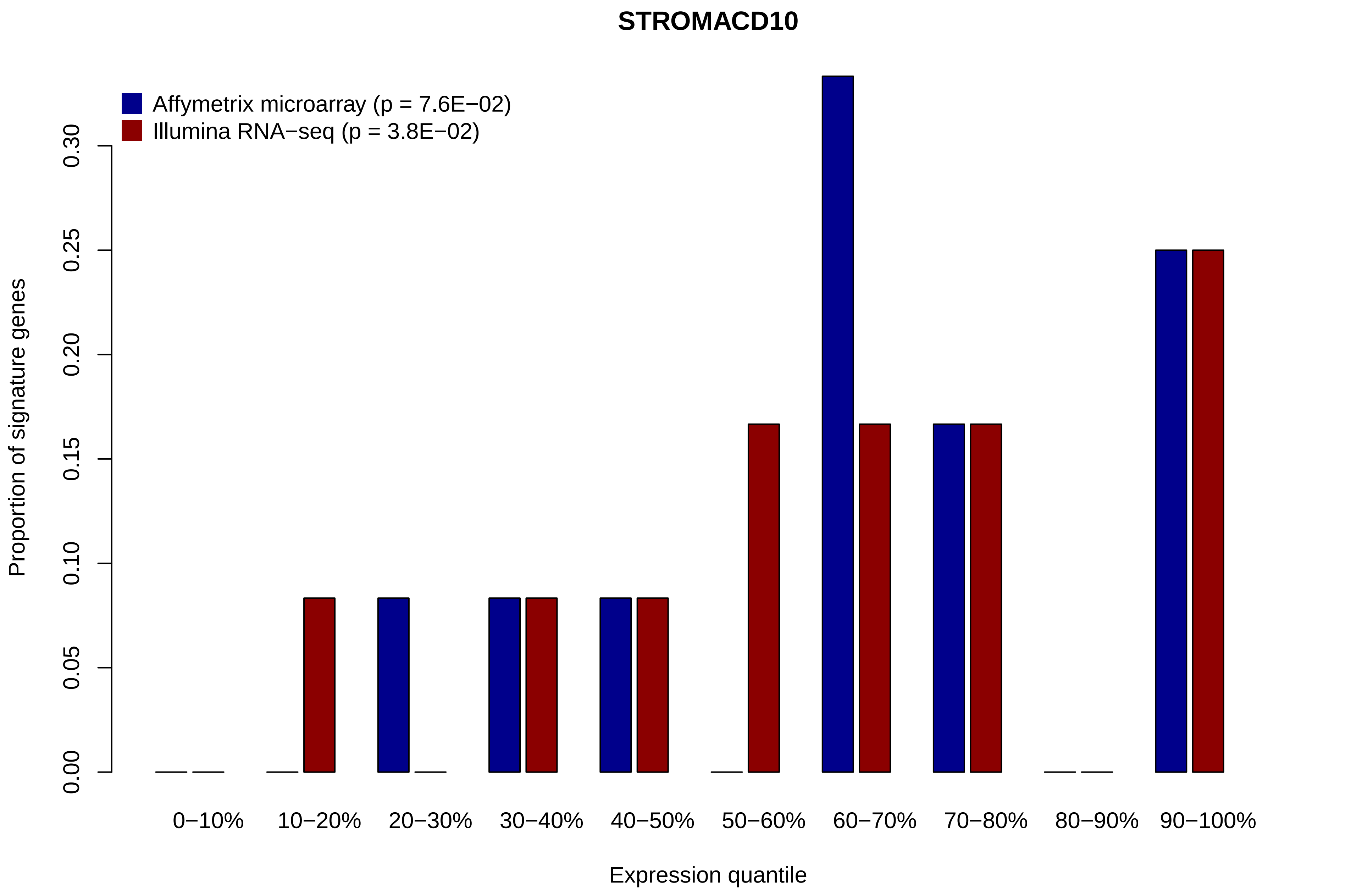
STROMACD10
Affymetrix microarray (p = 7.6E−02)
Illumina RNA−seq (p = 3.8E−02)
Proportion of signature genes
0.00
0.05
0.10
0.15
0.20
0.25
0.30
0−10%
10−20%
20−30%
30−40%
40−50%
50−60%
60−70%
70−80%
80−90%
90−100%
Expression quantile

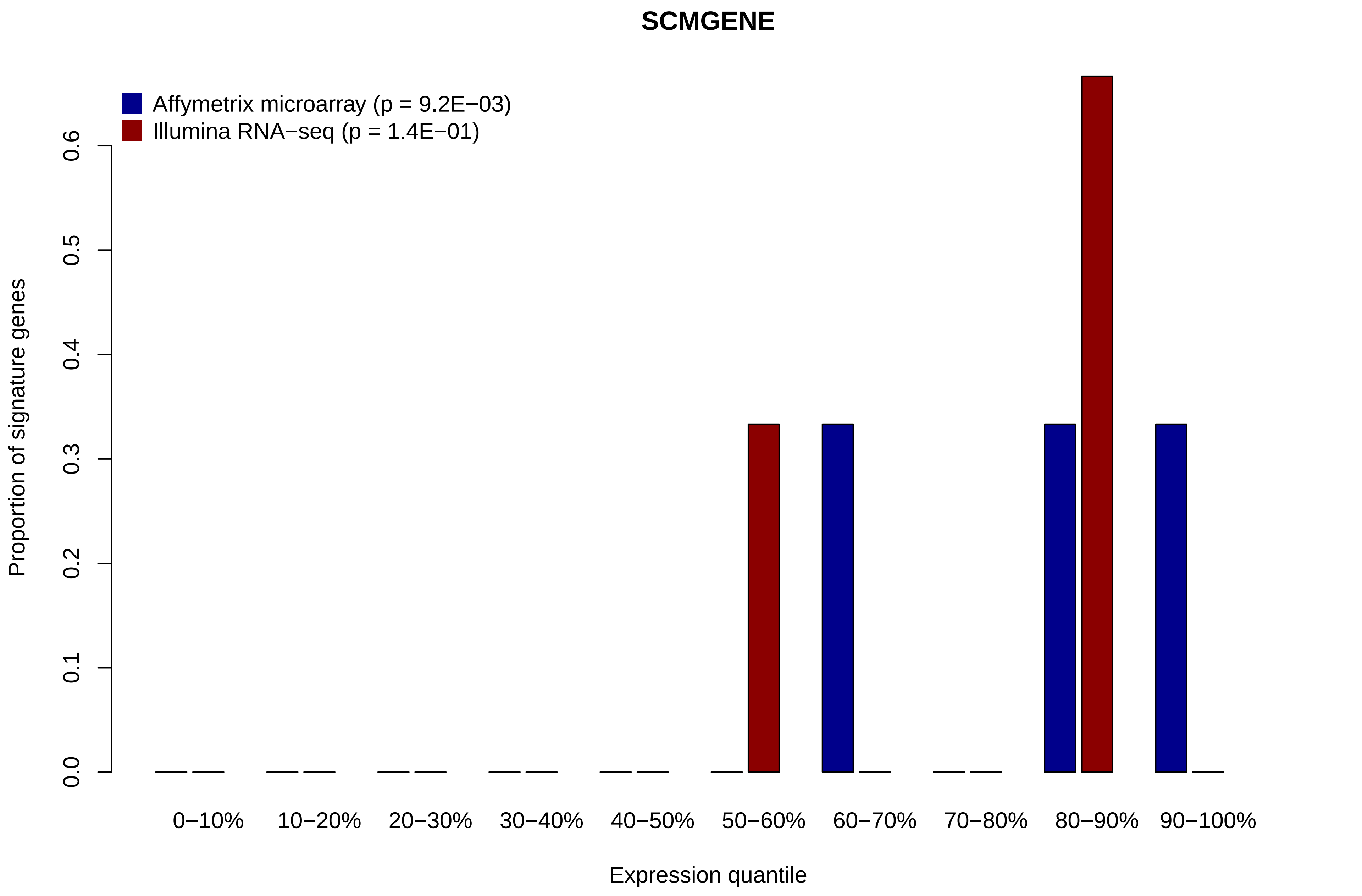
SCMGENE
Affymetrix microarray (p = 9.2E−03)
Illumina RNA−seq (p = 1.4E−01)
Proportion of signature genes
0.0
0.1
0.2
0.3
0.4
0.5
0.6
0−10%
10−20%
20−30%
30−40%
40−50%
50−60%
60−70%
70−80%
80−90%
90−100%
Expression quantile

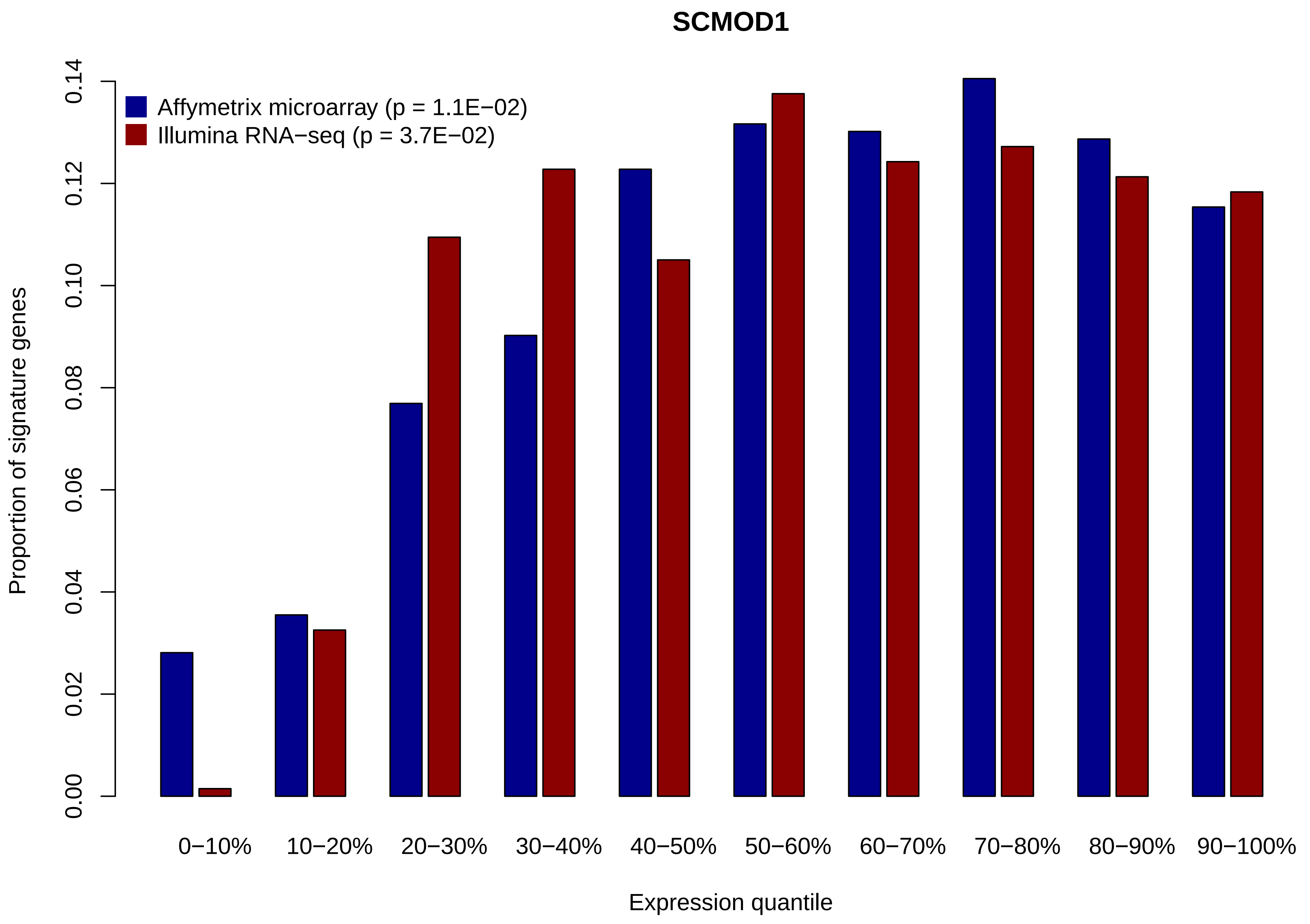
SCMOD1
Affymetrix microarray (p = 1.1E−02)
Illumina RNA−seq (p = 3.7E−02)
Proportion of signature genes
0.00
0.02
0.04
0.06
0.08
0.10
0.12
0.14
0−10%
10−20%
20−30%
30−40%
40−50%
50−60%
60−70%
70−80%
80−90%
90−100%
Expression quantile

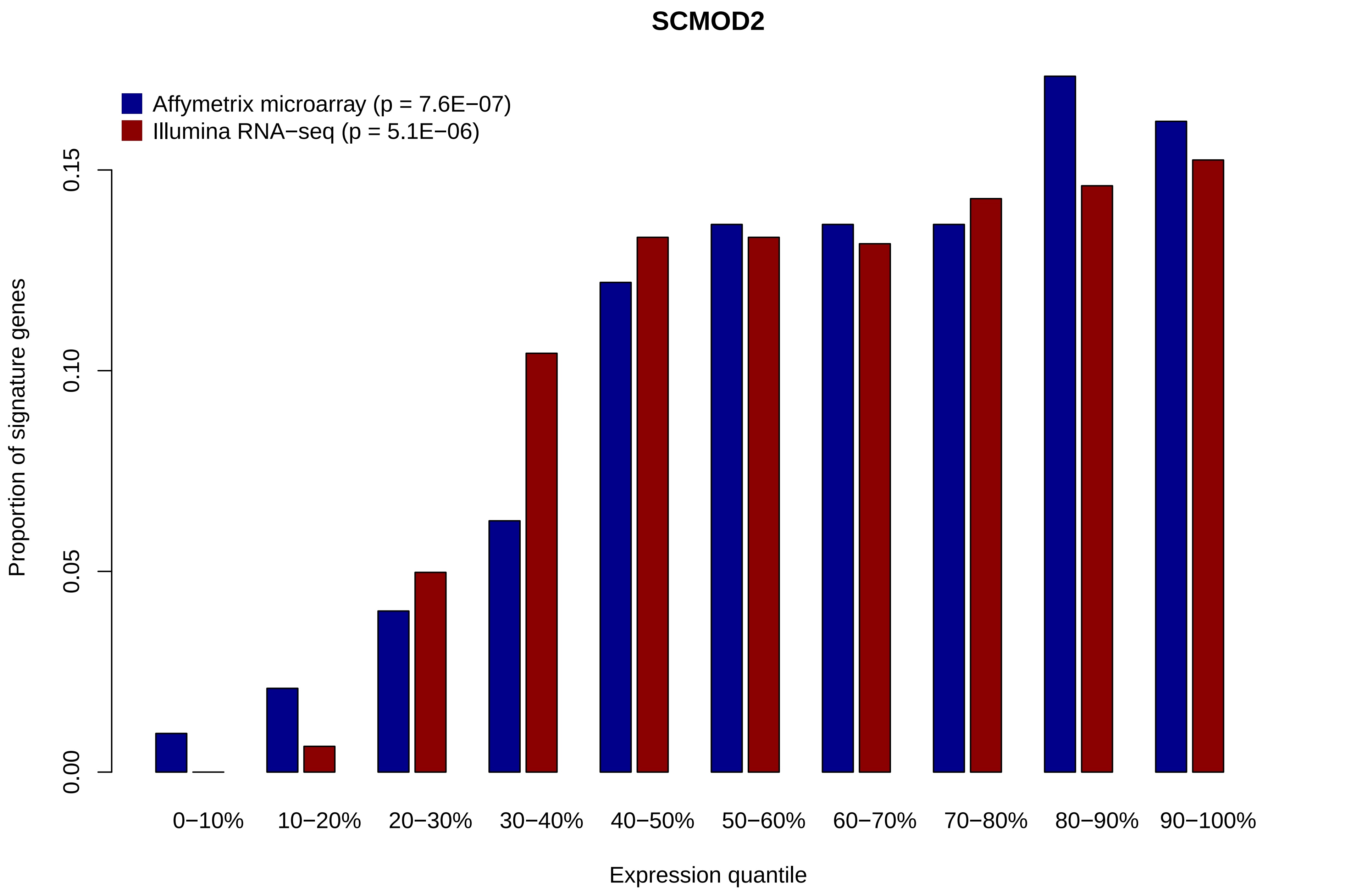
SCMOD2
Affymetrix microarray (p = 7.6E−07)
Illumina RNA−seq (p = 5.1E−06)
Proportion of signature genes
0.00
0.05
0.10
0.15
0−10%
10−20%
20−30%
30−40%
40−50%
50−60%
60−70%
70−80%
80−90%
90−100%
Expression quantile

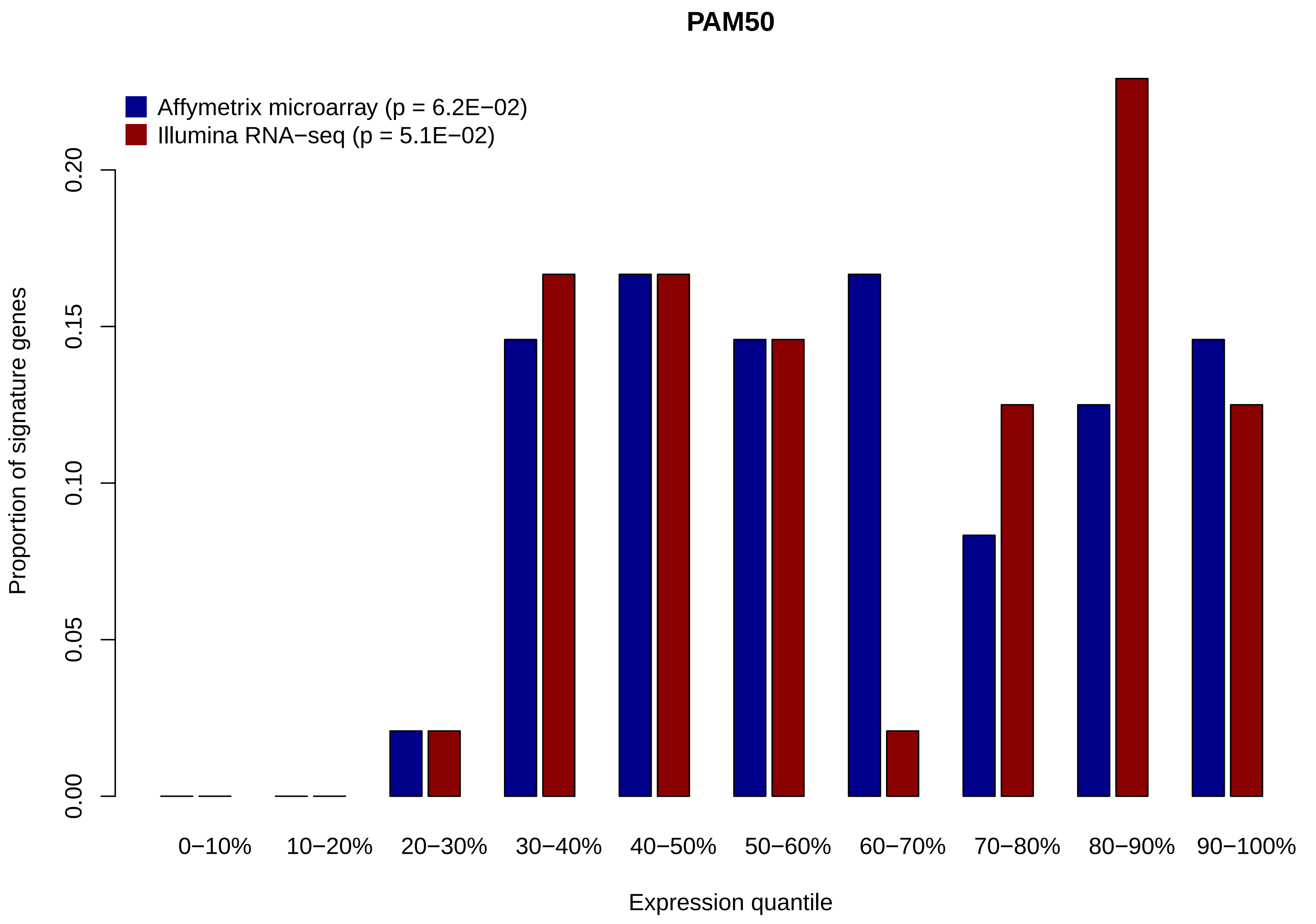
PAM50
Affymetrix microarray (p = 6.2E−02)
Illumina RNA−seq (p = 5.1E−02)
Proportion of signature genes
0.00
0.05
0.10
0.15
0.20
0−10%
10−20%
20−30%
30−40%
40−50%
50−60%
60−70%
70−80%
80−90%
90−100%
Expression quantile

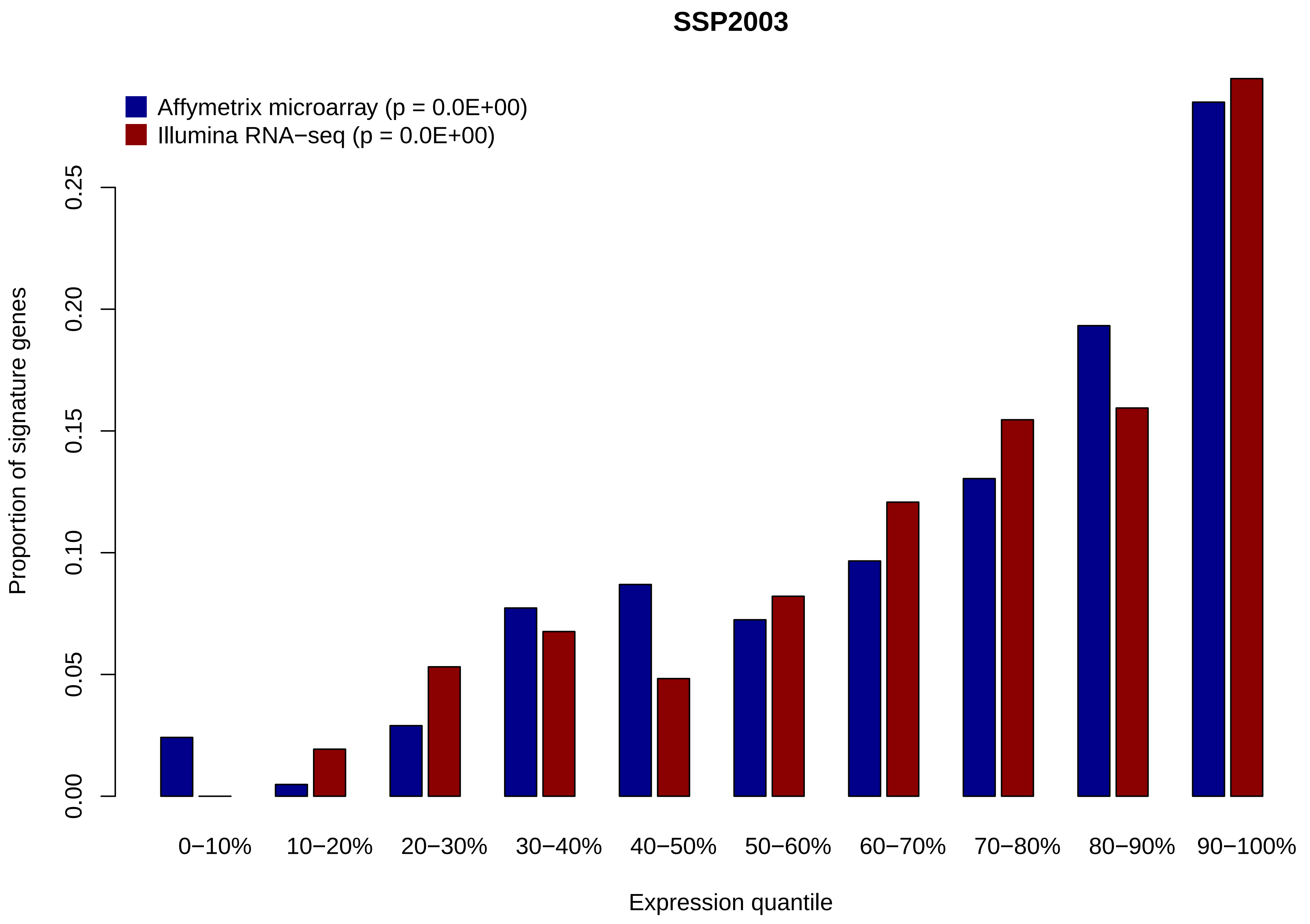
SSP2003
Affymetrix microarray (p = 0.0E+00)
Illumina RNA−seq (p = 0.0E+00)
Proportion of signature genes
0.00
0.05
0.10
0.15
0.20
0.25
0−10%
10−20%
20−30%
30−40%
40−50%
50−60%
60−70%
70−80%
80−90%
90−100%
Expression quantile

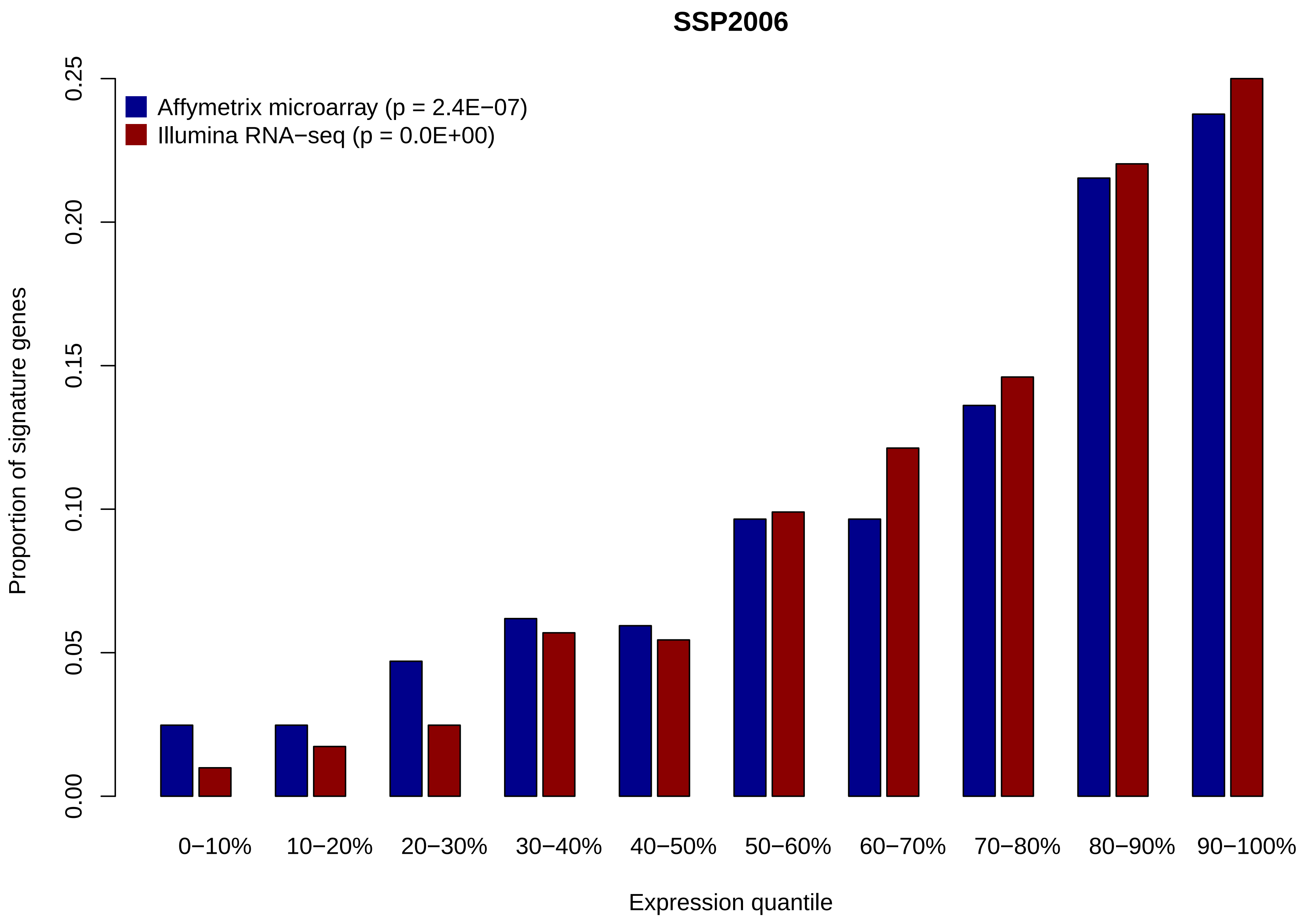
SSP2006
Affymetrix microarray (p = 2.4E−07)
Illumina RNA−seq (p = 0.0E+00)
Proportion of signature genes
0.00
0.05
0.10
0.15
0.20
0.25
0−10%
10−20%
20−30%
30−40%
40−50%
50−60%
60−70%
70−80%
80−90%
90−100%
Expression quantile
